# Dual CARM1-and IKZF3-targeting: A novel approach to multiple myeloma therapy

**DOI:** 10.1016/j.omton.2025.200966

**Published:** 2025-03-28

**Authors:** Wei Ni, Swati Garg, Basudev Chowdhury, Martin Sattler, Dana Sanchez, Chengcheng Meng, Taisei Akatsu, Katherine A. Donovan, Jun Qi, Michelle Y. Wang, Cara Ann Starnbach, Xiaoxi Liu, Maria Tarazona Guzman, Wei Pin Teh, Richard Stone, James D. Griffin, Sara Buhrlage, Ellen Weisberg

## Main text

(Molecular Therapy: Oncology *33*, 200952; March 2025)

In the originally published version of the article, a short title (“Synergy between CARM1 inhibition and IMIDs”) under the actual title was merged with the actual title. The correct title for this paper is “Dual CARM1- and IKZF3-targeting: a novel approach to multiple myeloma therapy.” The short title was included by accident, and unfortunately, this mistake was not caught during the proof stage. The authors and editors apologize for this mistake.

