# Supplementary material for: Dual CARM1-and IKZF3-targeting: A novel approach to multiple myeloma therapy synergy between CARM1 inhibition and IMiDs
Source: Mol Ther Oncol. 2025 Feb 20;33(1):200952. doi: 10.1016/j.omton.2025.200952 (PMC11930131; doi:10.1016/j.omton.2025.200952)
Supplement: Document S1. Figures S1–S37 [file mmc1.pdf]

OMTON, Volume *33*

Supplemental information

Dual CARM1-and IKZF3-targeting: A novel approach to multiple myeloma therapy synergy between CARM1 inhibition and IMiDs

Wei Ni, Swati Garg, Basudev Chowdhury, Martin Sattler, Dana Sanchez, Chengcheng Meng, Taisei Akatsu, Katherine A. Donovan, Jun Qi, Michelle Y. Wang, Cara Ann Starnbach, Xiaoxi Liu, Maria Tarazona Guzman, Wei Pin Teh, Richard Stone, James D. Griffin, Sara Buhrlage, and Ellen Weisberg

Supplemental Materials and Methods

Table S1 Raw proteomics data for the 8-hour time point corresponding to 074 treatment of MOLT4 cells. Multiplexed quantitative proteomics experiment used for assessing protein abundance changes in response to degrader treatment.

Table S2 Raw proteomics data for the 24-hour time point corresponding to 074 treatment of MOLT4 cells. Multiplexed quantitative proteomics experiment used for assessing protein abundance changes in response to degrader treatment.

Table S3 Enzyme assay assessing 074 inhibition of CARM1.

Quantitative PCR (qPCR)

The following primers were used for qPCR.

Gene	Forward primer (5'->3')	Reverse primer (5'->3')	Ampicon (bp)
MYC	AATGAAAAGGCCCCCAAGGTAGTTATCC	GTCGTTTCCGCAACAAGTCCTCTTC	112
IRF4	CCCGGAAATCCCGTACCAAT	TTATGCTTGGCTCTGTGGGG	158

074 Chemical Synthesis

General Experimental and Materials:

All commercially available starting materials and solvents were purchased from Sigma Aldrich, Fisher Scientific, Oakwood Chemical, and Combi Blocks and were used without further purification. Deuterated solvents ($CDCl_3$, DMSO-d^6) were obtained from Cambridge Isotope Laboratories. Compound purification was performed by normal phase column chromatography using Teledyne Combi Flash chromatography system, and/or reversed phase chromatography using Shimadzu Nexera LC40 Series with Shim-Pack GIS 10 μm C18 30x250 mm column. Reaction monitoring and mass spectrometry data were obtained using Waters ACQUITY UPLC I-Class system. ^{1}H-NMR spectra were recorded at 298K using a Bruker ARX 500 (500 MHz) spectrometer.

General Information for Starting Materials:

All starting materials were purchased from commercial sources and used without further purification unless otherwise specified. Intermediates **1**, **3**, and **5** were synthesized according to literature procedure and the spectroscopic data were consistent with the reported values[1-3].

<u>**Supplemental Figures**</u>

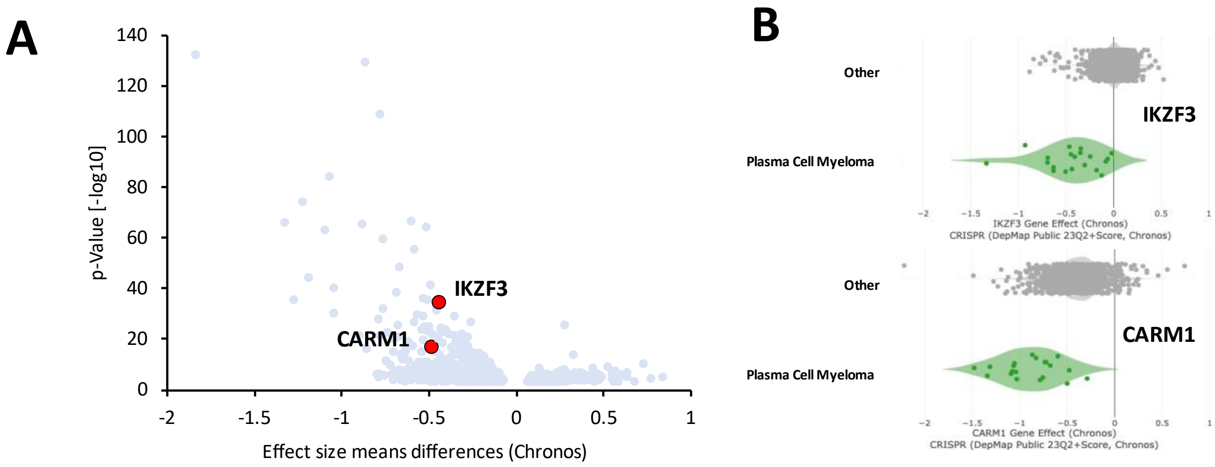

Figure S1. Genome-based CRISPR gene-editing screen identifies preferential dependencies in myeloma cell lines. A. Volcano plot of Chronos dependency scores and p-values within the DepMap dataset for myeloma identify IKZF3 and CARM1 as primary dependencies. B. Gene effect of IKZF3 and CARM1 targeting in 20 myeloma cell lines versus various other cancer cell lines tested within this screen. Data were retrieved from https://depmap.org/.

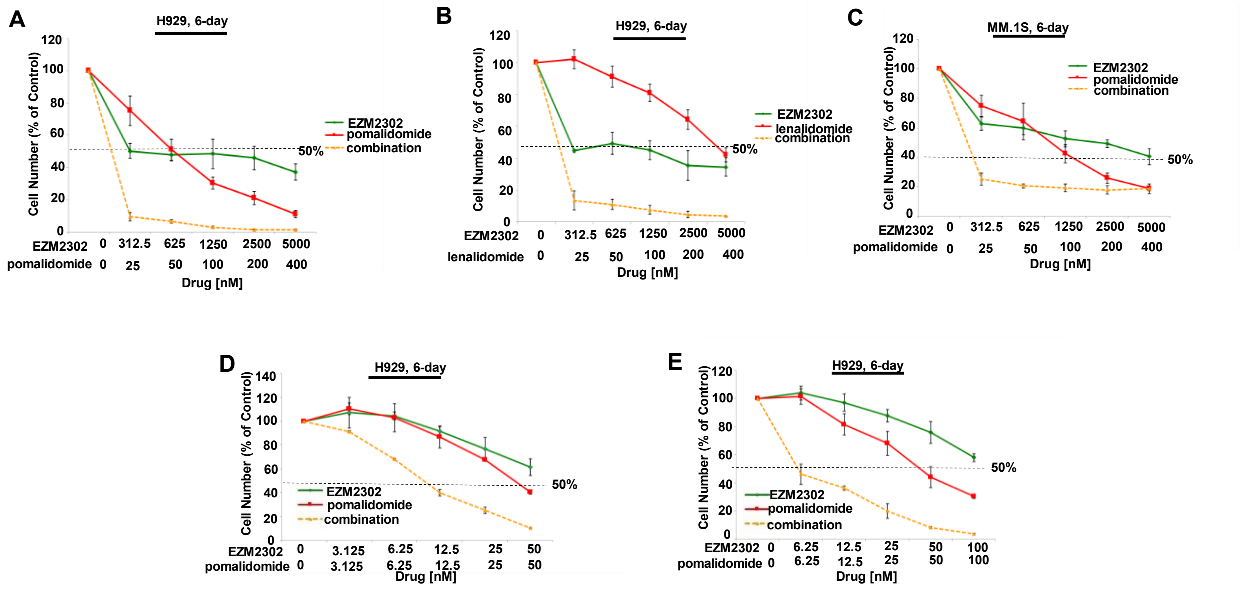

Figure S2. CARM1 inhibition potentiates the antiproliferative effects of pomalidomide against MM cells. (A) Proliferation assay: 6-day treatment of H929 cells with EZM2302, pomalidomide, or a combination. (B) Proliferation assay: 6-day treatment of H929 cells with EZM2302, lenalidomide, or a combination. (C) Proliferation assay: 6-day treatment of MM.1S cells with EZM2302, pomalidomide,or a combination. (D) Proliferation assay: 6-day treatment of H929 cells with EZM2302, pomalidomide, or a combination (concentration range 3.125-50 nM). (E) Proliferation assay: 6-day treatment of H929 cells with EZM2302, pomalidomide, or a combination (concentration range 6.25-100 nM).

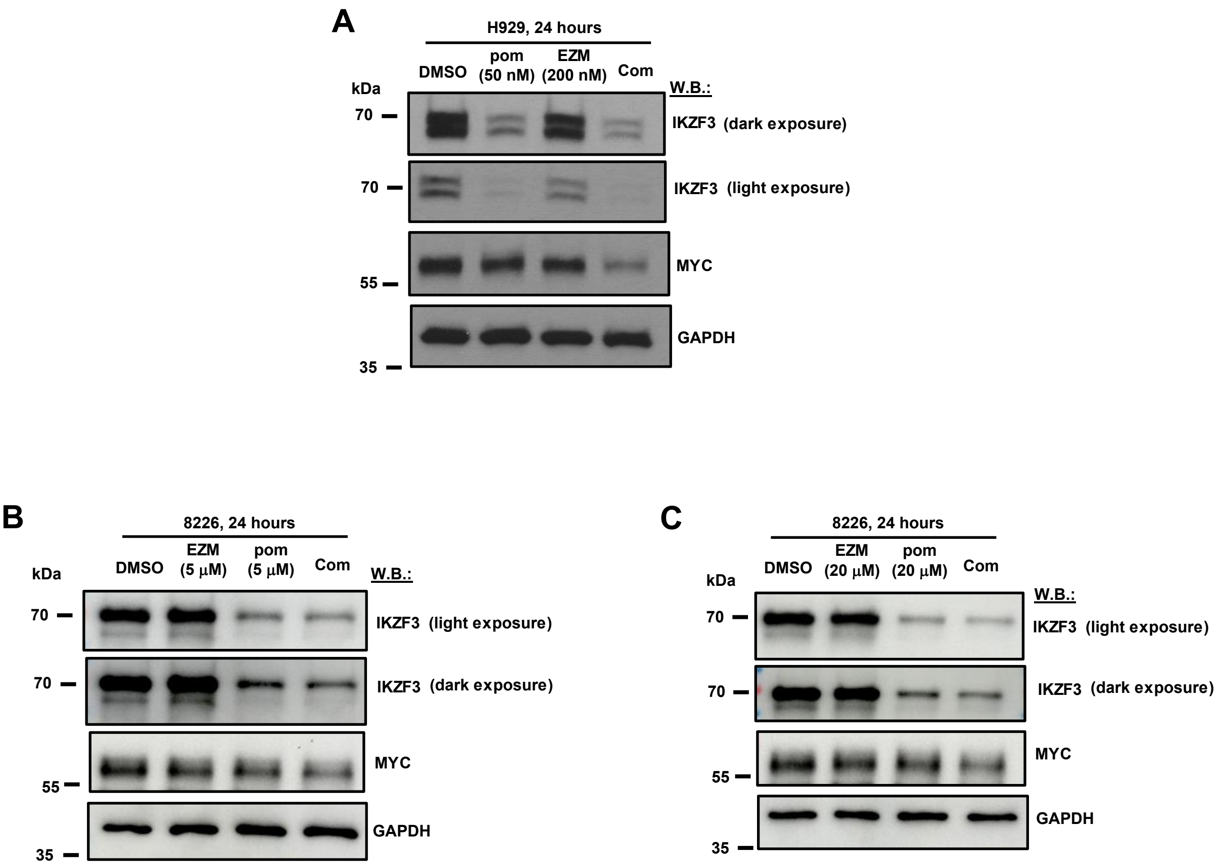

Figure S3 (A-C). Effects of CARM1 targeting and IMiD treatment on IKZF3 and MYC in MM cells. (A-C) Immunoblots: Effects of 24-hour treatment of H929 cells (A) or 8226 cells (B-C) with pomalidomide, EZM2302, or a combination on IKZF3 and MYC expression.

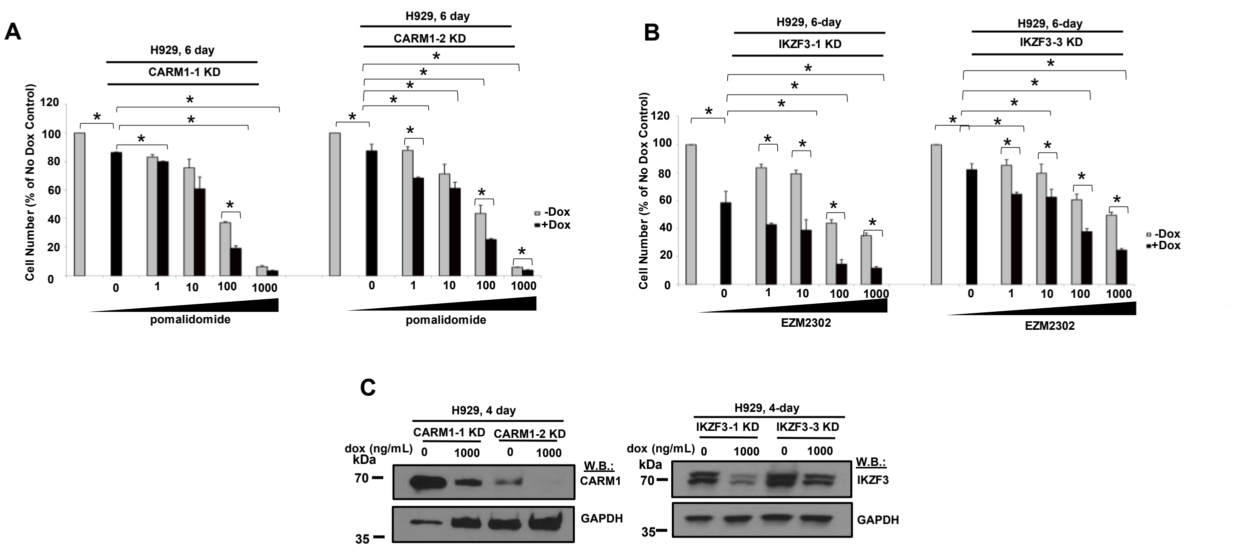

Figure S4. Contribution of CARM1 and IKZF3 to synergy between EZM2302 and pomalidomide. For doxycycline-inducible KD of *CARM1* and *IKZF3*, two individual clones were generated for each gene: ishCARM1-1 (CARM1-1 KD) and ishCARM1-2 for CARM1 (CARM1-2 KD), and ishIKZF3-1 (IKZF3-1 KD) and ishIKZF3-3 (IKZF3-3 KD) for IKZF3. (A) Effects of doxycycline-inducible CARM1 KD on potency of pomalidomide against MM cell growth. (B) Effects of doxycycline-inducible IKZF3 KD on potency of CARM1 inhibition against MM cell growth. (C) Immunoblots: (Left panel) Assessment of CARM1 KD efficiency in doxycycline-treated H929 CARM1 KD cells following a 4-day treatment. (Right panel): Immunoblots: Assessment of IKZF3 KD efficiency in doxycycline-treated H929 IKZF3 KD cells following a 4-day treatment.

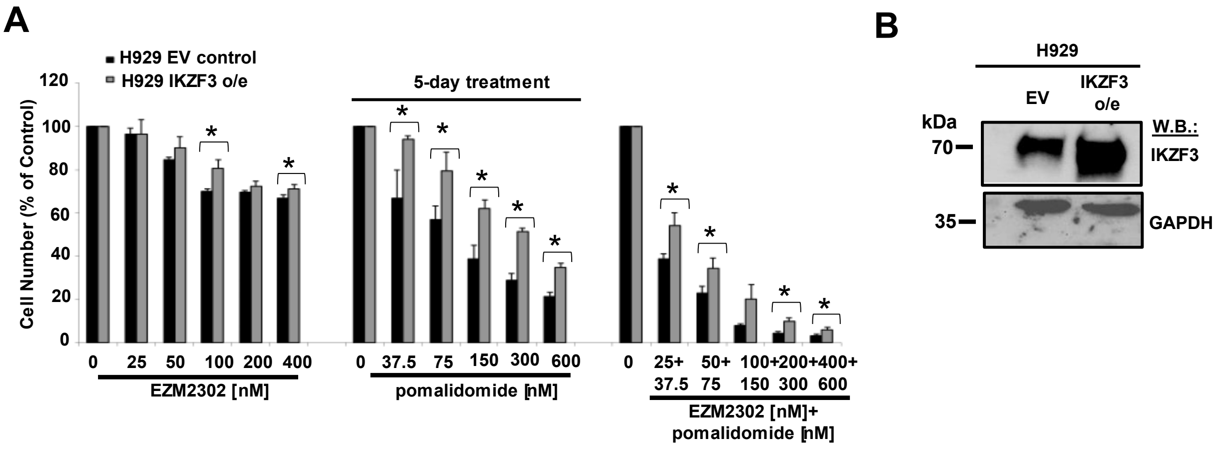

Figure S5. Contribution of IKZF3 to synergy between EZM2302 and pomalidomide. (A) Effects of IKZF3 overexpression (IKZF3 o/e) in MM cells on EZM2302, pomalidomide, or a combination. (B) Immunoblots: Assessment of IKZF3 levels in H929 cells engineered to overexpress IKZF3.

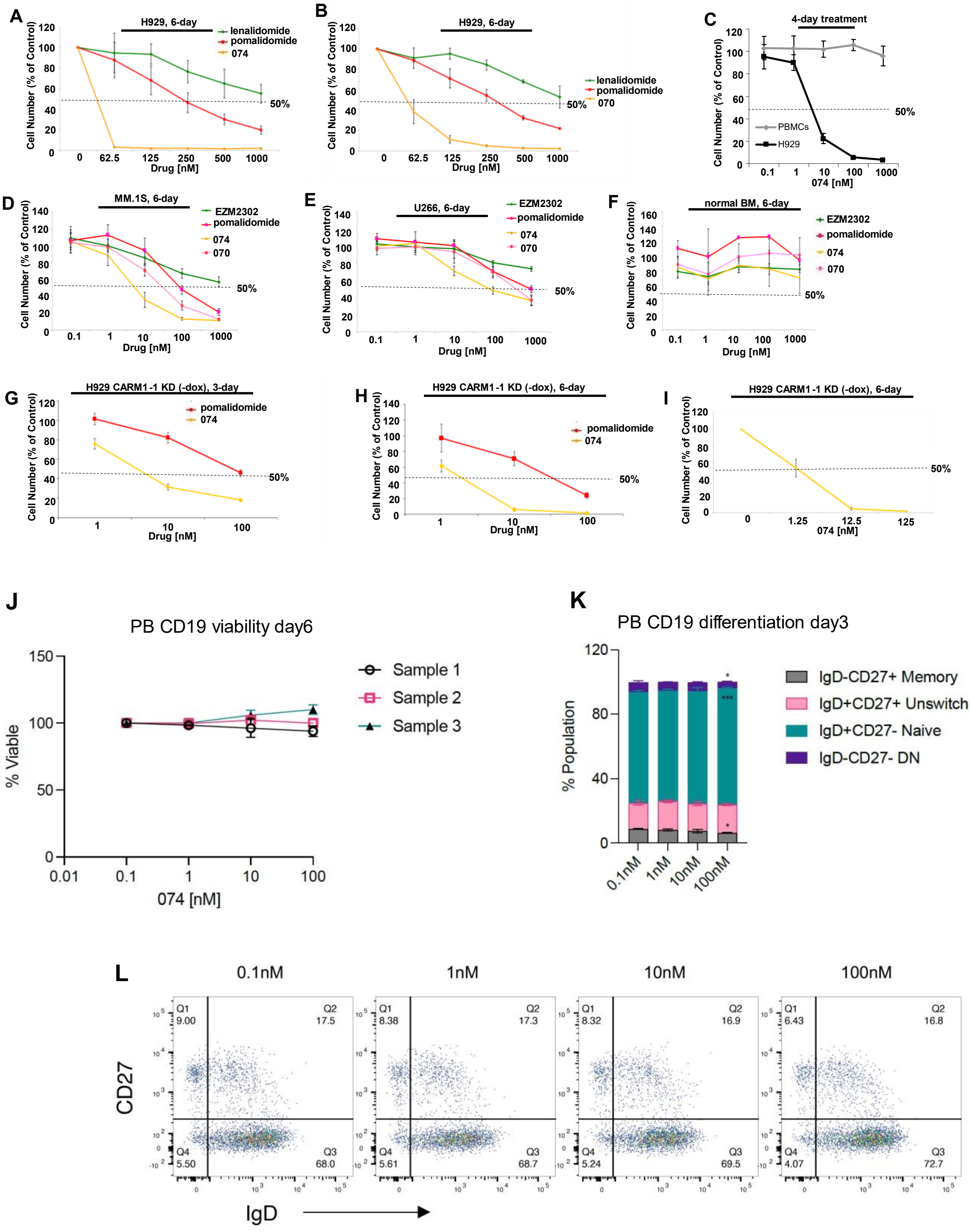
A
H929, 6-day
lenalidomide
pomalidomide
074
Cell Number (% of Control)
50%
Drug [nM]
B
H929, 6-day
lenalidomide
pomalidomide
070
Cell Number (% of Control)
50%
Drug [nM]
C
4-day treatment
PBMCs
H929
Cell Number (% of Control)
50%
074 [nM]
D
MM.1S, 6-day
EZM2302
pomalidomide
074
070
Cell Number (% of Control)
50%
Drug [nM]
E
U266, 6-day
EZM2302
pomalidomide
074
070
Cell Number (% of Control)
50%
Drug [nM]
F
normal BM, 6-day
EZM2302
pomalidomide
074
070
Cell Number (% of Control)
50%
Drug [nM]
G
H929 CARM1-1 KD (-dox), 3-day
pomalidomide
074
Cell Number (% of Control)
50%
Drug [nM]
H
H929 CARM1-1 KD (-dox), 6-day
pomalidomide
074
Cell Number (% of Control)
50%
Drug [nM]
I
H929 CARM1-1 KD (-dox), 6-day
Cell Number (% of Control)
50%
074 [nM]
J
PB CD19 viability day6
Sample 1
Sample 2
Sample 3
% Viable
074 [nM]
K
PB CD19 differentiation day3
IgD-CD27+ Memory
IgD+CD27+ Unswitch
IgD+CD27- Naive
IgD-CD27- DN
% Population
0.1nM
1nM
10nM
100nM
L
0.1nM
1nM
10nM
100nM
Q1 9.00
Q2 17.5
Q4 5.50
Q3 68.0
Q1 8.38
Q2 17.3
Q4 5.61
Q3 68.7
Q1 8.32
Q2 16.9
Q4 5.24
Q3 69.5
Q1 6.43
Q2 16.8
Q4 4.07
Q3 72.7
CD27
IgD

Figure S6. 070 and 074 more potently inhibit the proliferation of MM cell lines than EZM2302 or IMiDs. (A-B) Proliferation studies: H929 treated with lenalidomide, pomalidomide, 074 or 070 for 6 days. (C) Proliferation assay: 4-day treatment of normal PBMCs versus H929 cells with 074. (D) Proliferation studies: MM.1S treated with EZM2302, pomalidomide, 074 or 070 for 6 days. (E) Proliferation studies: U266 treated with EZM2302, pomalidomide, 074 or 070 for 6 days. (F) Proliferation studies: Normal bone marrow treated with EZM2302, pomalidomide, 074 or 070 for 6 days. (G-H) Proliferation studies: 074 versus pomalidomide treatment of dox-inducible H929 CARM1-1 KD cells in the absence of doxycycline for 3 days (G) and 6 days (H). (I) H929 CARM1-1 KD cells treated with 074 in the absence of doxycycline for 6 days. (J) CD19+ B cells enriched from healthy donor peripheral blood were treated with increasing concentrations of 074, and on day 6 cells were stained with DAPI for HTS acquisition on flow cytometer. The data plot shows cell viability (mean and SD) from a minimum of 5 replicates with each concentration normalized to the smallest concentration of 0.1nM. (K) CD19+ cells from one sample were treated in triplicates and subjected to flow cytometry on day 3. The statistical plot shows mean and SD, and comparison from 0.1nM in two-way ANOVA using Dunnett's multiple comparisons test; asterisk shows $p<0.01$. (L) A representative flow cytometry plot showing distribution of naïve (IgD+CD27-), unswitched (IgD+CD27+), memory (IgD-CD27+) and double negative cells on day 3 post-treatment.

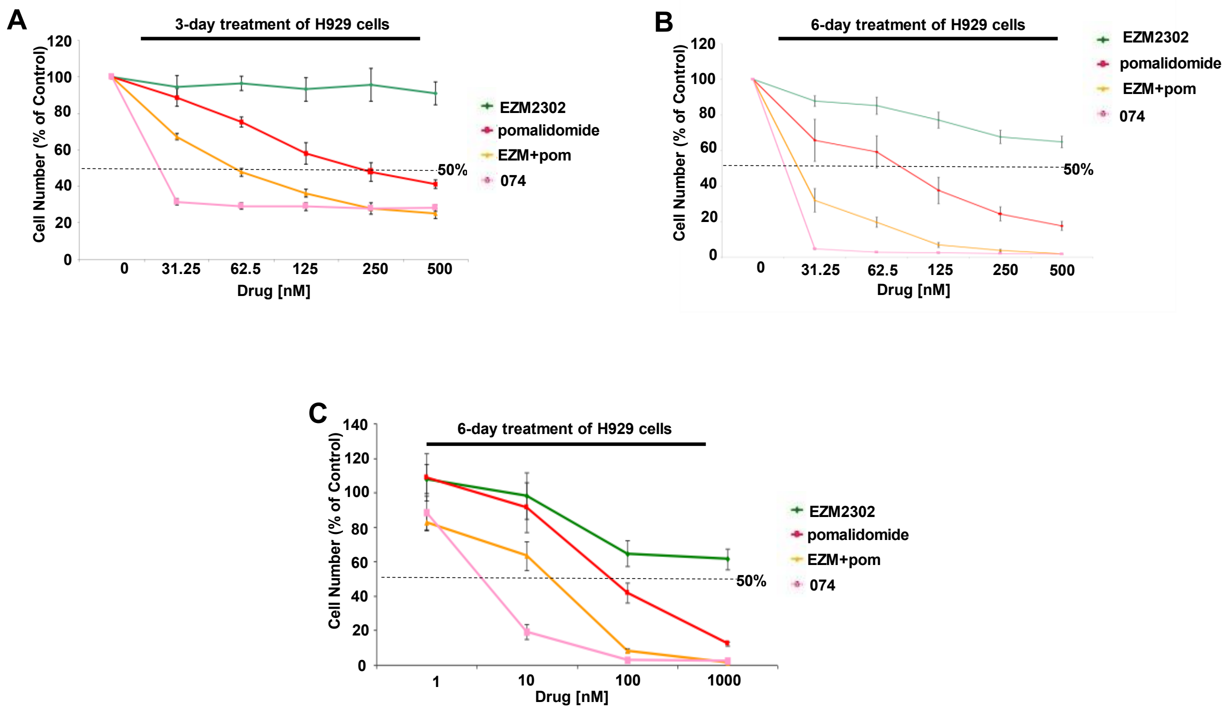

Figure S7. Comparison of the potency of 074 versus the combination of CARM1 inhibition+pomalidomide. (A-B) Proliferation studies: H929 cells treated with EZM2302, pomalidomide, EZM2302+pomalidomide, or 074 for 3 days (A) or 6 days (B) (concentration range 31.25-500 nM). (C) Proliferation studies: H929 cells treated with EZM2302, pomalidomide, EZM2302+pomalidomide, or 074 for 6 days (concentration range 1-1000 nM).

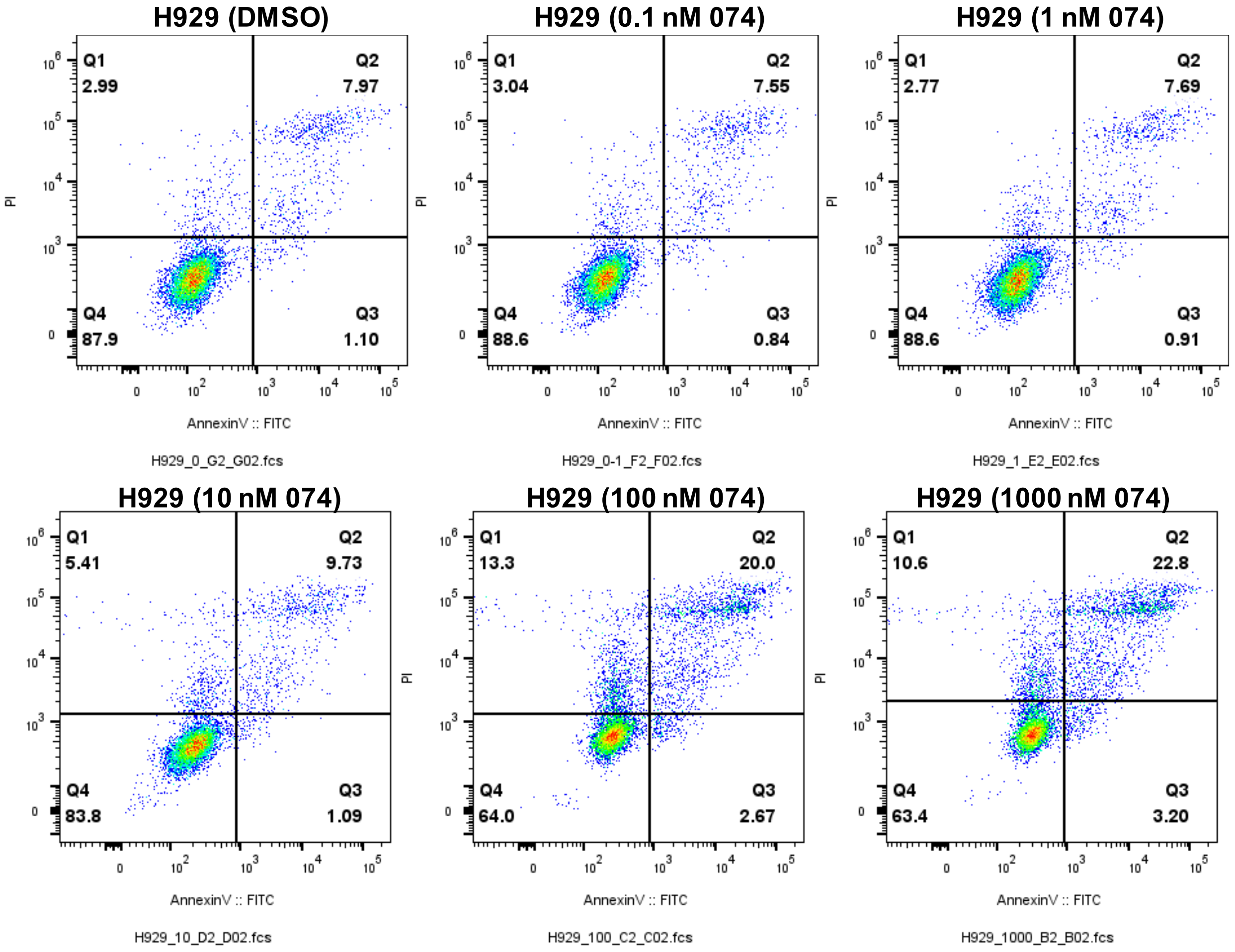

Figure S8. 074 induces late-stage apoptosis and necrosis in a concentration-dependent manner. 4-day 074 treatment of H929 cells followed by Annexin/PI staining as a measure of apoptosis.

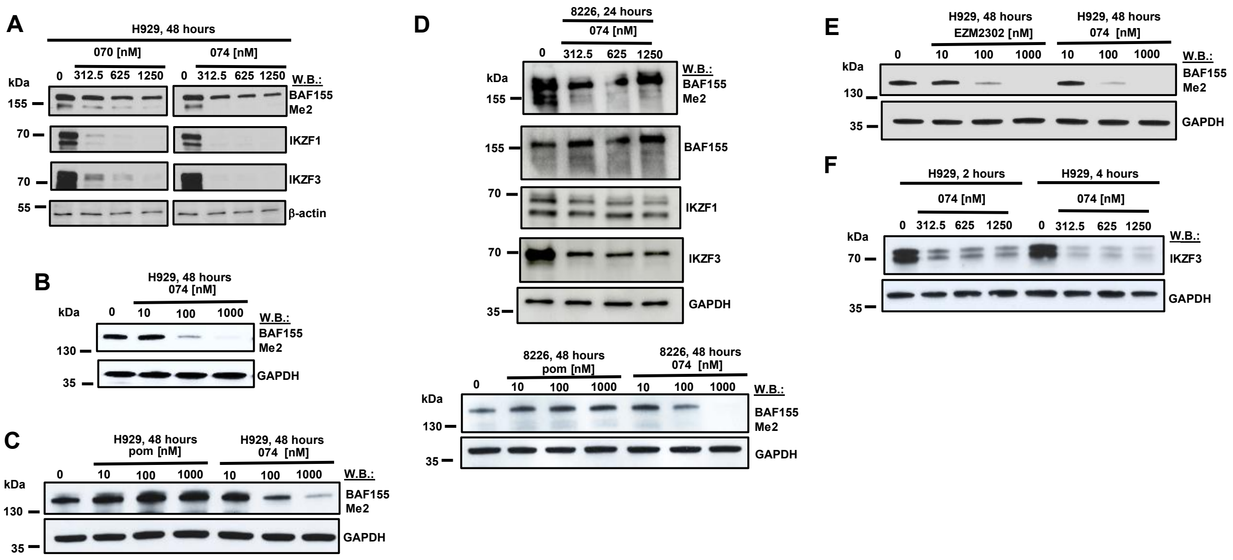

Figure S9 (A-F). Effects of 070 and 074 on BAF155 methylation and expression of IKZF1, IKZF3, MYC and IRF4. (A) Immunoblots: Effects of 070 and 074 on expression of methylated BAF155, IKZF1, and IKZF3 following 48-hour treatment of H929 cells. (B) Immunoblots: Effects of 074 on expression of methylated BAF155 following 48-hour treatment of H929 cells. (C) Immunoblots: Comparison of effects of pomalidomide and 074 on methylation of BAF155 in H929 cells. (D) Effects of 074 on expression of methylated BAF155, IKZF1, and IKZF3 following 24 hours of treatment of 8226 cells (lower panel) and effects of pomalidomide versus 074 on methylated BAF155 following 48 hours of treatment of 8226 cells (right panel). (E) Comparison of effects of EZM2302 and 074 on methylation of BAF155 in H929 cells following 48 hours of treatment. (F) Immunoblots: Effects of 074 on IKZF3 expression following 2-hour and 4-hour treatment of H929 cells.

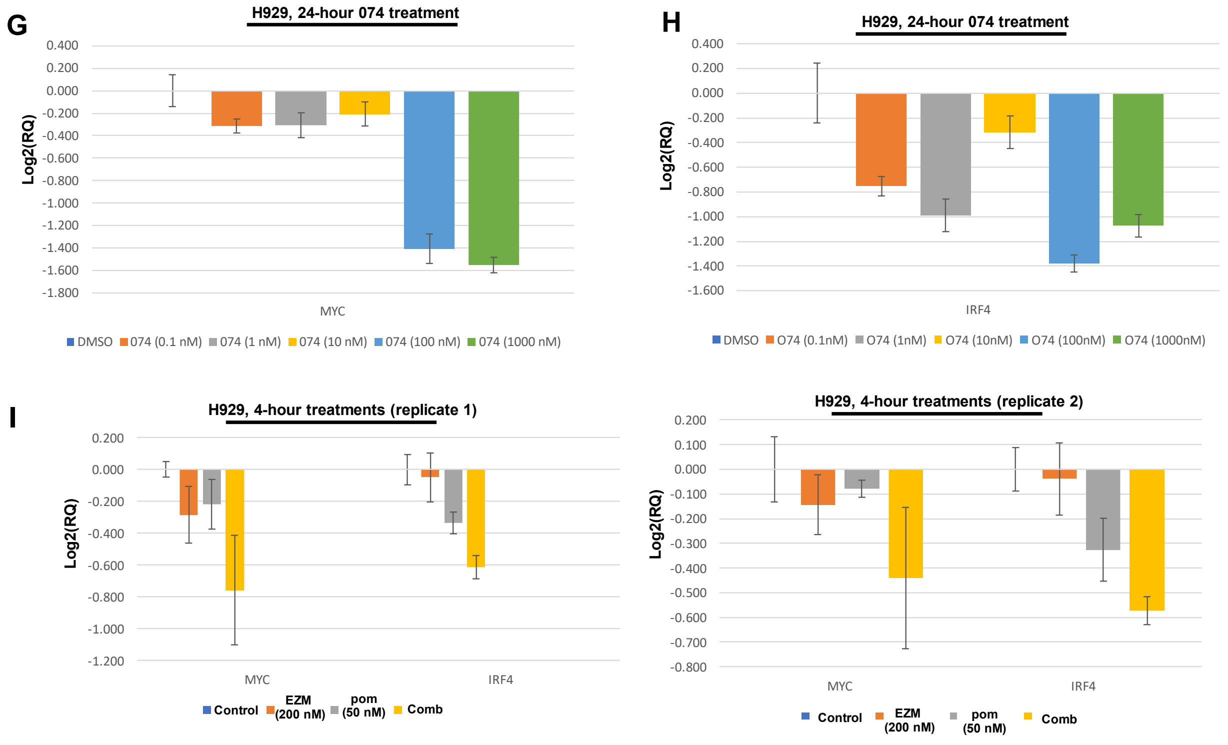

Figure S9 (G-I). Effects of CARM1 targeting+IMiD treatment or 074 on IKZF3, MYC and IRF4 in MM cells. (G) qPCR investigation of effects of 074 on transcription of MYC. (H) qPCR investigation of effects of 074 on transcription of IRF4. (I) qPCR investigation of effects of EZM2302 (200 nM), pomalidomide (50 nM), or the combination following a 4-hour treatment on MYC and IRF4 transcription in H929 cells.

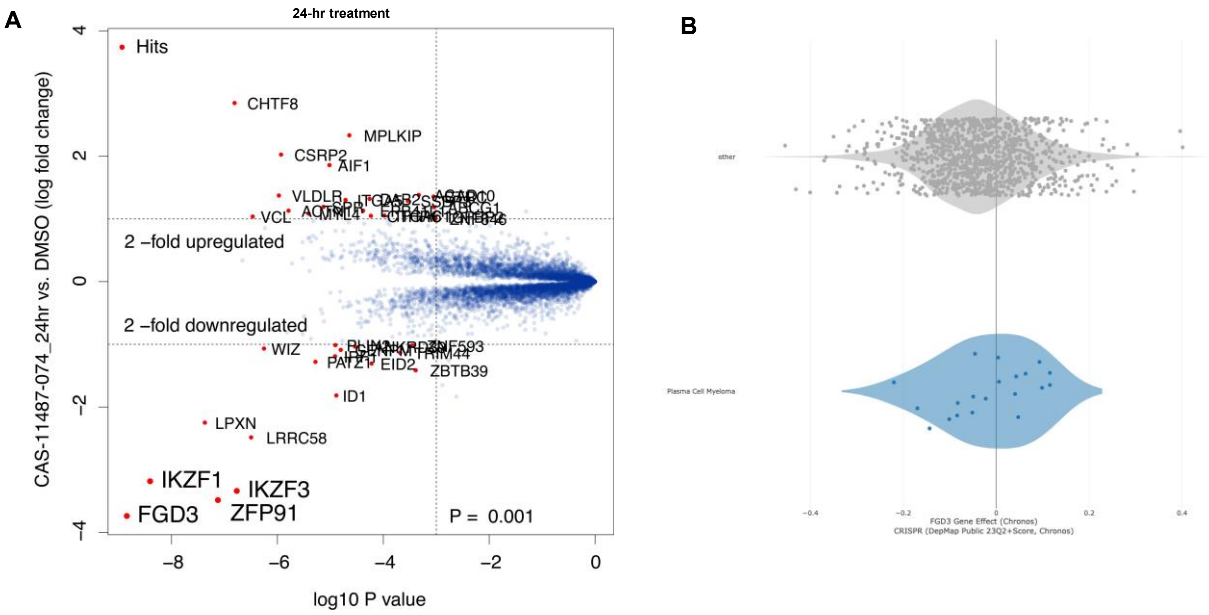

Figure S10. Proteomics identification of protein targets of 074. (A) Proteomics analysis for 074. 24-hour time point. (B) Gene effect of FGD3 targeting in 20 myeloma cell lines versus various other cancer cell lines from different lineages tested within this CRISPR screen. Data were retrieved from https://depmap.org/.

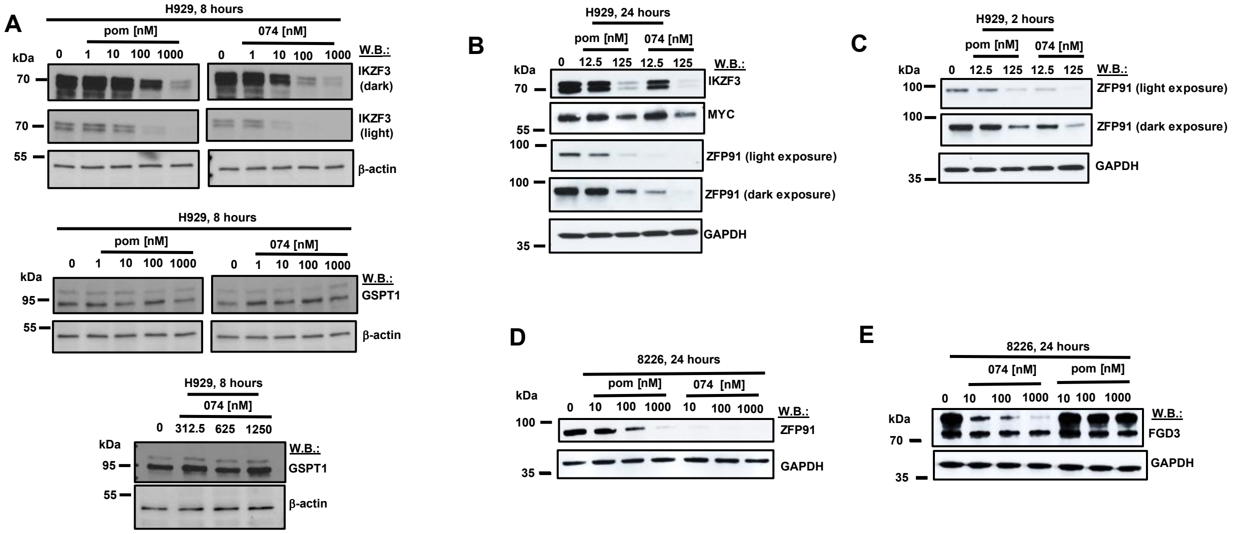

Figure S11. Comparison of effects of pomalidomide and 074 on IKZF1, IKZF3, GSTP1, and ZFP91 protein expression in H929 cells. (A) Effects of pomalidomide or 074 on expression of IKZF3 and GSPT1 following 8-hour treatment of H929 cells. (B) Effects of pomalidomide or 074 on expression of IKZF3, MYC, and ZFP91 following a 24-hour treatment of H929 cells. (C) Effects of pomalidomide or 074 on expression of ZFP91 following 2-hour treatment of H929 cells. (D-E) Effects of pomalidomide or 074 on expression of ZFP91 (D) or FGD3 (E) following a 24-hour treatment of 8226 cells.

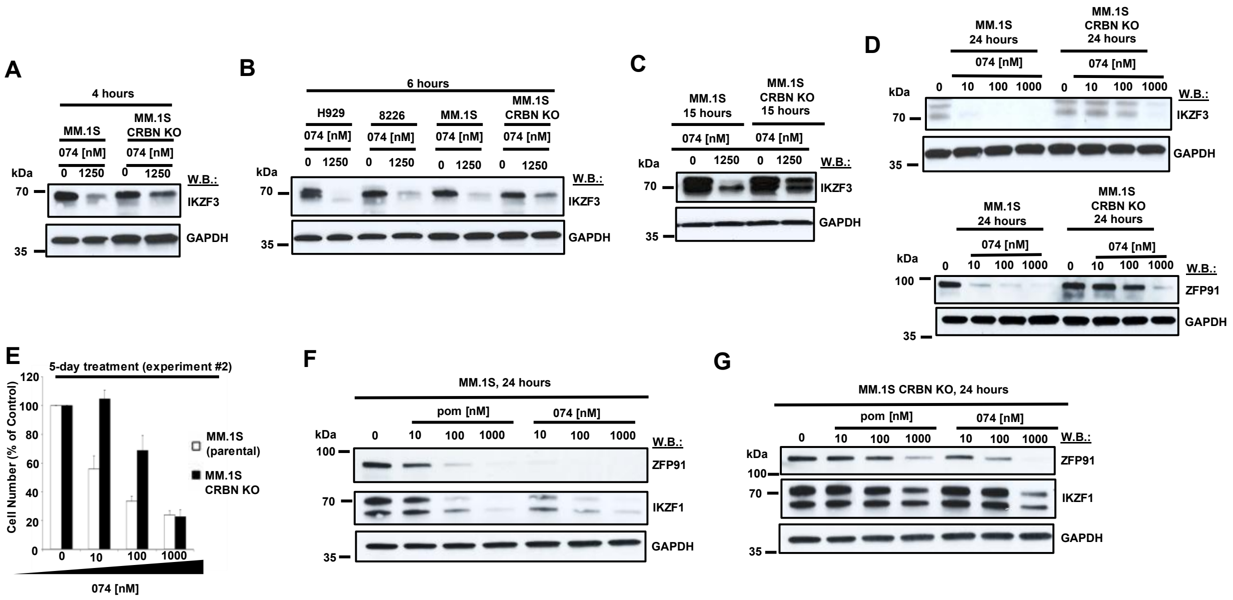

Figure S12. Effects of 074 treatment of MM.1S and MM.1S CRBN KO on IKZF3, CARM1, and ZFP91 protein levels. (A-B) Effects of 074 on IKZF3 protein levels in MM.1S and MM.1S CRBN KO cells following 4 hours (A) and 6 hours (B). In (B), H929 and 8226 cells are included as controls for comparison. (C) Effects of 074 on IKZF3 protein levels in MM.1S and MM.1S CRBN KO cells following 15 hours of treatment. (D) Effects of 074 on IKZF3 and ZFP91 protein levels in MM.1S and MM.1S CRBN KO cells following 24 hours of treatment (E) Proliferation study: 5-day 074 treatment of parental MM.1S cells and MM.1S CRBN KO cells. . (F) Immunoblots: Comparison of effects of pomalidomide and 074 on ZFP91 and IKZF1 protein expression following 24 hours of treatment of MM.1S cells. (G) Immunoblots: Comparison of effects of pomalidomide and 074 on ZFP91 and IKZF1 protein expression following 24 hours of treatment of MM.1S CRBN KO cells.

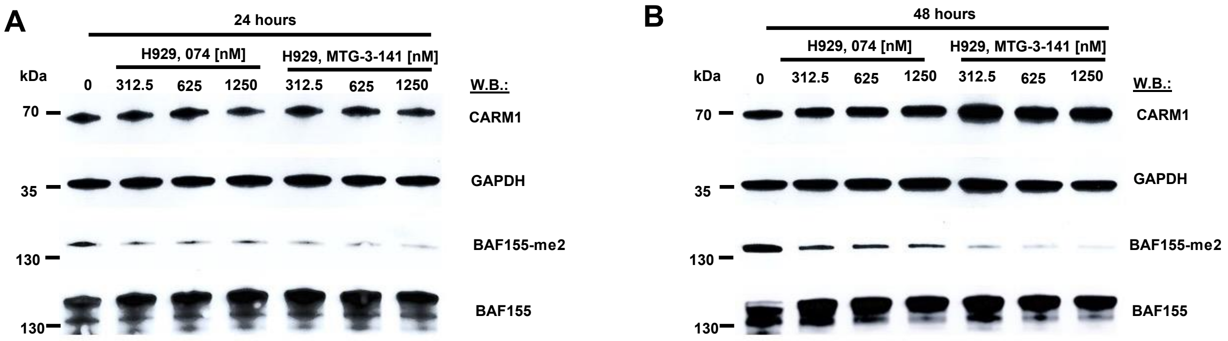

Figure S13. Effects of 074 or MTG-3-141 treatment of H929 cells on CARM1, meBAF155 and total BAF155. (A-B) Immunoblots: Effects of 24- and 48-hour 074 or MTG-3-141 treatment of H929 on levels of CARM1, methylated BAF155 and total BAF155.

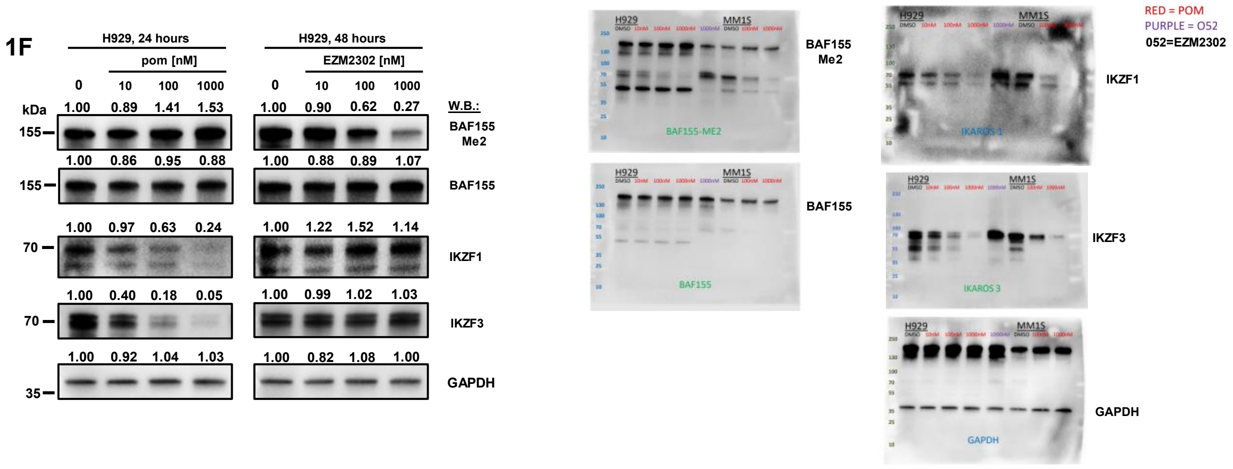

Figure S14 (part 1). Uncut gels/densitometry for Figure 1F (left panel).

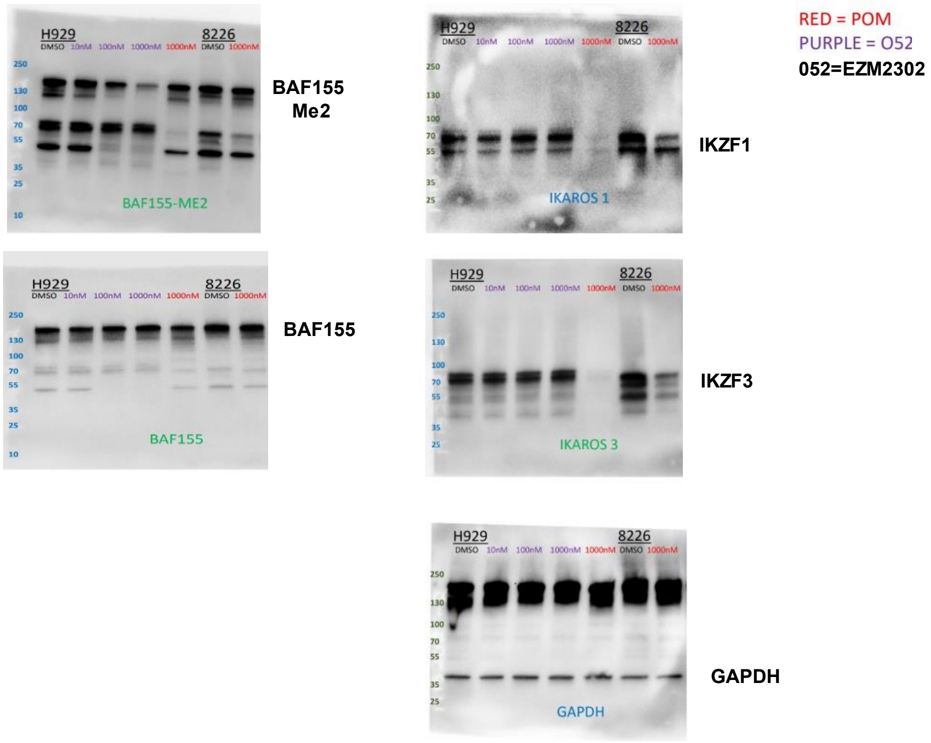

Figure S14 (part 2). Uncut gels/densitometry for Figure 1F (right panel).

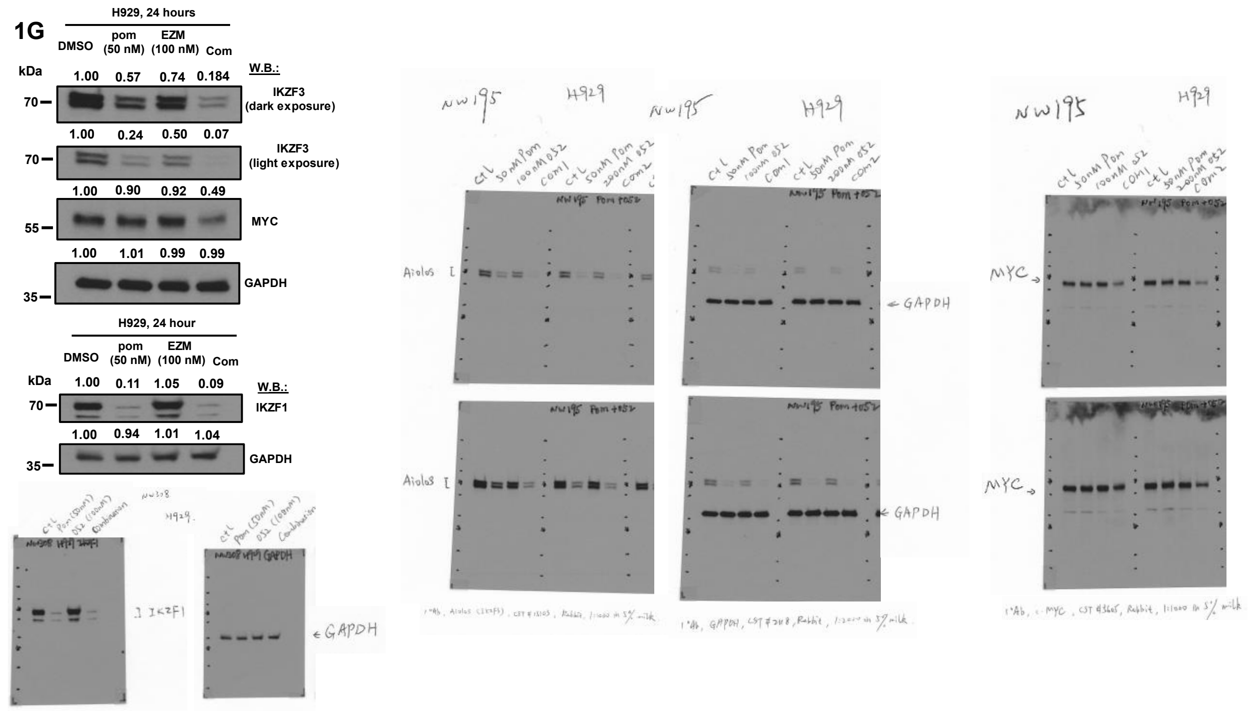

Figure S15. Uncut gels/densitometry for Figure 1G.

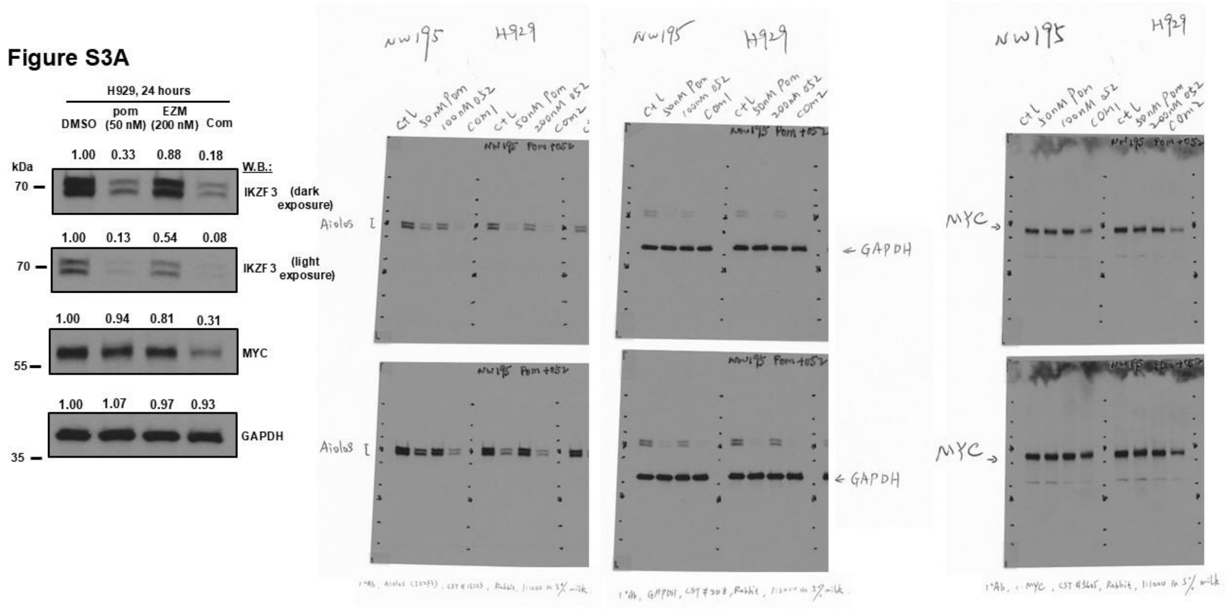

Figure S16 (part 1). Uncut gels/densitometry for Figure S3.

Figure S3B

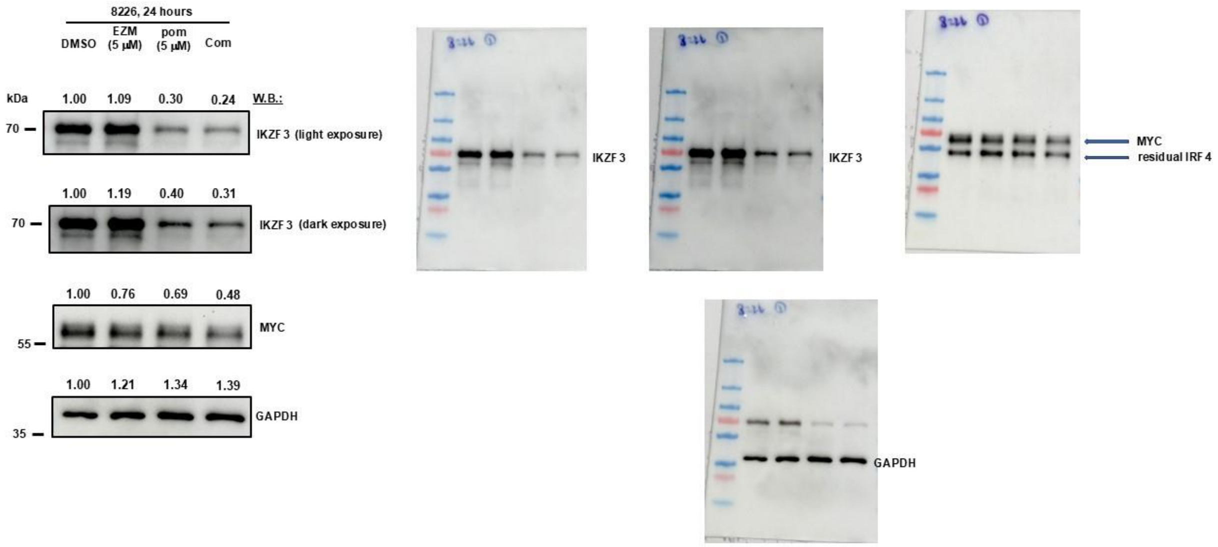

Figure S16 (part 2). Uncut gels/densitometry for Figure S3.

Figure S3C

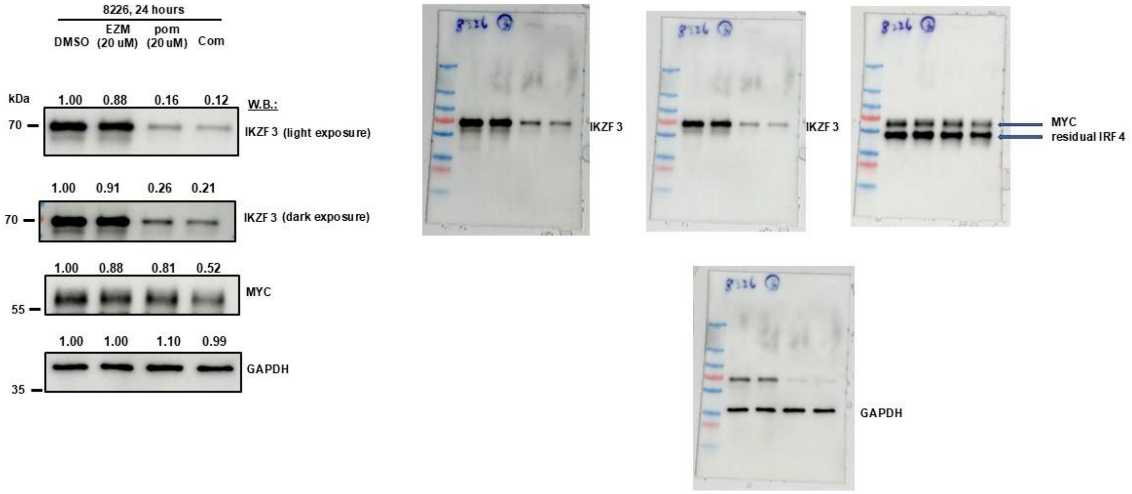

Figure S16 (part 3). Uncut gels/densitometry for Figure S3.

Figure S4C

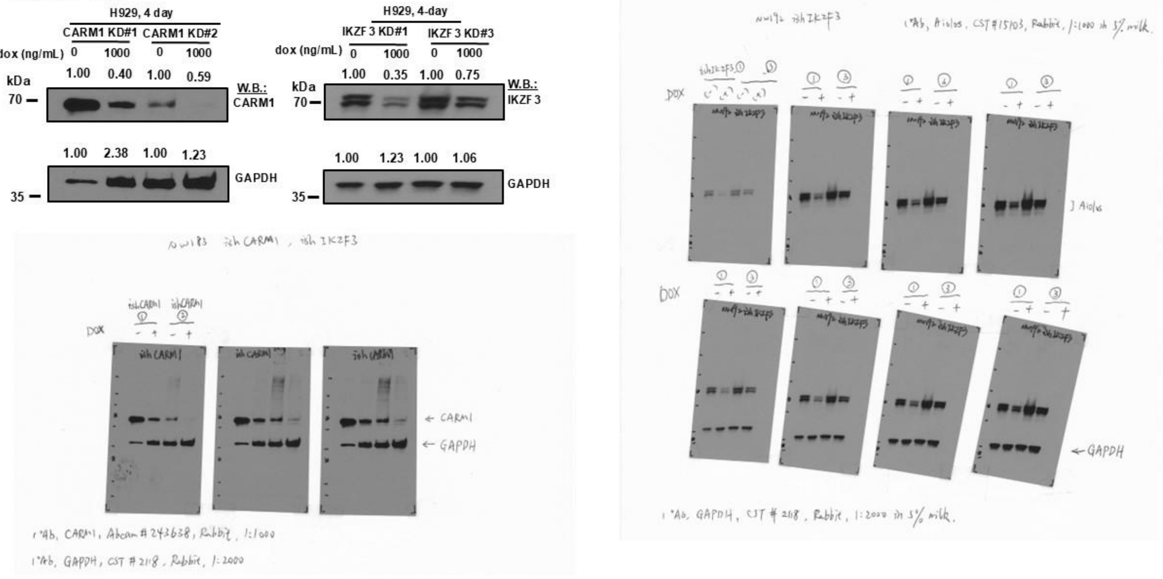

Figure S17. Uncut gels/densitometry for Figure S4C.

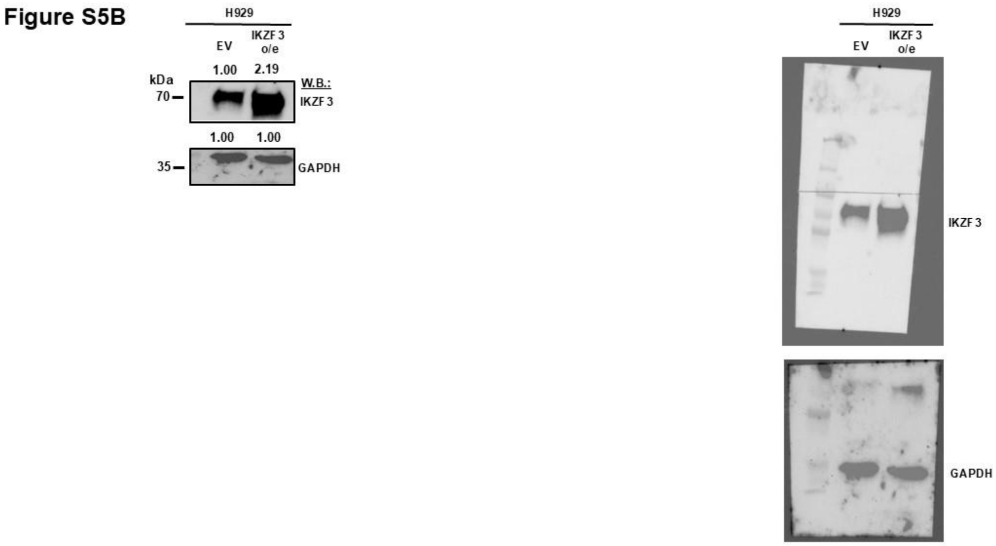

Figure S18. Uncut gels/densitometry for Figure S5B.

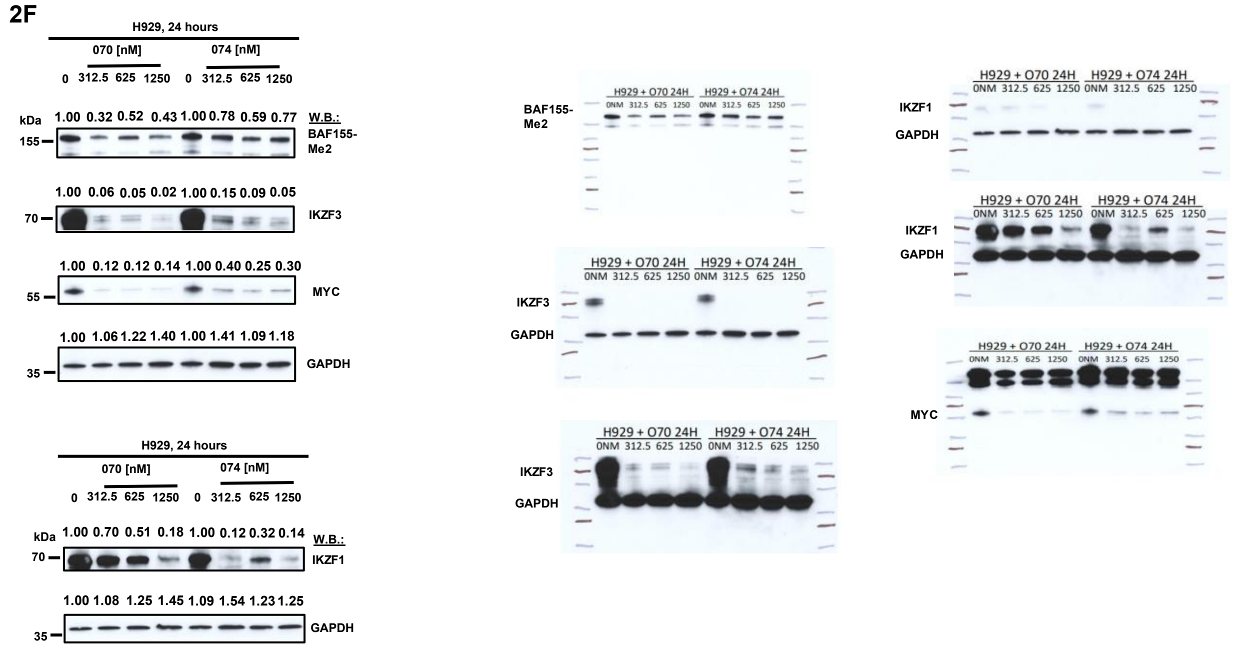

Figure S19 (part 1). Uncut gels/densitometry for Figure 2F.

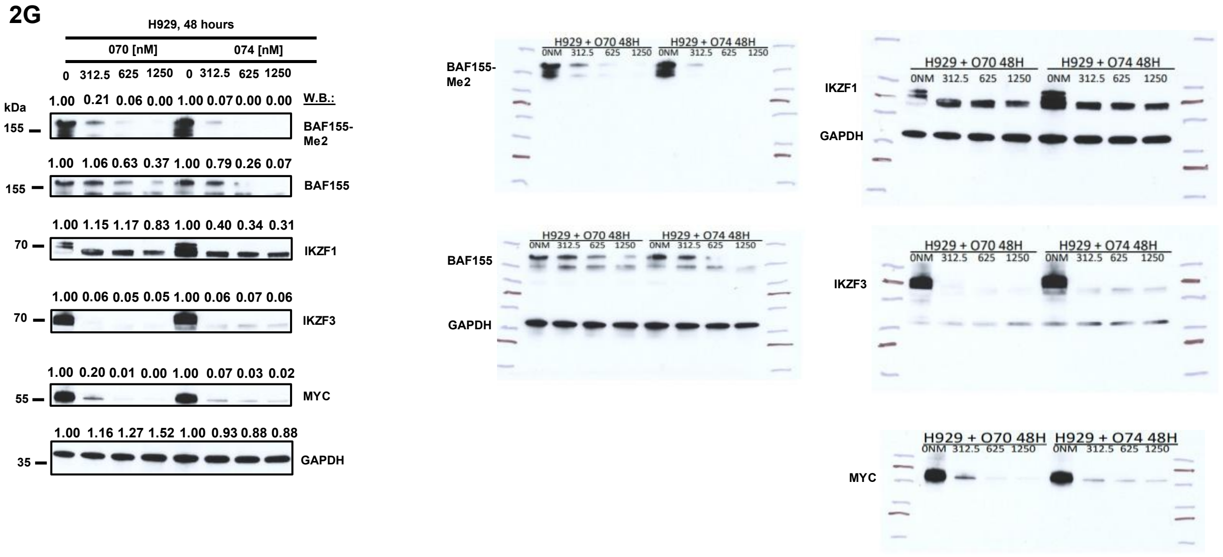

Figure S19 (part 2). Uncut gels/densitometry for Figure 2G.

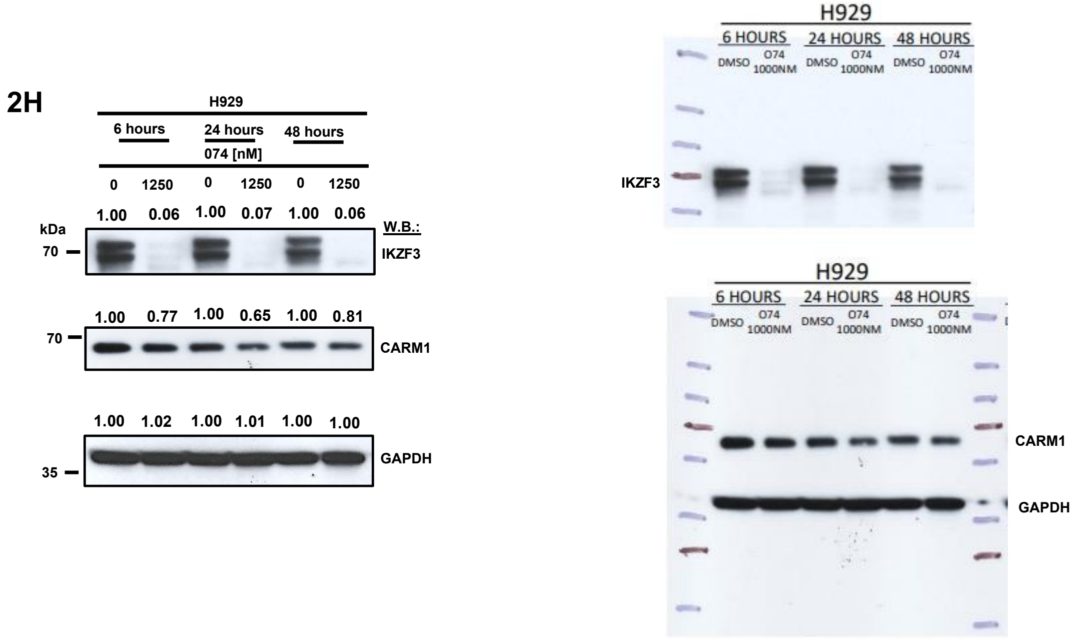

Figure S19 (part 3). Uncut gels/densitometry for Figure 2H.

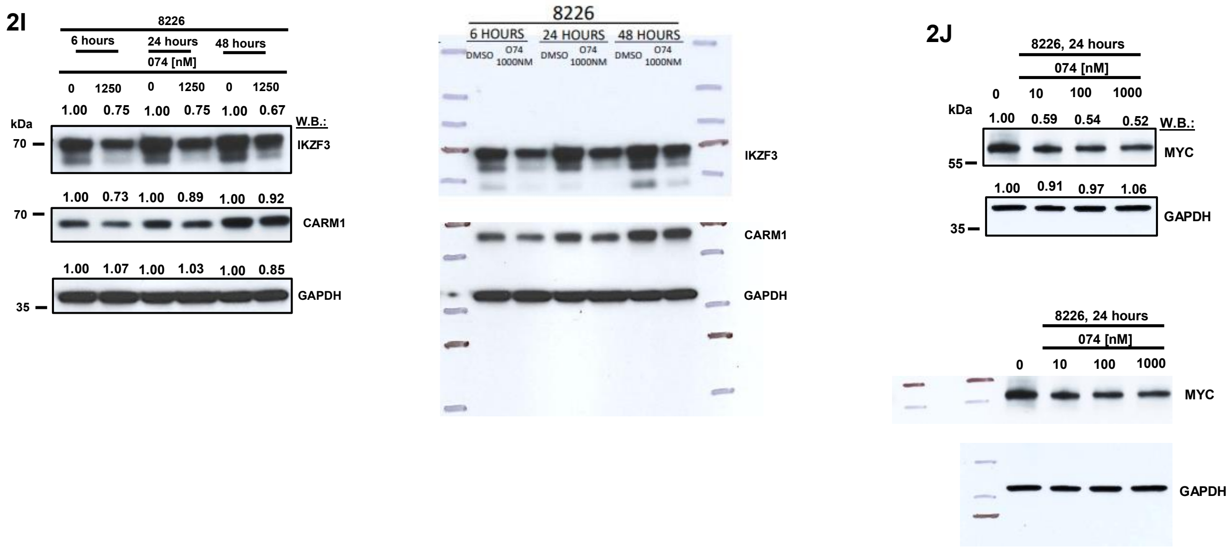

Figure S19 (part 4). Uncut gels/densitometry for Figure 2I and J.

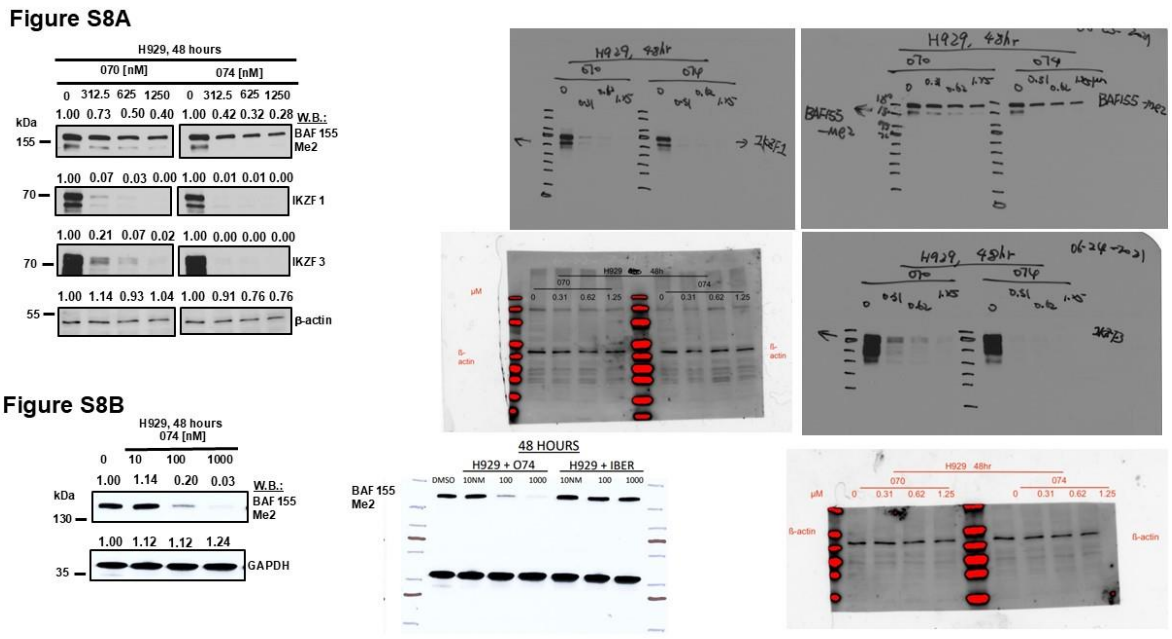

Figure S20 (part 1). Uncut gels/densitometry for Figure S8A and B.

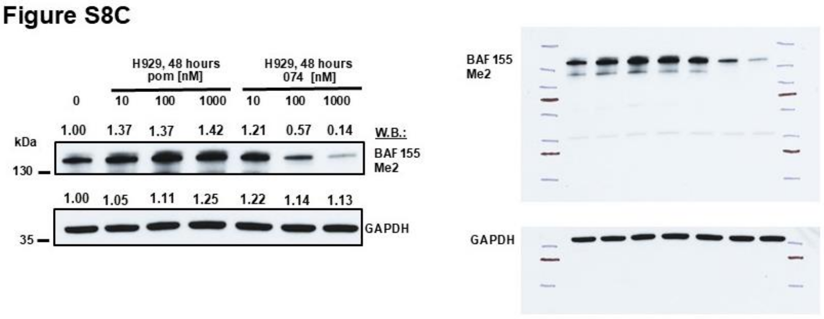

Figure S20 (part 2). Uncut gels/densitometry for Figure S8C.

Figure S8D

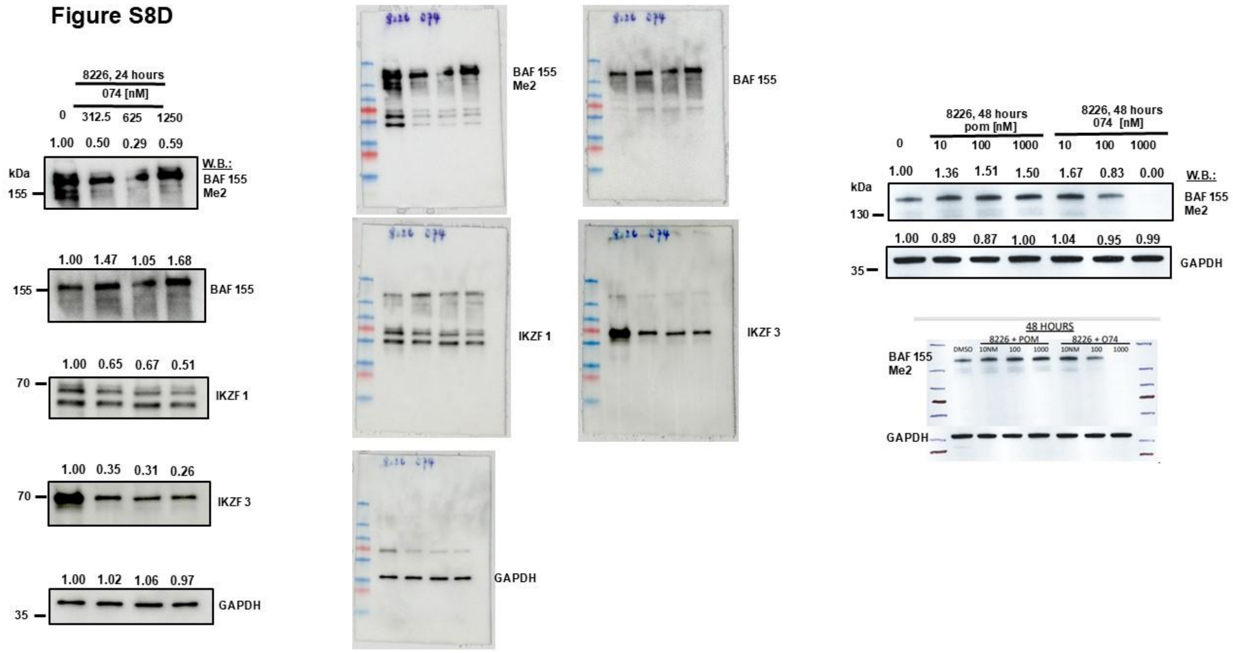

Figure S20 (part 3). Uncut gels/densitometry for Figure S8D.

Figure S8E

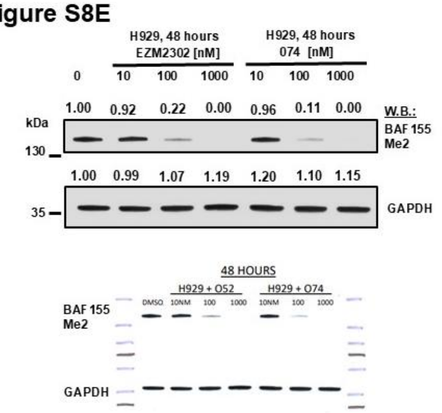

Figure S8F

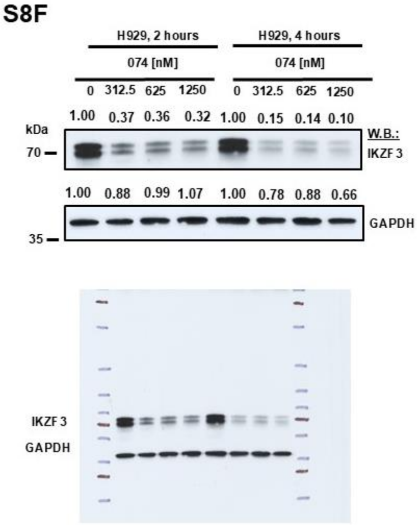

Figure S20 (part 4). Uncut gels/densitometry for Figure S8E and F.

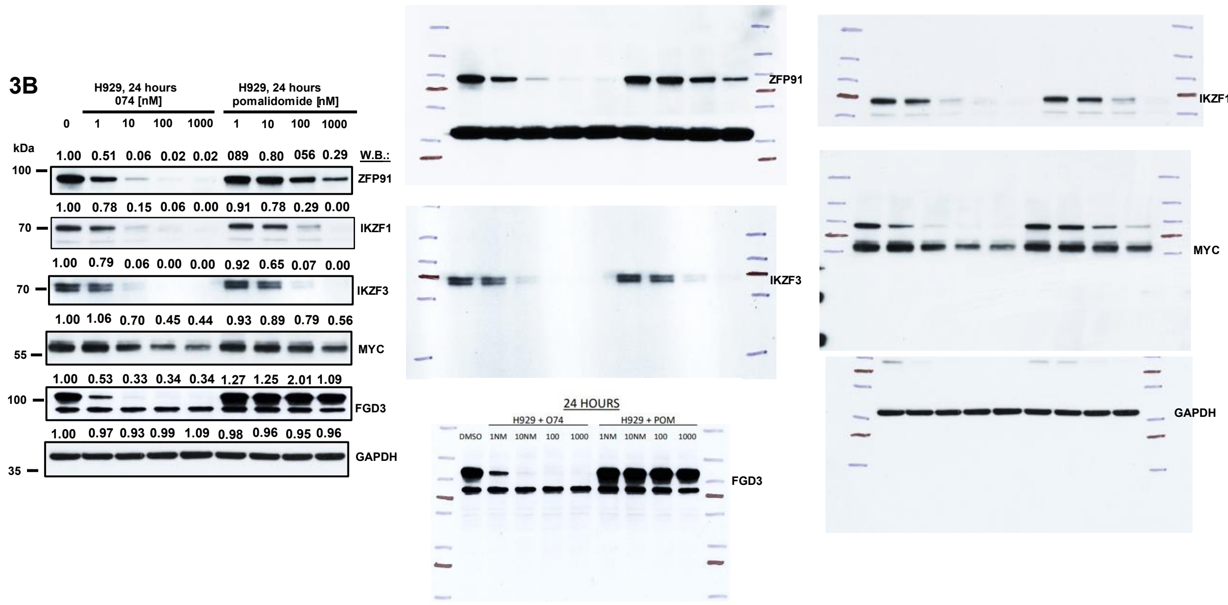

Figure S21 (part 1). Uncut gels/densitometry for Figure 3B.

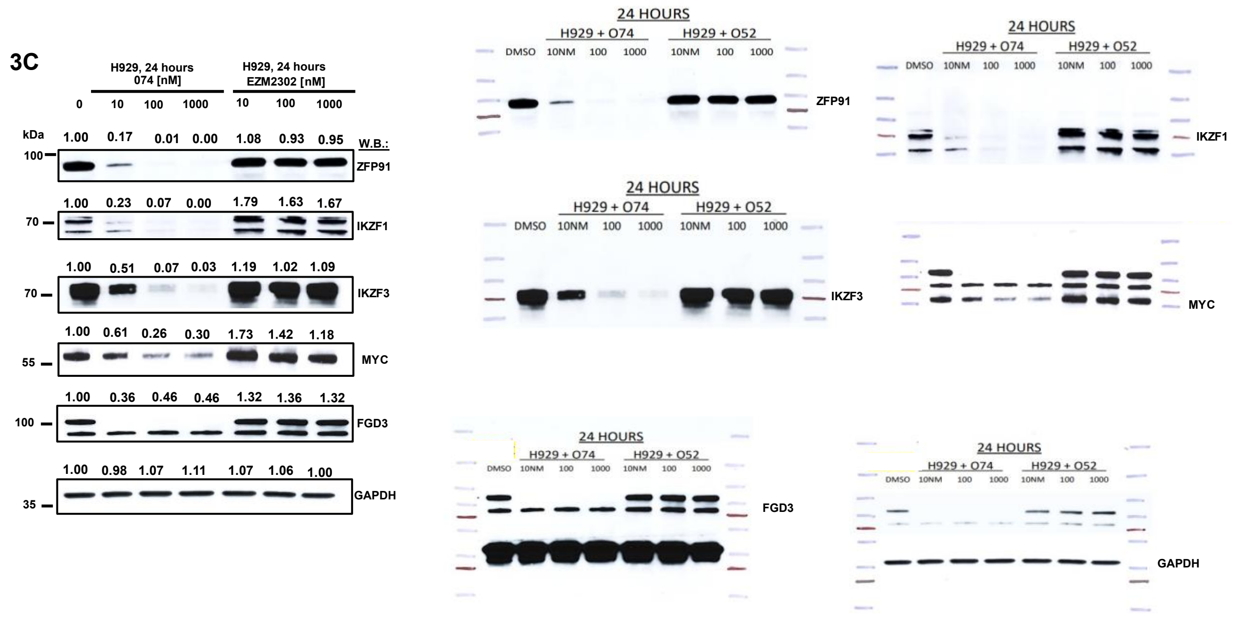

Figure S21 (part 2). Uncut gels/densitometry for Figure 3C.

Figure S10A

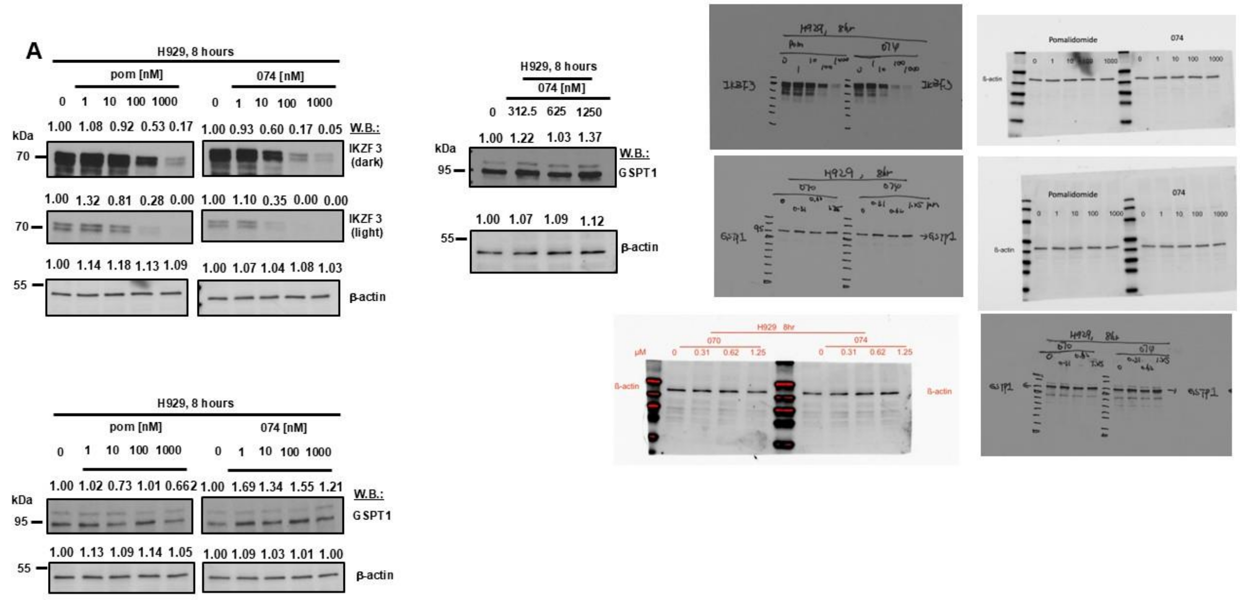

Figure S22 (part 1). Uncut gels/densitometry for Figure S10A.

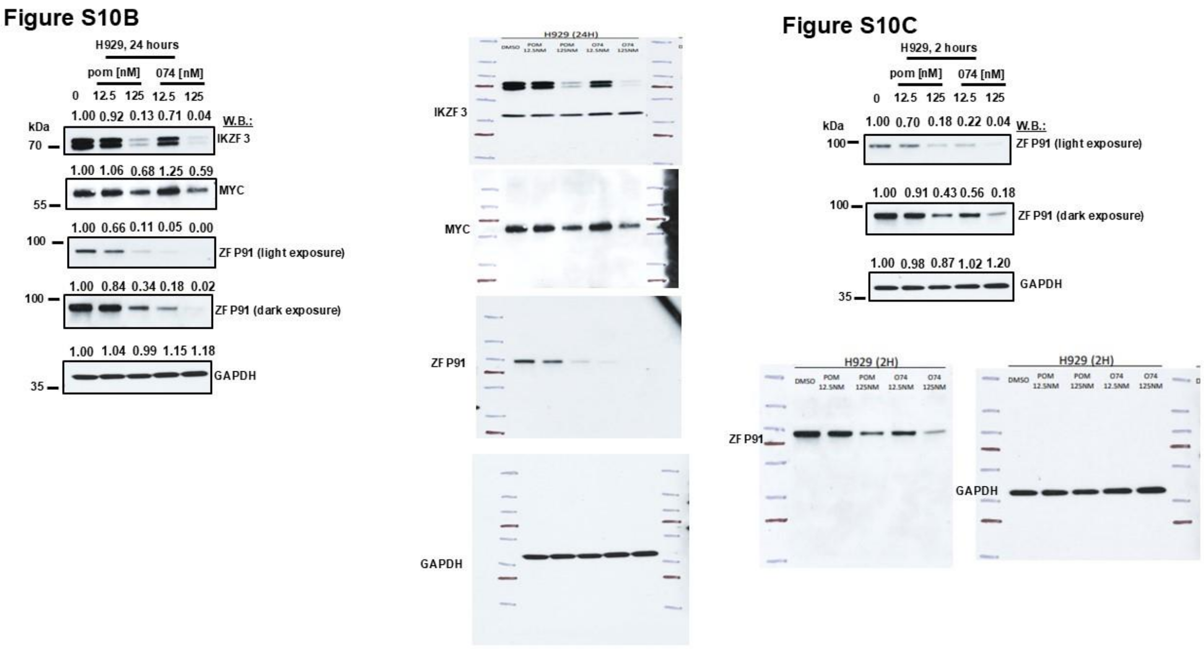

Figure S22 (part 2). Uncut gels/densitometry for Figure S10B and C.

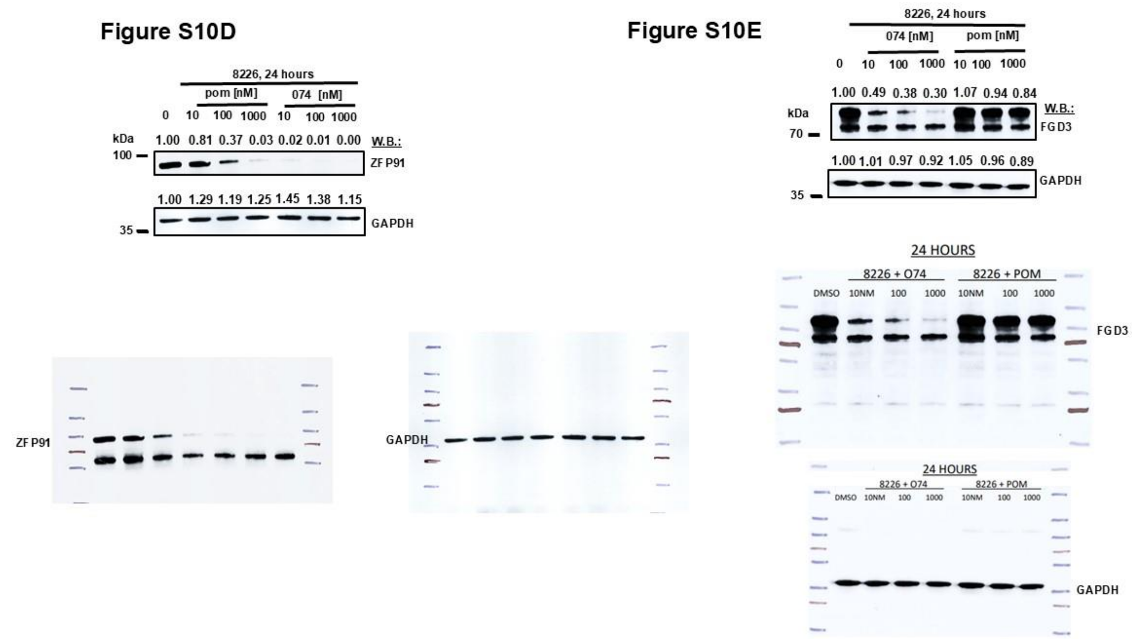

Figure S22 (part 3). Uncut gels/densitometry for Figure S10D and E.

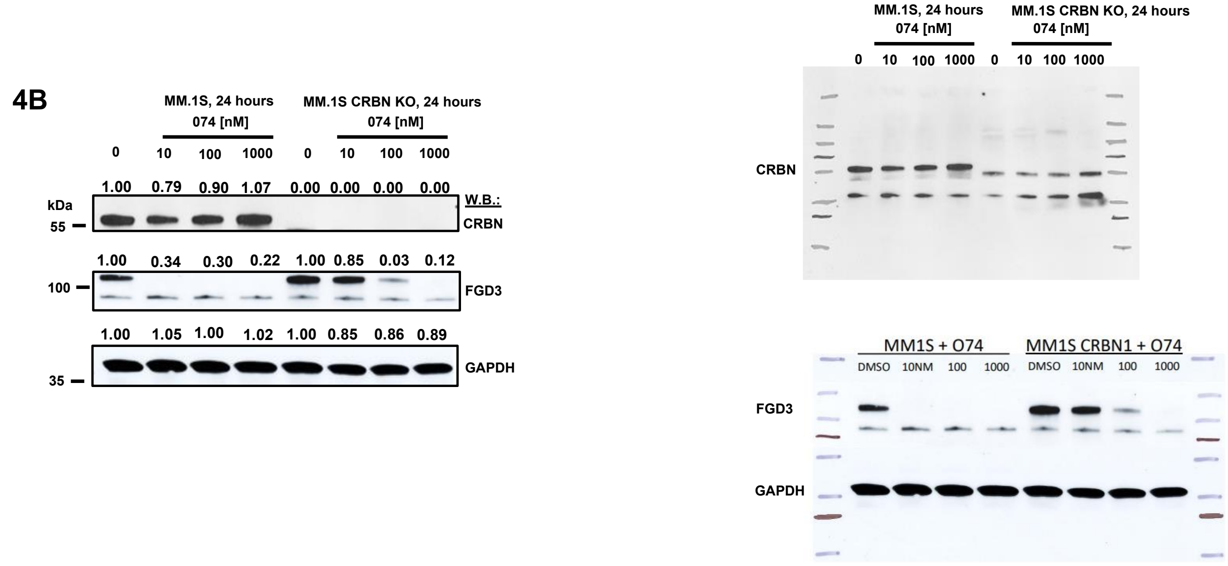

Figure S23 (part 1). Uncut gels/densitometry for Figure 4B.

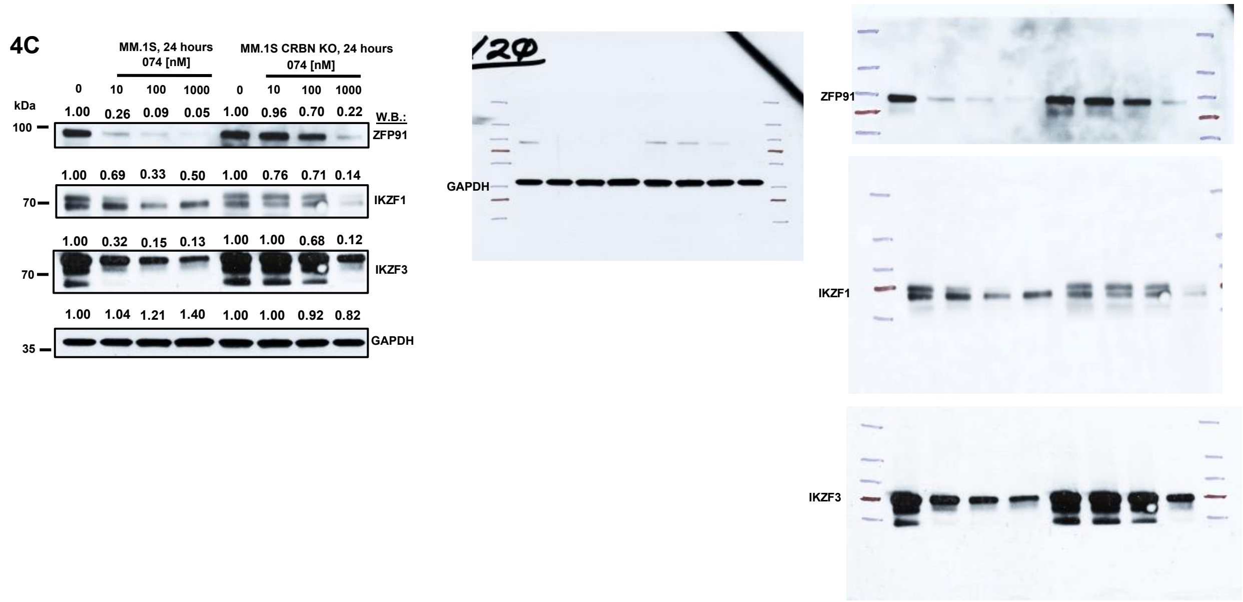

Figure S23 (part 2). Uncut gels/densitometry for Figure 4C.

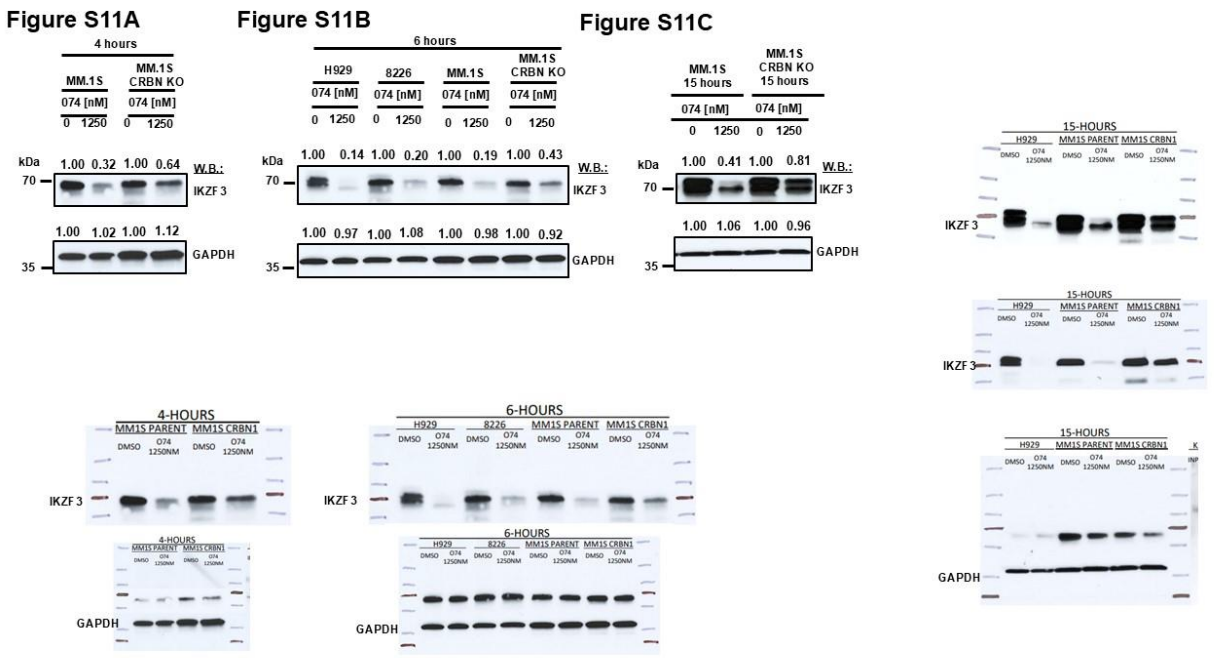

Figure S24 (part 1). Uncut gels/densitometry for Figure S11A-C.

Figure S11D

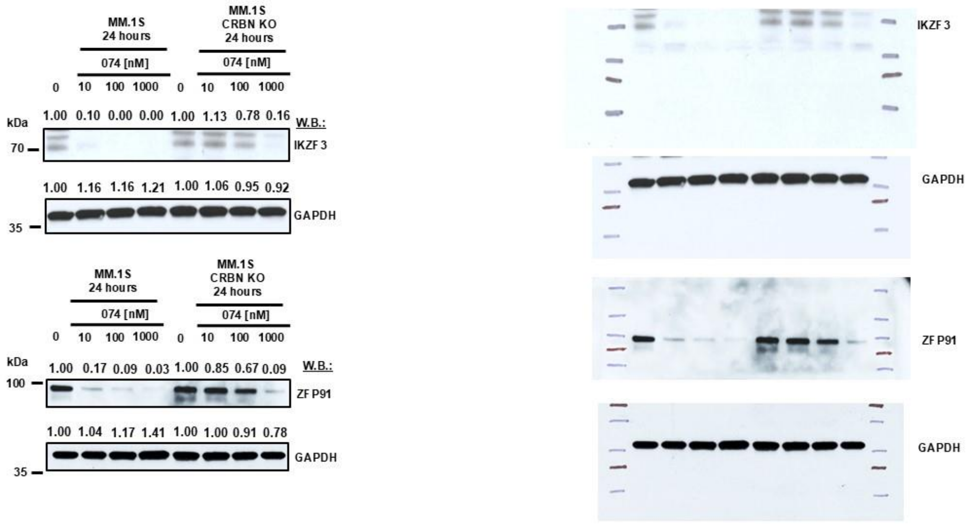

Figure S24 (part 2). Uncut gels/densitometry for Figure S11D.

Figure S11F

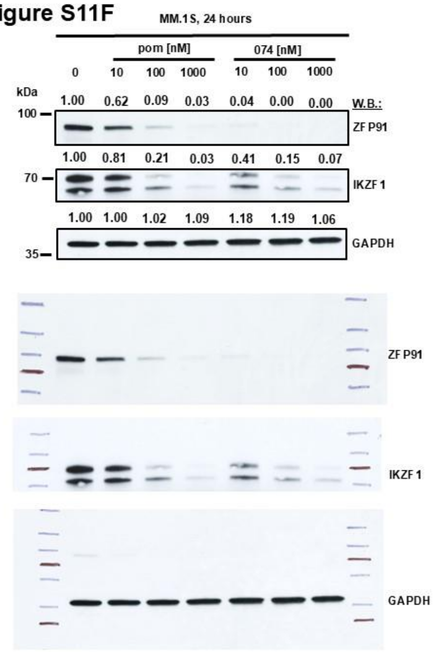

Figure S11G

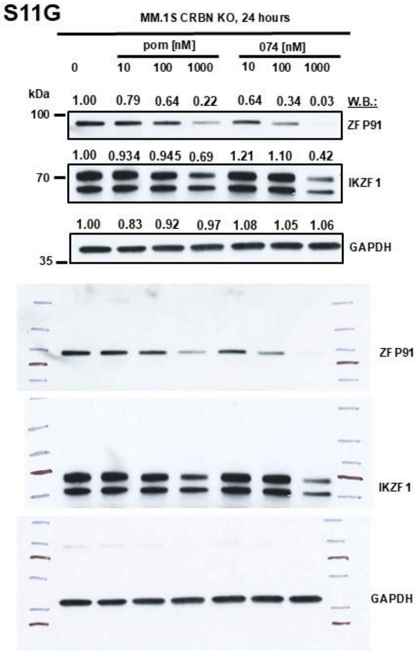

Figure S24 (part 3). Uncut gels/densitometry for Figure S11F and G.

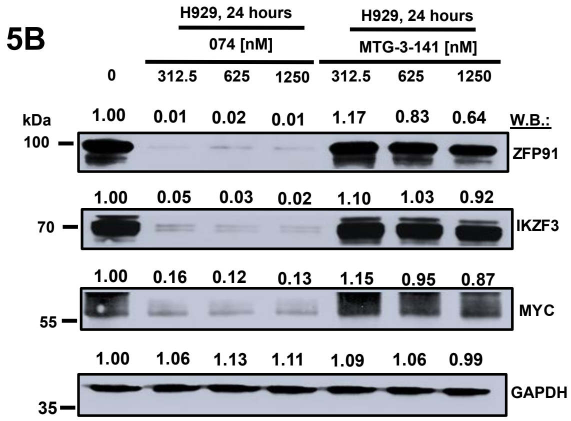

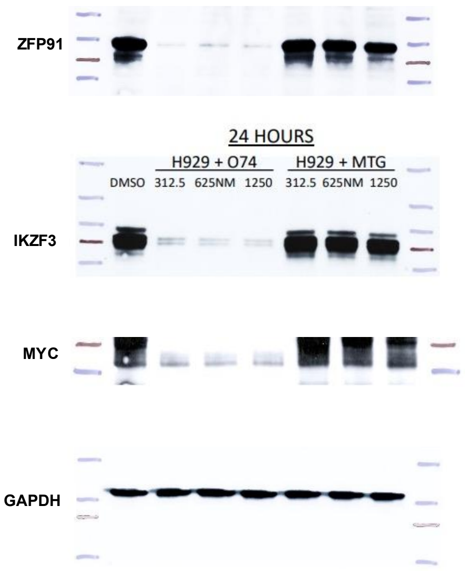

Figure S25 (part 1). Uncut gels/densitometry for Figure 5B.

5C

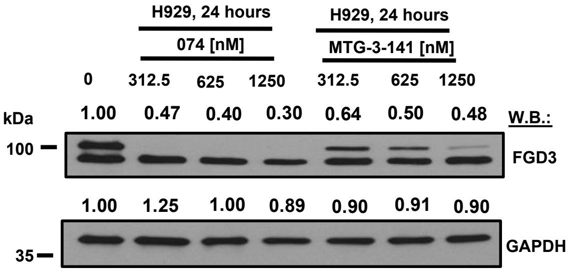

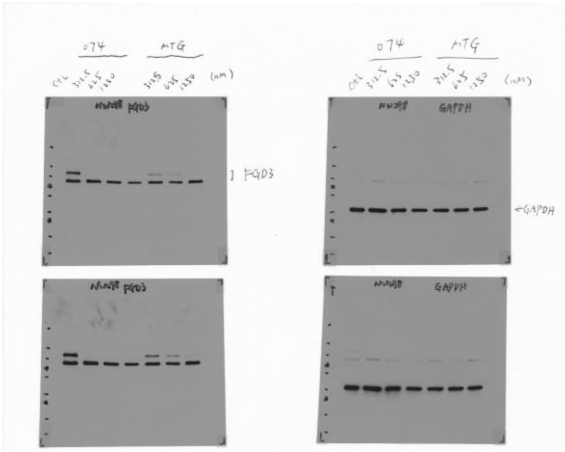

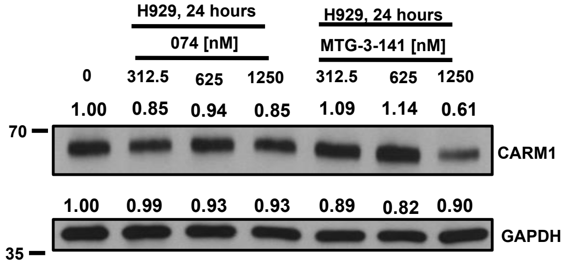

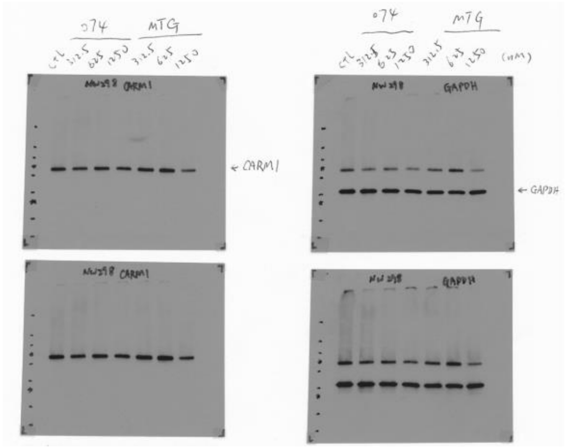

Figure S25 (part 2). Uncut gels/densitometry for Figure 5C.

Figure S12A

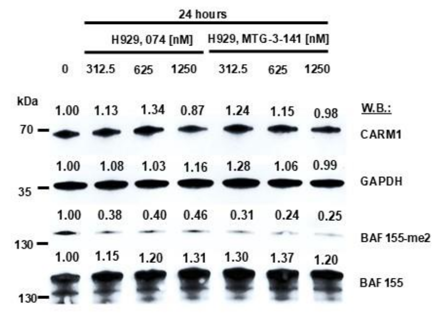

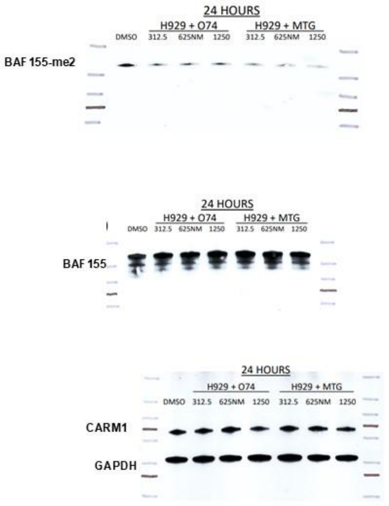

Figure S26 (part 1). Uncut gels/densitometry for Figure S12A.

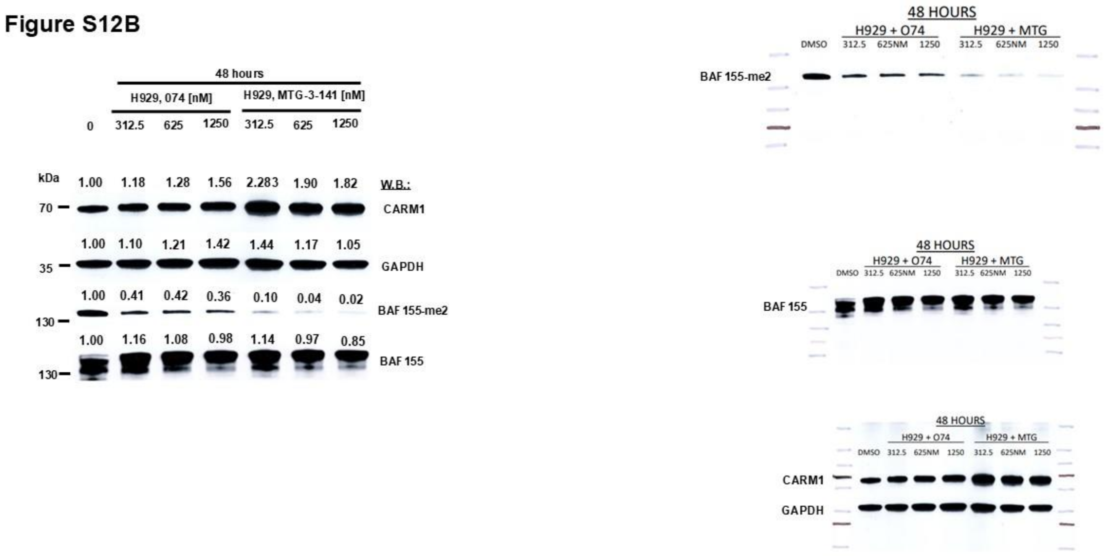

Figure S26 (part 2). Uncut gels/densitometry for Figure S12B.

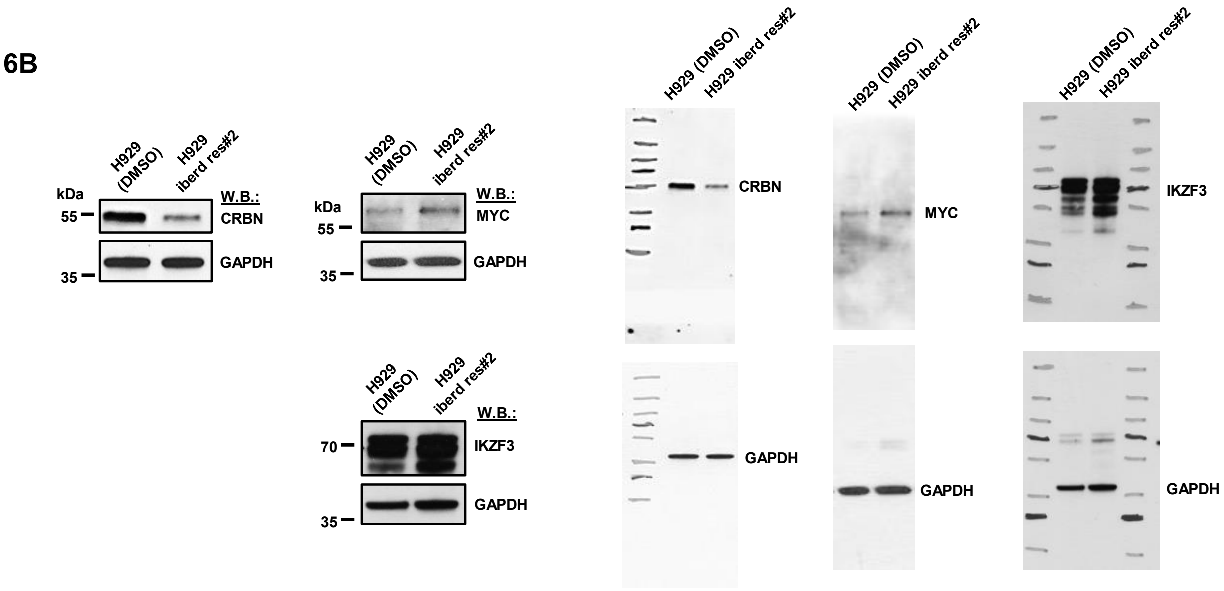

Figure S27. Uncut gels/densitometry for Figure 6B.

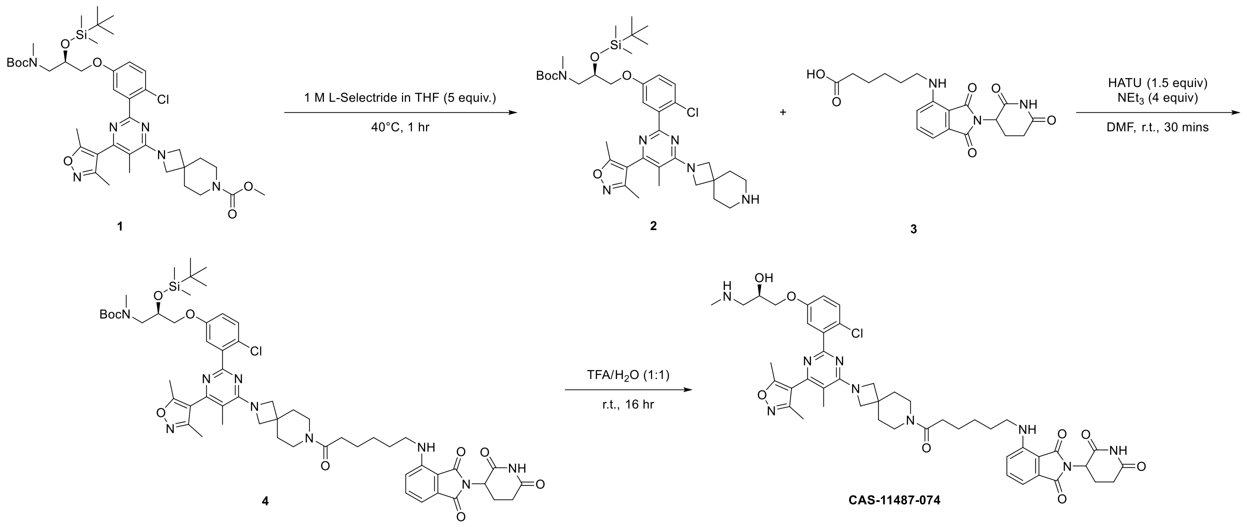

Figure S28. Synthetic Procedure and Characterization of CAS-11487-074.

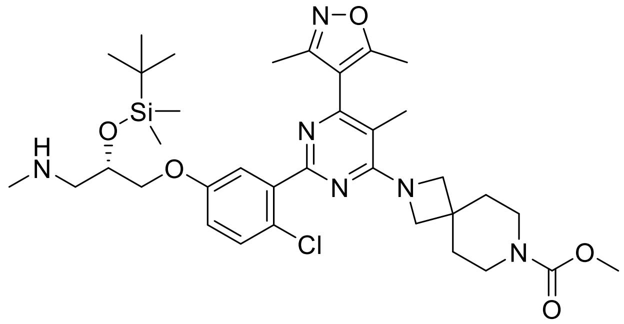

2

Figure S29. Intermediate 2. A borosilicate glass vial equipped with a magnetic stir bar was charged with a solution of 1 M L-Selectride in THF (2.28 mL, 2.28 mmol) and compound **1** (364 mg, 0.455 mmol). The solution was stirred at 40 °C for 1 hour. Upon completion, the reaction mixture was cooled to room temperature and concentrated under reduced pressure. The crude reaction product was then purified by flash chromatography (gradient from 20:80 to 90:10 methanol with 5% triethylamine: ethyl acetate) to afford the product as a clear liquid (289 mg, 86% yield). LCMS (ESI) $[M+H]^+$ calculated for $C_{38}H_{58}ClN_6O_5Si^+$m/z 742.45, found m/z 742.81.

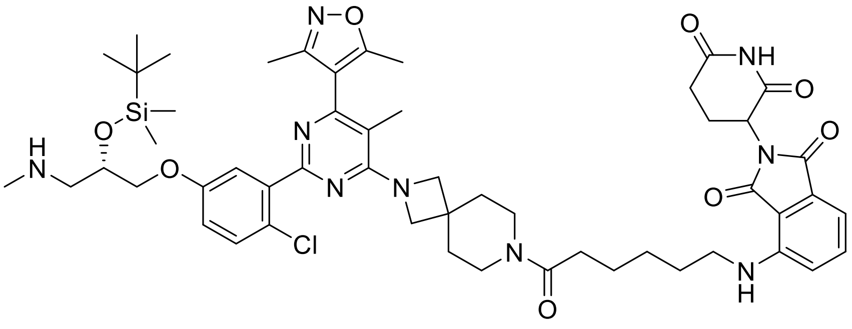

4

Figure S30. *tert*-Butyl ((2*R*)-2-((*tert*-butyldimethylsilyl)oxy)-3-(4-chloro-3-(4-(3,5-dimethylisoxazol-4-yl)-6-(7-(6-((2-(2,6-dioxopiperidin-3-yl)-1,3-dioxoisoindolin-4-yl)amino)hexanoyl)-2,7-diazaspiro[3.5]nonan-2-yl)-5-methylpyrimidin-2-yl)phenoxy)propyl)(methyl)carbamate (4). A borosilicate glass vial equipped with a magnetic stir bar was charged with **2** (65.8 mg, 0.0888 mmol) and **3** (34.4 mg, 0.0888 mmol). Dimethylformamide (3 mL) and triethylamine (50.0 µL, 0.355 mmol) were added followed by hexafluorophosphate azabenzotriazole tetramethyl uronium (50.6 mg, 0.133 mmol). The reaction mixture was stirred at room temperature for 30 minutes. Upon completion, the reaction mixture was purified by reverse-phase high-performance liquid chromatography to afford the product as yellow solid (26.2 mg, 27% yield). LCMS (ESI) $[M+H]^+$ calculated for $C_{57}H_{77}ClN_9O_{10}Si^+$m/z 1111.82, found m/z 1111.85.

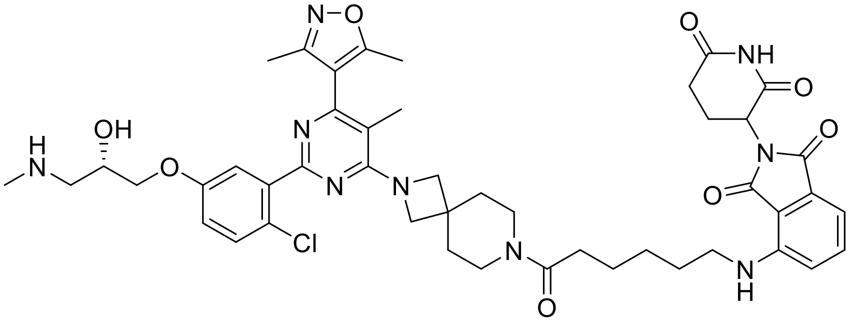

CAS-11487-074

Figure S31. 4-((6-(2-(2-(2-chloro-5-(®-2-Hydroxy-3-(methylamino)propoxy)phenyl)-6-(3,5-dimethylisoxazol-4-yl)-5-methylpyrimidin-4-yl)-2,7-diazaspiro[3.5]nonan-7-yl)-6-oxohexyl)amino)-2-(2,6-dioxopiperidin-3-yl)isoindoline-1,3-dione (CAS-11487-074).
A borosilicate glass vial equipped with a magnetic stir bar was charged with **4** (26.2 mg, 0.0236 mmol) and a solution of TFA/H_2O (1:1, 2 mL). The reaction mixture was stirred at room temperature for 16 hours. Upon completion, the reaction mixture was concentrated and purified by flash chromatography (gradient from 50:50 to 100:0 methanol with 5% triethylamine: ethyl acetate) to afford the product as a yellow solid (18.5 mg, 88% yield). LCMS (ESI) $[M+H]^+$ calculated for $C_{46}H_{55}ClN_9O_8^+$ m/z 896.44, found m/z 896.42. ^1H NMR (500 MHz, Chloroform-*d*) δ 7.52 – 7.45 (m, 1H), 7.31 (d, *J* = 8.8 Hz, 2H), 7.08 (d, *J* = 7.0 Hz, 1H), 6.88 (d, *J* = 8.6 Hz, 1H), 6.85 (dd, *J* = 8.8, 3.1 Hz, 2H), 6.25 – 6.19 (m, 1H), 4.94 – 4.86 (m, 1H), 4.32 – 4.26 (m, 1H), 4.11 (s, 4H), 4.07 – 4.01 (m, 1H), 3.99 – 3.93 (m, 1H), 3.64 (s, 7H), 3.64 – 3.57 (m, 3H), 3.45 (d, *J* = 4.8 Hz, 2H), 3.28 (dt, *J* = 13.8, 6.9 Hz, 2H), 3.11 – 2.66 (m, 9H), 2.64 (s, 2H), 2.37 (s, 3H), 2.27 (s, 3H), 2.11 (s, 3H), 1.53 – 1.44 (m, 3H), 1.38 (t, *J* = 7.2 Hz, 3H), 1.25 (s, 3H), 0.88 (t, *J* = 6.7 Hz, 1H). (missing 2 protons)

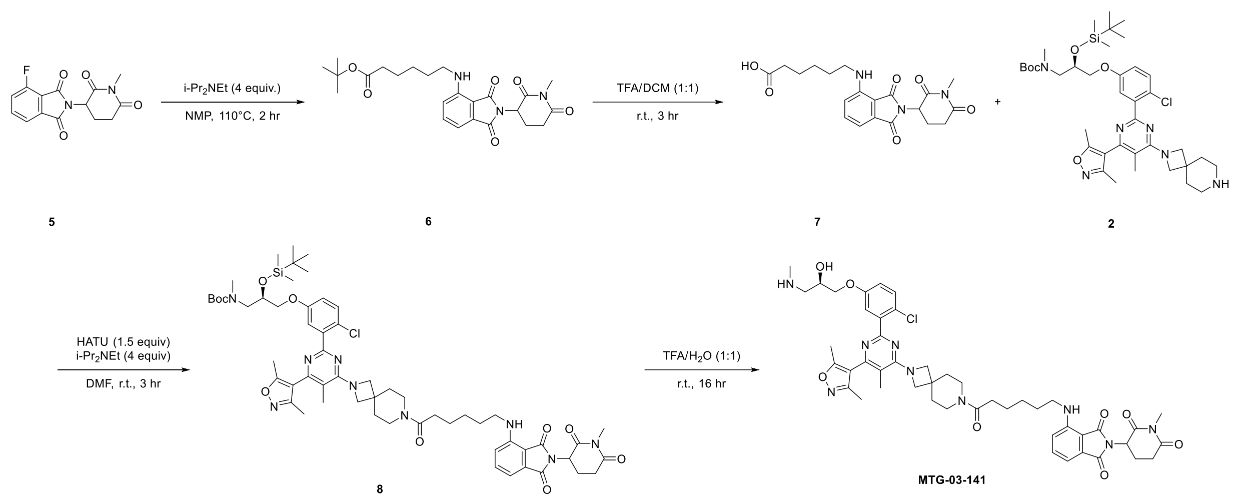

Figure S32. Synthetic Procedure and Characterization of MTG-03-141.

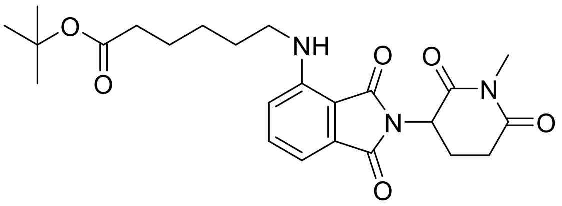

6

Figure S33. *tert*-Butyl 6-((2-(1-methyl-2,6-dioxopiperidin-3-yl)-1,3-dioxoisoindolin-4-yl)amino)hexanoate (6). To a stirred solution of **5** (142 mg, 0.487 mmol) in N-Methyl-2-Pyrrolidone (1 mL) was added N,N-Diisopropylethylamine (339 µL, 1.95 mmol) followed by *tert*-butyl 6-aminohexanoate (218 mg, 0.975 mmol). The reaction mixture was heated at 110 °C in a sand bath with stirring for 2 hours. Once complete, the reaction mixture was cooled to room temperature, resuspended in water (20 mL) and extracted with dichloromethane (3 x 20 mL). Organic layers were dried with sodium sulfate, filtered, concentrated, and purified by flash chromatography (gradient from 0:100 to 40:60 hexanes: ethyl acetate) to afford the product as a yellow oil (32.5 mg, 14% yield). LCMS (ESI) $[M+H]^+$ calculated for $C_{24}H_{32}N_3O_6^+$ - tBu m/z 402.43, found m/z 402.32.

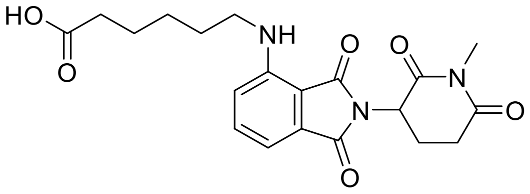

7

Figure S34. 6-((2-(1-methyl-2,6-dioxopiperidin-3-yl)-1,3-Dioxoisoindolin-4-yl)amino)hexanoic acid (7). A 1:1 solution of TFA/DCM (1 mL) was added to **6** (32.5 mg, 0.710 mmol) and stirred at room temperature for 3 hours. Once complete, the reaction mixture was concentrated to afford the crude product and used for subsequent reaction without further purification. LCMS (ESI) $[M+H]^+$ calculated for $C_{20}H_{24}N_3O_6^+$ m/z 402.42, found m/z 402.32.

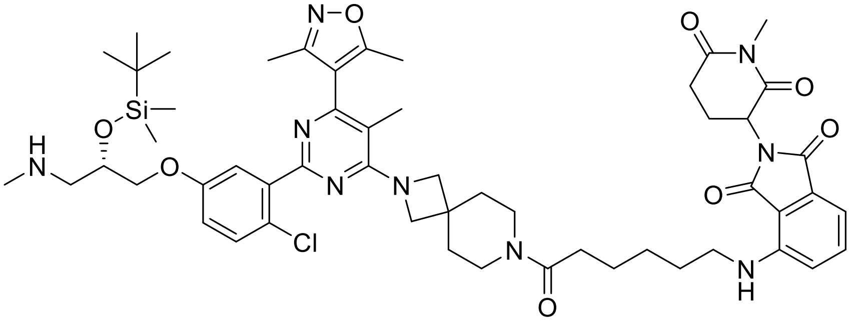

8

Figure S35. *tert*-Butyl ((2*R*)-2-((*tert*-butyldimethylsilyl)oxy)-3-(4-chloro-3-(4-(3,5-dimethylisoxazol-4-yl)-5-methyl-6-(7-(6-((2-(1-methyl-2,6-dioxopiperidin-3-yl)-1,3-dioxoisoindolin-4-yl)amino)hexanoyl)-2,7-diazaspiro[3.5]nonan-2-yl)pyrimidin-2-yl)phenoxy)propyl)(methyl)carbamate (8). To a stirred solution of **2** (57 mg, 0.0767 mmol) in dimethylformamide (1 mL) at room temperature was added **7** (31 mg, 0.0771 mmol) and N,N-Diisopropylethylamine (67.0 µL, 0.385 mmol) followed by hexafluorophosphate azabenzotriazole tetramethyl uronium (35.0 mg, 0.0925 mmol). After 3 hours, the reaction was resuspended in water (15 mL) and extracted with dichloromethane (2 x 20 mL). Organic layer was dried with sodium sulfate, filtered, concentrated, and purified by flash chromatography (gradient from 0:100 to 80:20 hexanes/ethyl acetate) to afford the product as a yellow oil (20.2 mg, 23.3% yield). LCMS (ESI) $[M+H]^+$ calculated for $C_{58}H_{79}ClN_9O_{10}Si^+$ m/z 1124.85, found m/z 1124.49.

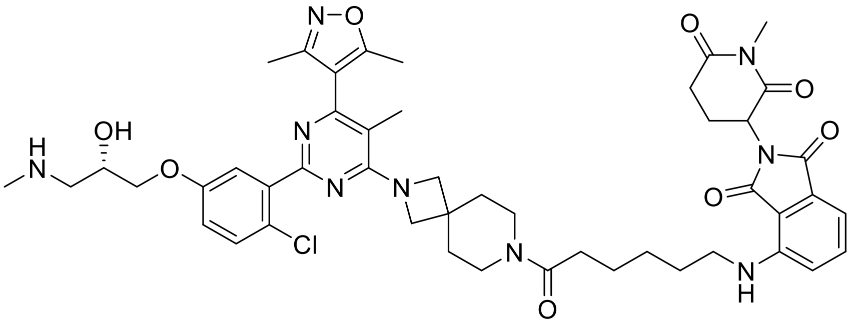

MTG-03-141

Figure S36. 4-((6-(2-(2-(2-chloro-5-((*R*)-2-Hydroxy-3-(methylamino)propoxy)phenyl)-6-(3,5-dimethylisoxazol-4-yl)-5-methylpyrimidin-4-yl)-2,7-diazaspiro[3.5]nonan-7-yl)-6-oxohexyl)amino)-2-(1-methyl-2,6-dioxopiperidin-3-yl)isoindoline-1,3-dione (MTG-03-141). A 1:1 solution of TFA/H2O (1 mL) was added to **8** (20.3 mg, 0.0180 mmol) and stirred at room temperature for 16 hours. Once completed, the reaction was concentrated and purified by reverse-phased high-performance liquid chromatography to afford the product as a yellow solid (10.1 mg,62% yield). LCMS (ESI) $[M+H]^+$ calculated for $C_{47}H_{57}ClN_9O_8^+$ m/z 910.47, found m/z 910.33.

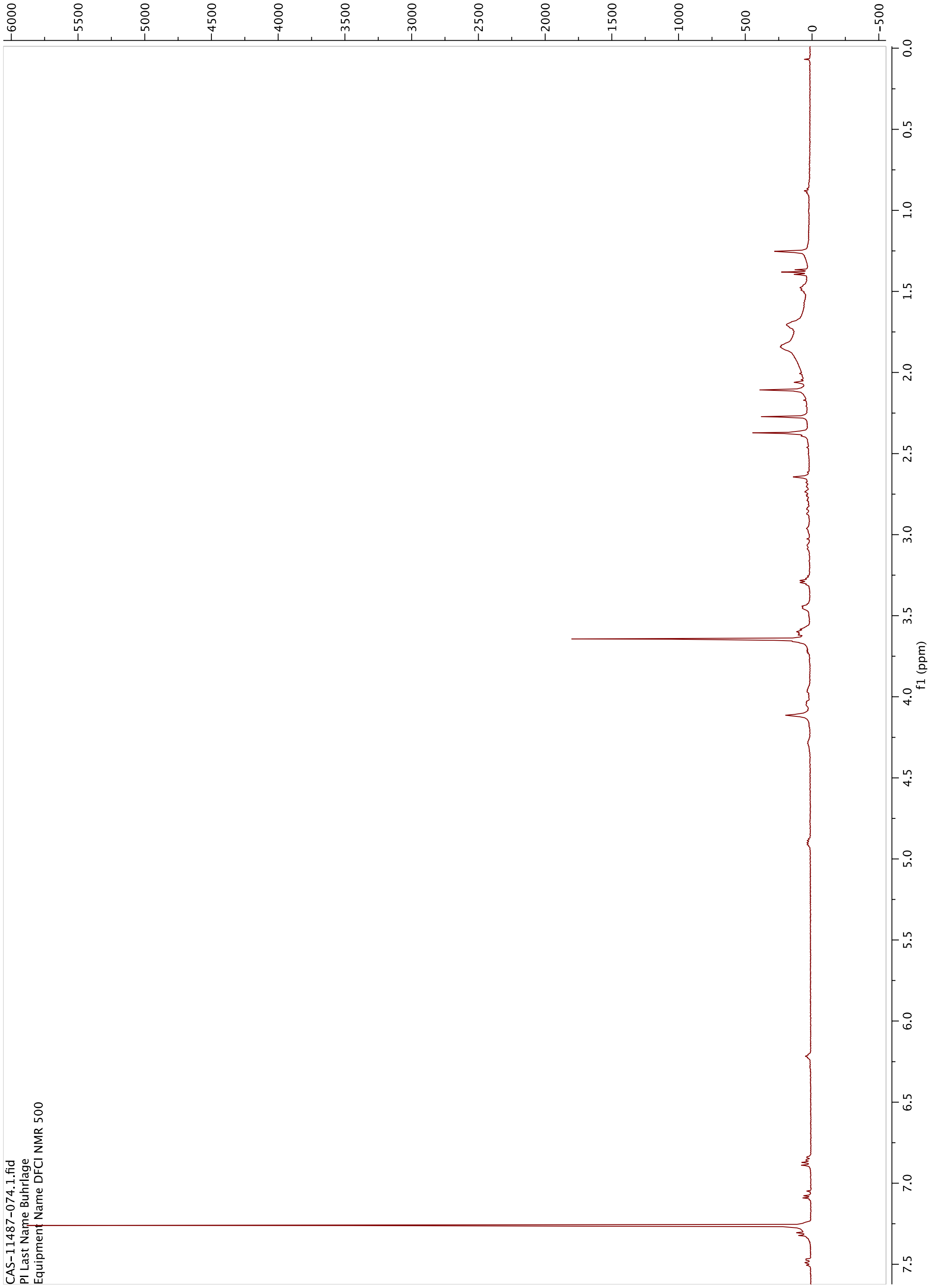

Figure S37. NMR spectrum of CAS-11487-074.

References:

1. Drew, A. E. et al. (2017). Identification of a CARM1 Inhibitor with Potent In Vitro and In Vivo Activity in Preclinical Models of Multiple Myeloma. *Scientific reports*, 7(1), 17993. https://doi-org.ezp-prod1.hul.harvard.edu/10.1038/s41598-017-18446-z
2. Patent: US10925868B2 Title: Degradation of protein kinases by conjugation of protein kinase inhibitors with E3 ligase ligand and methods of use
3. Posternak, G. et al. (2020). Functional characterization of a PROTAC directed against BRAF mutant V600E. *Nature chemical biology*, *16*(11), 1170–1178. https://doi-org.ezp-prod1.hul.harvard.edu/10.1038/s41589-020-0609-7
