# Supplementary material for: Dual CARM1-and IKZF3-targeting: A novel approach to multiple myeloma therapy synergy between CARM1 inhibition and IMiDs
Source: Mol Ther Oncol. 2025 Feb 20;33(1):200952. doi: 10.1016/j.omton.2025.200952 (PMC11930131; doi:10.1016/j.omton.2025.200952)
Supplement: Document S2. Article plus supplemental information [file mmc5.pdf]

Molecular Therapy
Oncology
Original Article

Dual CARM1-and IKZF3-targeting: A novel approach to multiple myeloma therapy synergy between CARM1 inhibition and IMiDs

Wei Ni,[1,2] Swati Garg,[1,2] Basudev Chowdhury,[1,2] Martin Sattler,[1,2] Dana Sanchez,[1] Chengcheng Meng,[1] Taisei Akatsu,[1] Katherine A. Donovan,[3,4] Jun Qi,[4] Michelle Y. Wang,[4] Cara Ann Starnbach,[3,4] Xiaoxi Liu,[3,4] Maria Tarazona Guzman,[3,4] Wei Pin Teh,[3,4] Richard Stone,[1,2] James D. Griffin,[1,2] Sara Buhrlage,[3,4] and Ellen Weisberg[1,2]

[1]Department of Medical Oncology, Dana-Farber Cancer Institute, Boston, MA, USA; [2]Department of Medicine, Harvard Medical School, Boston, MA, USA; [3]Department of Biological Chemistry and Molecular Pharmacology, Harvard Medical School, Boston, MA, USA; [4]Department of Cancer Biology, Dana-Farber Cancer Institute, Boston, MA, USA

Advancements in the treatment of multiple myeloma (MM) have resulted in an improvement in the survival rate. However, there continues to be an urgent need for improved therapies. The protein arginine methyltransferase, CARM1 (coactivator associated arginine methyltransferase 1), is emerging as a potential cancer therapy target and inhibitors have been developed. MM cell lines are particularly dependent on CARM1 for cell survival. Here, we show that targeting of CARM1 through small molecule inhibition potentiates the activity of immunomodulatory drugs (IMiDs) in cell line models of MM. This likely occurs through synergistic targeting of Aiolos (IKZF3) and MYC expression. Rational design of a new molecule, 074, which consists of a CARM1 inhibitor linked to the IMiD pomalidomide, was carried out and treatment with this agent led to more potent killing of MM cells than either the CARM1 inhibitor or the IMiD as single agents. Importantly, 074 was able to override IMiD resistance. Taken together, our results demonstrate that dual CARM1/IKZF3-targeting agents represent a promising novel therapeutic strategy for MM and IMiD-resistant disease.

INTRODUCTION

Multiple myeloma (MM) is a malignancy of clonal plasma cells.[1,2] The overall 5-year survival rate for MM has been improved to 49%–56% with the advent of immunomodulatory drugs (IMiDs) and proteasome inhibitors and other agents, such as antibodies and chimeric antigen receptor T cell therapy.[2–4] Nonetheless, drug resistance remains a problem and novel therapies are needed to provide more durable remissions.

As a protein arginine methyltransferase, coactivator-associated arginine methyltransferase 1 (CARM1) catalyzes the transfer of methyl groups from S-adenosyl methionine to side chain nitrogens of arginine residues, leading to the formation of asymmetric dimethylarginines in substrate proteins.[5] CARM1 acts through methylation of numerous substrates, in particular those with nuclear function. CARM1 has been implicated in cellular functions, including differentiation, cell-cycle regulation, transcriptional co-activation, and RNA processing.[6]

CARM1 is highly expressed in MM cells and was strongly associated with a poor prognosis.[7] CARM1 inhibitors, such as EZM2302, resulted in anti-proliferative activity in MM cell lines *in vitro* and dose-dependent tumor growth inhibition in an MM tumor xenograft model in mice,[8] thus revealing a dependency on CARM1 in MM.

The mechanism of action of CARM1 inhibitors in MM is poorly understood. Activation of p53 signaling, one of the resulting events following CARM1 inhibition, may account for the lethal effect of CARM1 inhibitors in MM.[7] A recent study investigating lineage directionality showed that CARM1-mediated methylation of arginine-35 located in the C/EBPα transcription activation domain plays a critical role in regulating B cell to macrophage transdifferentiation (BMT).[9] Moreover, it showed that mutant C/EBPα with R35A replacement, which diminishes the arginine methylation, mimicking the effect of CARM1 inhibition by the CARM1 inhibitor, TP-064, accelerates BMT by closing the chromatin for B cell genes and redistributing PU.1 to myeloid enhancers and activating macrophage genes. During the lineage program shift, the B cell lineage genes, including two essential lymphoid transcription factors in MM, IKZF1 (Ikaros) and Aiolos (IKZF3), are downregulated.[9] Nevertheless, the role of CARM1 in the expression of IKZF1/3

Received 5 October 2024; accepted 17 February 2025;
https://doi.org/10.1016/j.omton.2025.200952.

Correspondence: Sara Buhrlage, Department of Cancer Biology, Dana-Farber Cancer Institute, MA, USA.
E-mail: saraj_buhrlage@dfci.harvard.edu
Correspondence: Ellen Weisberg, Department of Medical Oncology, Dana-Farber Cancer Institute, MA, USA.
E-mail: ellen_weisberg@dfci.harvard.edu

© 2025 The Author(s).
Published by Elsevier Inc. on behalf of The American Society of Gene and Cell Therapy.
This is an open access article under the CC BY license (http://creativecommons.org/licenses/by/4.0/).

proteins in MM and its involvement in the mechanism of IMiD activity and resistance is not known.

IMiDs (lenalidomide, thalidomide, and pomalidomide) have shown clinical efficacy in MM and other B cell malignancies.[10] At present, pomalidomide is the standard-of-care treatment for relapsed/refractory MM patients after lenalidomide and a proteasome inhibitor.[11] Pomalidomide is approved for use in combination with dexamethasone, and together these agents represent a robust and synergistic therapeutic approach for relapsed and refractory MM and are being clinically evaluated as part of quadruple therapy combinations with novel agents.[11] Mechanistically, IMiDs lead to targeted ubiquitination and degradation of IKZF1 and IKZF3, via directly binding to cereblon (CRBN), which determines the substrate specificity in CRL4 E3 ubiquitin ligase complex.[12,13] By activating proteasomal degradation of IKZF1 and IKZF3, coupled with immunomodulatory effects, inhibition of angiogenesis, and disruption of MM cells with bone marrow stroma, pomalidomide exhibits potent anti-MM activity.[11]

Downregulation of IKZF1 and IKZF3 leads to decreases in MYC expression, which is essential for MM cell growth and viability.[14] Conversely, elevation in MYC levels is associated with IMiD resistance, and CRBN abnormalities, including acquired mutations of CRBN and related genes, have been identified in a portion (22%) of IMiD-resistant MM patients; both CRBN loss and MYC upregulation confer a poor prognosis.[15–17]

To increase the efficacy of IKZF1 and IKZF3 degradation induced by IMiD therapy, we identified the CARM1 inhibitor, EZM2302, as a potential drug for combination therapy. Here, we report a synergistic interaction between CARM1 inhibition and IMiDs against MM cells that correlated with the downregulation of IKZF3 and MYC. Based on this, we developed 074, a novel compound composed of the CARM1 inhibitor, EZM2302, linked to pomalidomide. 074 exhibited greater potency than either drug alone and activity against MM and IMiD-resistant MM, which correlated with downregulation of IKZF3 and MYC. Taken together, these data support the notion that dual targeting of CARM1 and IKZF3 represents a promising novel therapeutic strategy for MM and IMiD-resistant disease.

RESULTS

CARM1 inhibition potentiates the antiproliferative effects of pomalidomide against MM cells

Data from the DepMap database suggest that IKZF3 and CARM1 are independent dependencies in MM cells and that they are uniquely different from other cancers (Figure S1). We sought to define the interaction between IKZF3 and CARM1 dependencies in MM cells. To investigate this, we treated H929 and 8226 MM cell lines with the CARM1 inhibitor, EZM2302, alone and together with IKZF3-targeting pomalidomide or lenalidomide for 6 days. We observed increased sensitivity with the combination, signified by a leftward shift in the dose-response curve versus single agent-treated cells (Figures 1A–1D and S2A–S2E). Combination indices generated by Calcusyn software indicated these combinations were synergistic across a range of concentrations (Figure 1E). The activity of each agent was shown to be pathway specific, as pomalidomide treatment of MM cells degraded its known protein targets, IKZF1 and IKZF3, without demethylating BAF155 (a known substrate of CARM1), and EZM2302 treatment led to a concentration-dependent decrease in methylated BAF155 without degrading IKZF1 or IKZF3 protein levels (Figure 1F).

We were interested in identifying factors mediating the synergistic effects observed between CARM1 inhibition and IMiD treatment. Both CARM1 and Ikaros proteins are known regulators of MYC and its transcriptional network. At the concentrations used, we did not find that either drug used had a significant effect on MYC protein levels. However, levels of MYC protein were more downregulated in combination-treated H929 and 8226 cells as compared with single agent-treated cells (Figures 1G and S3A–S3C). Also, we found a modest increase in the downregulation of IKZF3 in combination-treated H929 and 8226 cells (Figures 1G and S3A–S3C; densitometry shown in Figure S16).

Doxycycline-inducible CARM1 KD in H929 cells significantly potentiated the antiproliferative effects of pomalidomide (Figures S4A and S4C), further supporting the notion of a synergistic interaction between CARM1-targeting and IMiD treatment. Conversely, doxycycline-inducible IKZF3 KD in H929 cells potentiated the antiproliferative effects of EZM2302 (Figures S4B and S4C). In addition, overexpression of IKZF3 partially rescued H929 cells from the antiproliferative effects of pomalidomide as well as the synergy observed between EZM2302 and pomalidomide (Figures S5A and S5B). These results validate the contribution of CARM1-targeting of EZM2302 and the IKZF3-targeting of pomalidomide to the synergy observed between these agents and support the notion that drug effects are on-target. Further, these results strongly suggest that IKZF3 and MYC play effector roles, at least partially, in the synergistic interaction between EZM2302 and pomalidomide.

CARM1- and Ikaros-targeting agents, 074 and 070, kill MM cells with greater potency than pomalidomide or EZM2302

We sought to design a bifunctional pomalidomide/EZM2303 chimeric small molecule drug with the potential to obtain proteolysis targeting chimera function aimed at degrading CARM1. We synthesized and screened a panel of binary compounds against MM cell lines, which varied mostly in the linker region and the E3 binding piece (either targeting CRBN or VHL). We identified compound 070 and compound 074 as being the most potent, characterized as effective dual CARM1-and IKZF3- targeting agents, however, without exhibiting CARM1 degradation. Importantly, we confirmed that the linker does not affect CARM1 ligand activity, as 074 still inhibits in an enzyme assay with a half-maximal inhibitory concentration (IC_{50}) of 2 nM (Table S3). The compounds 070 and 074 are structurally similar, the only difference being in the linker region: the linker of 074 is two carbons longer than the one in 070.

Treatment of human MM cell lines, including H929, MM.1S, and U266, with designed bivalent compounds, 070 and 074 (structures

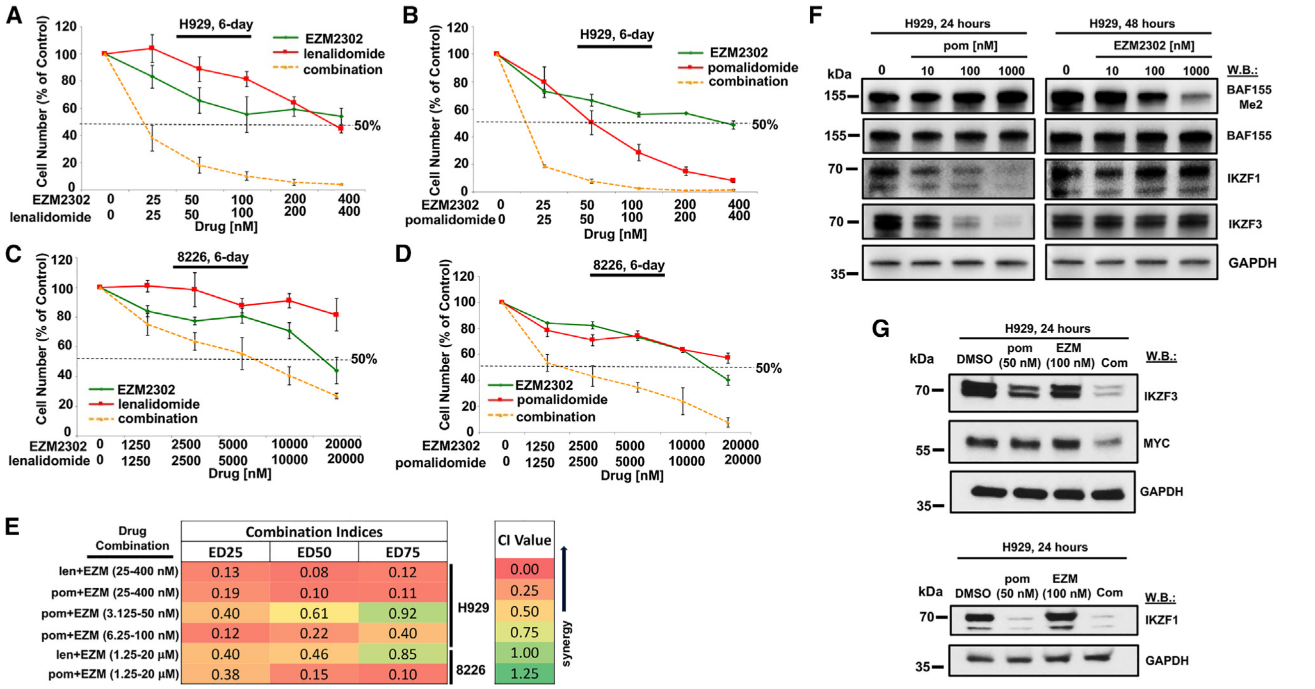

Drug Combination	Combination Indices ED25	ED50	ED75	Cell line
len+EZM (25-400 nM)	0.13	0.08	0.12	H929
pom+EZM (25-400 nM)	0.19	0.10	0.11	H929
pom+EZM (3.125-50 nM)	0.40	0.61	0.92	H929
pom+EZM (6.25-100 nM)	0.12	0.22	0.40	H929
len+EZM (1.25-20 μM)	0.40	0.46	0.85	8226
pom+EZM (1.25-20 μM)	0.38	0.15	0.10	8226

CI Value
0.00
0.25
0.50
0.75
1.00
1.25

synergy

Figure 1. CARM1 inhibition potentiates the antiproliferative effects of pomalidomide against MM cells

(A) Proliferation assay: 6-day treatment of H929 cells with EZM2303, lenalidomide, or a combination. (B) Proliferation assay: 6-day treatment of H929 cells with EZM2302, pomalidomide, or a combination. (C) Proliferation assay: 6-day treatment of 8226 cells with EZM2302, lenalidomide, or a combination. (D) Proliferation assay: 6-day treatment of 8226 cells with EZM2302, pomalidomide, or a combination. For proliferation assays, error bars represent the standard deviation for the average of n=3 or n=4 technical replicates. (E) Calcusyn combination indices (CIs). (F) Immunoblots: Effects of 24- to 48-h treatment of H929 cells with pomalidomide or EZM2302 on IKZF1, IKZF3, and methylated BAF155 expression. Results shown here are representative of other studies performed for which similar results were observed. (G) Immunoblots: Effects of 24-h treatment of H929 cells with pomalidomide, EZM2302, or a combination on IKZF1, IKZF3, and MYC protein levels.

shown in Figure 2A), each composed of EZM2302 and pomalidomide, led to selective killing of transformed cells with little to no killing of normal bone marrow cells or normal peripheral blood mononucleated cells (PBMCs) and stronger potency against MM than either EZM2302 or pomalidomide alone (Figures 2B–2D and S6A–S6I). Flow cytometric analysis performed on 074-treated, normal PBMCs to measure levels of CD19 magnetically enriched from leukopak samples revealed only modest effects at 100 nM on B cell differentiation (Figures S6J–S6L). 074 more potently suppressed the growth of H929 cells than the combination of EZM2302 and pomalidomide (Figures 2E and S7A–S7C), suggesting potential factors in addition to synergy as contributing to the drug's efficacy. It should be noted that, as 8226 cells are intrinsically IMiD resistant, the efficacy of the IMiD-EZM2302 combination requires higher concentrations of both compounds (100× higher than that required for H929 cells). However, 074 exhibits an IC_{50} of approximately 1,000 nM against 8226 cells (Figure 2C), a concentration that is minimally toxic toward normal bone marrow cells or normal PBMCs (Figures 2D, S6C, S6F, and S6J–S6L). These results suggest that 074 can potently and selectively kill IMiD-resistant MM cells at a concentration that is physiologically relevant and at concentrations that are more efficacious than those of pomalidomide combined with EZM2302. Of note, both 8226 and U266 cell lines, which express mutant p53, showed some sensitivity to 074, whereas H929 and MM.1S cells, which express wild-type p53, showed relatively higher sensitivity to 074 (Figures 2C and 2D).[18,19]

To determine whether the effects of 074 induced apoptotic cell death, H929 cells were treated with 074 for 4 days and then the percentage of apoptotic cells was measured by Annexin V and propidium iodide (PI) staining via flow cytometry. The percentage of apoptotic and necrotic cells was observed to increase in a concentration-dependent manner (Figure S8).

Both 070 and 074 treatment of H929 and 8226 cells led to a decrease in levels of methylated BAF155 that was similarly observed with EZM2302 treatment, but not observed with pomalidomide treatment (Figures 2F, 2G, and S9A–S9E). As methylated BAF155 is a substrate of CARM1, these results are indicative of on-target inhibition of CARM1 by 070 and 074. As expected, protein levels of IKZF1, IKZF3, and MYC were rapidly downregulated (within 2–24 h) with 070 and 074 treatment of H929 and 8226 cells (Figures 2F–2J, S9A, S9D, and S9F). While levels of IKZF1 and IKZF3 decreased in 074-treated 8226 cells, effects of 074 on Ikaros proteins were more modest in 8226- than in 074-treated H929 cells. 074 exhibited little to no effect on levels of CARM1 protein in H929 or 8226 cells (Figures 2H and 2I).

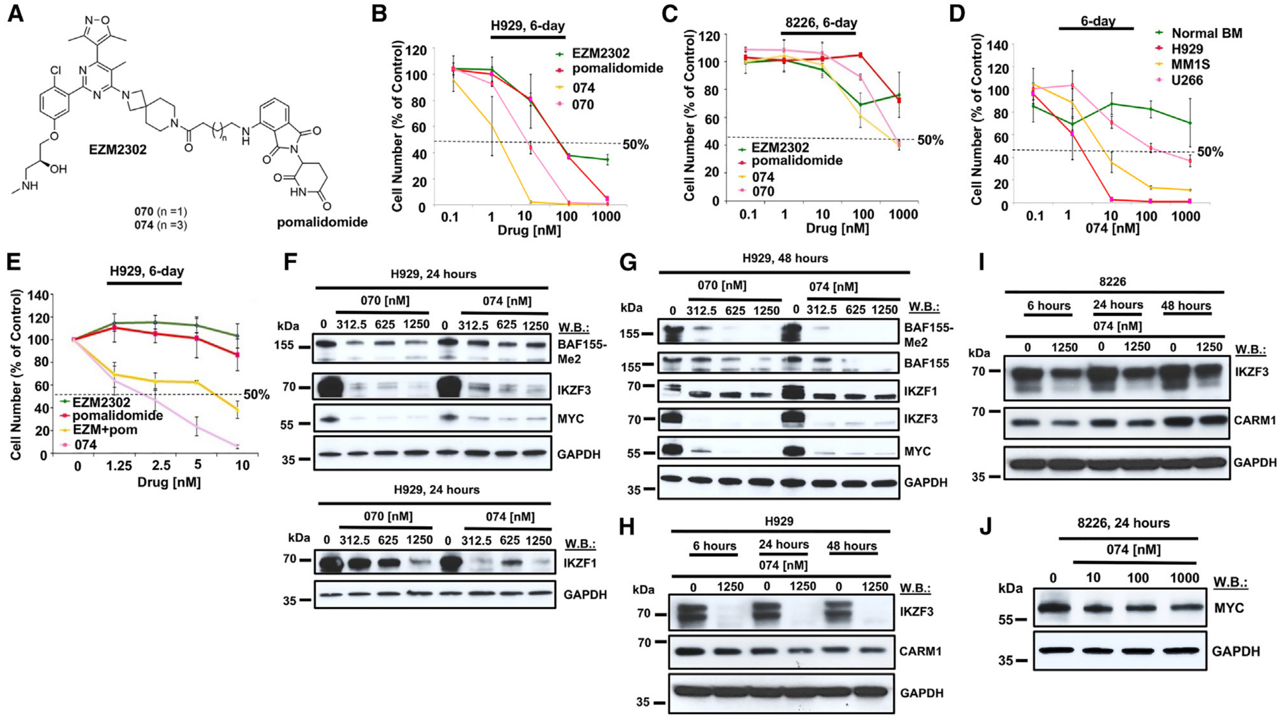

Figure 2. CARM1-and Ikaros-targeting agents, 074 and 070, potently kill MM cells with higher potency than pomalidomide or EZM2302

(A) Chemical structures of 070 and 074. (B) Proliferation assay: 6-day treatment of H929 cells with EZM2302, pomalidomide, 074, or 070. (C) Proliferation assay: 6-day treatment of 8226 cells with EZM2302, pomalidomide, 074, or 070. (D) Proliferation assay: 6-day treatment of normal BM, H929, MM.1S, and U266 cells with 074. (E) Proliferation study: 6-day treatment of H929 cells with EZM2302, pomalidomide, EZM2302 + pomalidomide, and 074. For proliferation assays, error bars represent the standard deviation for the average of n=3 or n=4 technical replicates. (F) Immunoblots: Effects of 070 and 074 on expression of methylated BAF155, IKZF1, IKZF3, and MYC after 24-h treatment of H929 cells. (G) Immunoblots: Effects of 070 and 074 on expression of methylated and total BAF155, IKZF1, IKZF3, and MYC after 48-h treatment of H929 cells. (H) Immunoblots: Effects of 074 on IKZF3 and CARM1 expression after 6-h, 24-h, and 48-h treatment of H929 cells. (I) Immunoblots: Effects of 074 on IKZF3 and CARM1 expression following 6-h, 24-h, and 48-h treatment of 8226 cells. (J) Effects of 074 on MYC expression after 24-h treatment of 8226 cells.

MYC transcript was downregulated in H929 cells treated with 074 after 24 h of treatment (Figure S9G). MYC transcript was also downregulated to a greater extent by the combination of EZM2302 and pomalidomide than either drug alone following 4 h of treatment (Figure S9I). Interferon regulatory factor 4 (IRF4), which has been shown to be important for MM cell viability and regulates MYC,[20] was decreased by 074 treatment as well after 24 h (Figure S9H). IRF4 was also downregulated to a greater extent by the combination of EZM2302 and pomalidomide than either drug alone after 4 h of treatment (Figure S9I). These results suggest that downregulation of MYC protein observed with 074 or with the combination of a CARM1 inhibitor and pomalidomide is likely due to both transcriptional and translational events.

Identification of CRBN-dependent protein targets of 074 via proteomics

To further validate the on-target IMID activity within 074, proteomics analysis of 8- and 24-h 074 treatment was performed, and we identified IKZF1, IKZF3, ZFP91, and FGD3 as primary protein degradation targets, but not CARM1 (Figures 3A and S10A). Our studies showed synergy between EZM2302 and IMiDs against MM cells (Figures 1A–1E and S2A–S2E), as well as IKZF3 and MYC to be more downregulated by the combination of EZM2302 and pomalidomide as compared with single agents (Figures 1G and S3A–S3C). Consistent with this, the dual CARM1/Ikaros targeting agent, 074, downregulated IKZF3, IKZF1, and MYC more potently than pomalidomide or EZM2302 (Figures 3B, 3C, S11A, and S11B). In addition, other protein targets of 074 identified by proteomics analysis were degraded more potently by 074 than pomalidomide or EZM2302, including zinc finger protein 91 (ZFP91), an established substrate of lenalidomide and pomalidomide,[21] that, in addition to IKZF1, has been linked to IMiD resistance,[22] and FGD3, which participates in reorganization of the actin cytoskeleton and cell shape[23] (Figures 3B, 3C, and S11B–S11E). Of note, FGD3 KD in H929 cells did not lead to changes in cell growth or viability, and a query of the DepMap database shows that FGD3 is not a disease-specific dependency in MM cell lines (Figure S10B). Levels of G1 to S phase transition 1 (GSPT1), which, similar to Ikaros, binds to CRBN,[24] were not observed to change after 8 h of treatment with 074 (Figure S11A). In summary, our results suggest that 074 hits most of the reported lenalidomide/pomalidomide targets for degradation.

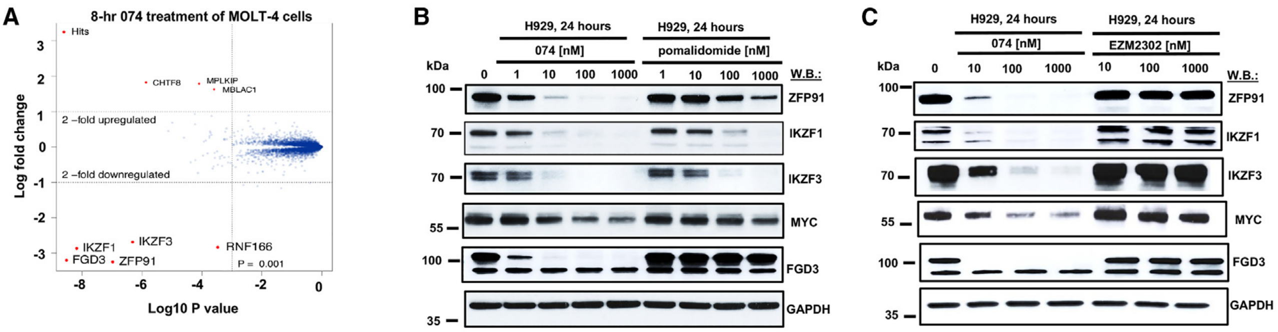

Figure 3. Identification of CRBN-dependent protein targets of 074 via proteomics

(A) Proteomics analysis for 074 treatment of MOLT-4 lymphoblast cells. (B) Immunoblots: Comparison of effects of 074 and pomalidomide on ZFP91, IKZF1, IKZF3, MYC, and FGD3 protein expression after 24 h of treatment of H929 cells. (C) Immunoblots: Comparison of effects of 074 and EZM2302 on ZFP91, IKZF1, IKZF3, MYC, and FGD3 protein expression after 24 h of treatment of H929 cells.

Validation of CRBN dependency of 074

To confirm that the observed protein downregulation by 074 occurs through a CRBN-dependent pathway, a 5-day 074 treatment of MM.1S parental cells and MM.1S CRBN knockout (KO) cells was carried out. We observed 074 at 100 nM to cause greater than 50% inhibition of cell growth of parental MM.1S cells, with higher potency than pomalidomide, but substantially less killing of MM.1S CRBN KO cells, as measured in a CellTiter Glo assay (Figures 4A and S12E). The observed potency difference correlated with CRBN-mediated degradation of the top proteins identified by proteomics analysis as main 074 targets (Figures 4B, 4C, S12A–S12D, S12F, and S12G).

The CRBN dependency of 074 was further validated by comparing antiproliferative effects of 074 with a modified version of 074 characterized by loss of CRBN binding affinity (MTG-3-141, structure and synthesis of which is described in supplemental data). 074 displayed considerably higher potency against H929 cells than MTG-3-141 in a proliferation assay (Figure 5A). In addition, whereas 074 robustly degraded the top proteins identified by proteomics analysis as main 074 targets, these proteins were not significantly degraded in H929 cells following MTG-3-141 treatment (Figures 5B and 5C). Our mass proteomic spectrometry data demonstrate that CARM1 is not a significant target degraded by 074 (Figures 3A and S10A). Consistent with this, we observed only a minor reduction or no change in CARM1 protein levels at 24-h or 48-h timepoints upon 074 or MTG-3-141 treatment (Figures 5C, S13A, and S13B). However, MTG-3-141, like 074, effectively demethylated BAF155, suggesting preservation of CARM1 inhibition (Figures S13A and S13B). Taken together, these results suggest that targeted degradation of IKZF3 by 074 is dependent on the pomalidomide moiety and therefore CRBN-dependent.

074 overrides IMiD resistance

Our data show that 074 has higher efficacy in cell line models compared with pomalidomide alone. Resistance to pomalidomide or other IMiDs is a clinical problem that is currently addressed with the clinical evaluation of related CelMods (Cereblon E3 Ligase Modulatory Drugs). We therefore tested the efficacy of 074 against H929 cells conferring resistance to pomalidomide (Figure 6A), which were developed as previously reported.[25] We have performed immunoblot analyses of CRBN, MYC, and IKZF3 expression in pomalidomide-resistant H929 cells versus H929 DMSO control cells (H929

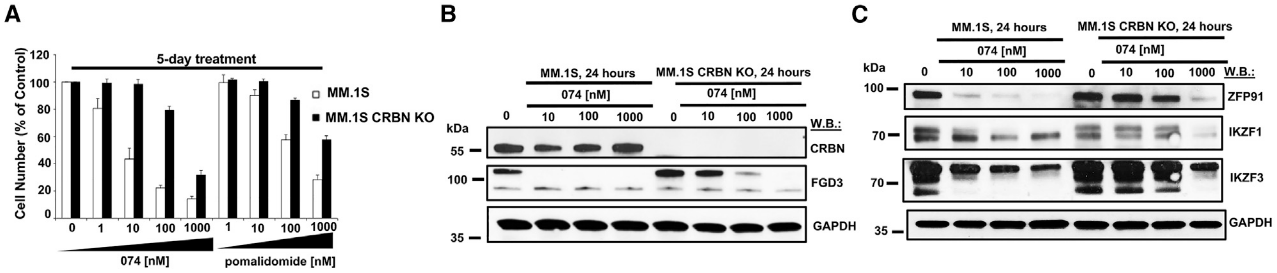

Figure 4. Effects of CRBN KO on 074 potency

(A) Proliferation study: Comparison of 074 and pomalidomide on parental MM.1S cells and MM.1S CRBN KO cells after 5 days of treatment. The MM.1S cells used were a stable line with CRBN knocked out by Crispr-Cas9. For proliferation assays, error bars represent the standard deviation for the average of n=3 or n=4 technical replicates. (B and C) Immunoblots: Comparison of effects of 074 on CRBN and FGD3 protein expression (B), and ZFP91, IKZF1, and IKZF3 protein expression (C) after 24 h of treatment of MM.1S versus MM.1S CRBN KO cells.

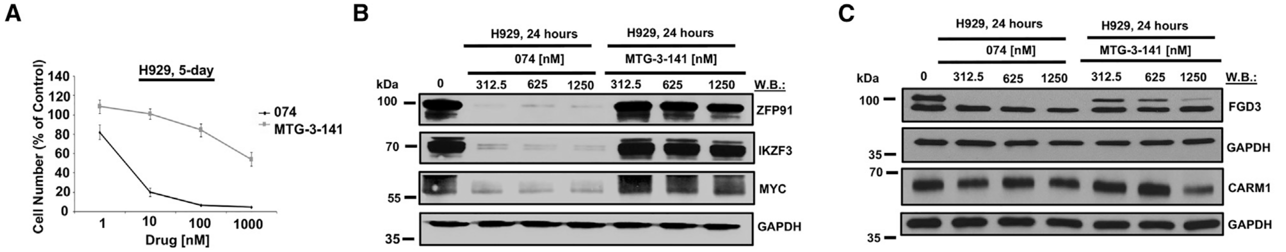

Figure 5. Effects of CRBN binding activity loss on 074 potency

(A) Proliferation study: Comparison of effects of 074 and MTG-3-141 074-CRBN negative control (without CRBN binding activity) on proliferation of H929 cells following 5 days of treatment. For proliferation assays, error bars represent the standard deviation for the average of n=3 or n=4 technical replicates. (B and C) Comparison of effects of 074 and MTG-3-141 on ZFP91, IKZF1, IKZF3, and MYC protein expression (B) and FGD3 and CARM1 protein expression (C) after 24 h of treatment.

cells treated with DMSO for 4 months). Similar to that which we have previously shown,[25] lower levels of CRBN were observed in pomalidomide-resistant H929 cells as compared with H929 DMSO control cells (Figure 6B, left), a finding consistent with an observed diminished sensitivity of CRBN KO MM.1S cells versus parental MM.1S cells to IMiDs (Figure 4A) and supportive of the CRBN dependency of IMiDs and IMiD-based agents. In addition, these results are consistent with previous reports of CRBN downregulation associated with lenalidomide resistance.[15,26] Furthermore, IKZF3 and MYC protein expression was observed to be slightly elevated in pomalidomide-resistant cells as compared with H929 DMSO control cells (Figure 6B, right). These findings coincide with an earlier report showing impaired MYC and IKZF3 downregulation by lenalidomide in lenalidomide-resistant MM cells.[26]

The combination of EZM2302 and pomalidomide tested against both H929 DMSO control cells (Figure 6C, left) and IMiD-resistant H929 cells (Figure 6C, right) caused a leftward shift in the dose-response

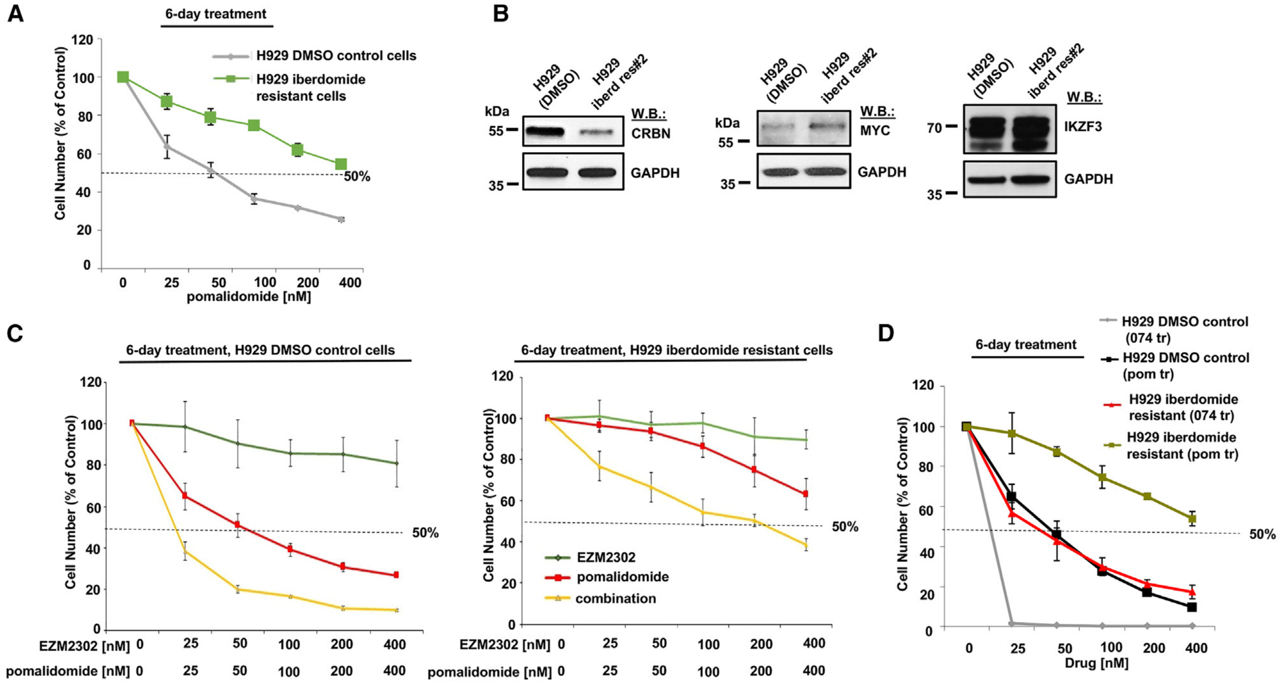

Figure 6. Effects of EZM2302 + pomalidomide against IMiD-resistant H929 cells

(A) Proliferation assay: 6-day effects of pomalidomide against H929 cells (treated with DMSO for four months) or IMiD-resistant H929 cells (treated for 4 months with up to 20 nM iberdomide). (B) Immunoblots: Comparison of levels of CRBN, MYC, and IKZF3 protein in H929 cells (treated with DMSO for 4 months) and IMiD-resistant H929 cells (treated for 4 months with up to 10–20 nM iberdomide). (C) Proliferation assays: 6-day effects of EZM2302 + pomalidomide against parental H929 cells (treated with DMSO for 4 months) (left) or iberdomide-resistant H929 cells (treated for four months with up to 20 nM iberdomide) (right). (D) Proliferation assay: 6-day effects of 074 versus pomalidomide against parental H929 cells (treated with DMSO for 4 months) or IMiD-resistant H929 cells (treated for 4 months with up to 20 nM iberdomide). For proliferation assays, error bars represent the standard deviation for the average of n=3 or n=4 technical replicates.

curve as compared with EZM2302 alone or pomalidomide alone. EZM2302 combined with pomalidomide against IMiD-resistant H929 cells (Figure 6C, right) was observed to be almost as efficacious as pomalidomide against H929 DMSO control cells (Figure 6A). Similar to pomalidomide, IMiD-resistant cells showed less sensitivity to 074 than H929 DMSO control cells (Figure 6D). However, the efficacy of 074 against IMiD-resistant H929 cells was the same as the efficacy of pomalidomide against H929 DMSO control cells (Figure 6D). These results suggest that treatment with the combination of a CARM1 inhibitor and pomalidomide, or treatment with 074, can to some extent re-sensitize cells to pomalidomide. Results also suggest that 074 activity necessarily depends on CARM1 inhibition and not only IMiD activity for growth suppression.

Taken together, these results support 074 as a unique and novel agent with properties not shared by existing therapeutic agents already in clinical use or that are under investigation as prospective MM therapies. 074 may provide needed clinical benefit in the context of some forms of IMiD resistance occurring in MM patients, and perhaps could be combined with standard therapy as a more efficacious approach to treating MM patients.

DISCUSSION

We tested the notion that targeting the MM dependency, CARM1, might enhance the CRBN-dependent, IKZF3- and IKZF1-dependent anti-MM activity of IMiDs.[27] We show synergistic interaction between CARM1 targeting through small molecule inhibition or genetic ablation plus either lenalidomide or pomalidomide against both IMiD-sensitive and IMiD-insensitive MM cells. Potentiation correlated with IKZF3 and MYC protein downregulation and was partially reversed by IKZF3 overexpression.

Synthesis of the molecules, 070 and 074, composed of EZM2302 and pomalidomide, led to substantially more potent suppression of MM cell growth than EZM2302 or pomalidomide as single agents without affecting normal bone marrow cells. This enhanced and selective growth suppression by 074 correlated with increased CRBN-dependent IKZF1, IKZF3, ZFP91, and FGD3 protein degradation, as compared with pomalidomide, which were identified in proteomics analysis as primary 074 degradation targets. Similar to EZM2302, the ability of 074 to demethylate BAF155 was preserved, suggesting 074 effectively targets CARM1. In this regard, 074 is unique as it retains CARM1 inhibition and pomalidomide activity at the same time without resulting in exclusive CARM1 degradation.

IMiD resistance is a significant challenging factor in the successful treatment of MM. Importantly, 074 was observed to be more efficacious against MM cells than the combination of pomalidomide and EZM2302. In addition, 074 killed 8226 cells, which are intrinsically resistant to IMiDs, at concentrations that were minimally toxic toward normal cells (PBMCs and bone marrow). We also used pomalidomide-resistant H929 cells, which confer acquired resistance to IMiDs, to show the ability of 074 to override IMiD resistance at physiologically relevant concentrations in MM. Specifically, we showed that 074 against IMiD-resistant MM cells was equipotent with pomalidomide against parental MM cells, suggesting that one molecule comprised of a CARM inhibitor linked to an IMiD can override IMiD resistance.

We recently reported CRBN protein levels to be downregulated in iberdomide-resistant MM cells conferring cross-resistance to pomalidomide, whereas MYC and IKZF3 protein levels were upregulated.[25] Deregulation of these proteins is consistent with CRBN deficiency and elevated levels of MYC in IMiD-resistant MM patients and impaired IMiD-induced MYC and IKZF3 downregulation in IMiD-resistant MM cells.[15,26] MYC is a known factor in the pathogenesis of MM, and our data suggest that 074 enhances the downregulation in MYC levels and so it may reduce MM growth fitness by further inhibiting MYC-mediated transcription activities. However, the involvement of CARM1-dependent mechanisms has not been fully characterized in the context of MM. CARM1 was reported to have pairwise synergistic interaction with other histone methyltransferases, like disruptor of telomeric silencing 1-like (DOT1L), jointly coordinating K562 (acute myeloid leukemia [AML]) cell survival.[28,29] A more recent study showed that diffuse large B-cell lymphoma cells with genetic lesions in genes encoding CREB-binding protein and E1A-binding protein P300 are more sensitive to CARM1 inhibition.[30] Therefore, future research prospects will focus on identifying specific genotypes or mutation burdens in MM cells that warrant either sensitivity or resistance to CARM1 inhibition, along with the underlying CARM1-dependent mechanisms.

Importantly, the downregulation of MYC protein observed with CARM1 KD + pomalidomide and observed with 074 is likely attributable to events occurring at both the transcriptional and translational levels. qPCR validated robust downregulation of MYC in MM cells treated with 074 as well as stronger downregulation of MYC transcript with EZM2302 + pomalidomide as compared with either agent alone.

In light of the fact that IRF4 has been revealed to be important for MM as a critical survival factor, and MYC has been identified as a direct target of IRF4 in MM,[20] we investigated the effect of 074 on IRF4 expression and observed downregulation of IRF4 transcript levels in 074-treated H929 cells. MYC is not solely regulated, however, by IRF4, as many other transcriptional signaling factors, such as Notch, Wnt/b-catenin/TCF, PI3K/AKT, MEK/MAPK, and Jak/STAT, can all contribute to MYC expression.[31–33] The work presented here aims to emphasize MYC as an effector of synergy between CARM1 targeting and IMiDs, as well as the activity of 074, and the observation of downregulation of IRF4 transcript in response to 074 treatment supports this.

We have recently published a study describing potentiation of IMiDs by targeting BRD9, a component of the switch/sucrose nonfermentable (SWI/SNF) chromatin-remodeling complex, noncanonical BAF.[25] In this report, we found that BRD9 KD, BRD9 inhibition, or BRD9 degradation, which lead to downregulation of MYC in some subtypes of AML and MM,[34,35] potentiated the

effects of IMiDs, including lenalidomide and pomalidomide, and conversely, IKZF3 or MYC overexpression could partially reverse this synergy. Similar to findings in the current study, we demonstrated the ability of this synergistic combination to override resistance to IMiDs in MM cells, at least in part through MYC and IKZF3 downregulation.[25]

The SWI/SNF subunit, BAF155, has been shown to be a substrate of CARM1.[36] Downregulation of MYC transcript due to CARM1 KD + pomalidomide as well as 074 treatment may at least in part be explained by the fact that CARM1-mediated BAF155 methylation affects gene expression by directing methylated BAF155 to unique chromatin regions, such as MYC pathway genes.[37] In light of these studies, as MYC plays such an integral and important role in cancer, and given its involvement in SWI/SNF signaling, it would be interesting to explore more broadly the ability of direct or indirect targeting of SWI/SNF complex subunits regulating MYC to potentiate the therapeutic effects of IMiDs for MM. As treatment of MM with IMiDs leads to ubiquitylation and proteasomal degradation of IKZF1 and IKZF3, and this results in transcriptional suppression of MYC,[14,38] it makes intuitive sense to rationally identify other targeted approaches involving MYC downregulation that have synergizing potential.

We successfully employed 074 in our *in vitro* studies, using cell lines and normal PBMCs and bone marrow, to establish a measure of therapeutic window and selectivity for transformed versus normal cells. However, we acknowledge the limitations of 074 for *in vivo* studies. Future chemistry effort will work to optimize the bioavailability of 074 for *in vivo* efficacy studies. Chemical optimization of 074 will ideally translate into agents with better *in vivo* properties that will be used to perform xenograft and PDX studies in the future.

Although newly diagnosed MM patients generally show a low incidence of *TP53* alterations, including missense mutations and 17p13 deletion, they significantly increase along the course of disease progression. *TP53* abnormalities in MM patients are associated with refractory disease and worse outcomes, especially in the cohort harboring biallelic events, double hits with concurrent del17p and mutations, which represents a clinical challenge.[16,39–42] We observed 074 to kill cell lines expressing mutated p53 in a concentration-dependent manner *in vitro*; however, the efficacy of 074 against these cell lines was more modest than the efficacy observed for 074 against wild-type p53-expressing cell lines, such as H929 and MM.1S. In addition, the decrease in Ikaros proteins by 074 was observed to be more modest in the 8226 cells, which express mutant p53, as compared with H929 cells. As activation of p53 signaling is believed to play a role in the anti-MM effects of CARM1 inhibition, and the CARM1 inhibitory activity of IMiD-based 074 is functionally preserved, it is possible that for patients with *TP53* abnormalities, maximum clinical benefit may be achieved by combining 074 with p53-targeting agents and/or other proapoptotic agents capable of overriding drug resistance due to mutated p53.[7,19]

The current study supports CARM1 as a therapeutic, druggable target for MM and introduces the novel concept of potentiation of the antitumor effects of IMiDs with CARM1 targeting. Specifically, we show synergistic interaction between CARM1 inhibition and IMiD treatment against MM, and we demonstrate that this synergy can be mimicked by a novel, rationally developed small molecule inhibitor composed of a CARM1 inhibitor and IMiD. Our findings reveal a striking dependency in MM on both CARM1 targeting and CRBN-mediated IKZF3 protein degradation, as simultaneous blockade of both pathways leads to robust MM cell killing with substantially greater potency than CARM1 inhibition or IMiD treatment alone. Our results suggest a novel and potentially highly effective therapeutic approach for MM and IMiD-resistant MM.

MATERIALS AND METHODS

Chemical compounds and biologic reagents

EZM2302 was purchased from MedChemExpress (Cat. No.: HY-111109). Lenalidomide was purchased from MedChemExpress (ID#: HY-A0003). Pomalidomide was purchased from Millipore Sigma (P0018-5MG) and MedChemExpress (Cat. No.: HY-10984). The agents, 070, 074, and MTG-3-141 (negative control compound), were synthesized by Dr. Sara Buhrlage (details provided in supplemental data section).

Cell lines and cell culture

H929, 8226, MM.1S, and U266 MM cell lines were obtained from Dr. Kenneth Anderson (Dana-Farber Cancer Institute). All cell lines were cultured at 37°C with 5% CO_2 (2×10^5–5×10^5 cells/mL) in RPMI 1640 media, purchased from Gibco (cat #11875-093). Media was supplemented with 10% fetal bovine serum (FBS), purchased from Gibco (cat # 10437-028) and 1% penicillin/streptomycin (5,000 U/mL), purchased from Gibco (cat # 15070063). Culture media for H929 was supplemented with 2-mercaptoethanol (50 μM).

All cell lines were authenticated within six months of manuscript preparation via cell line short tandem repeat (STR) profiling (Molecular Diagnostics Core, Dana-Farber Cancer Institute). Tested cell lines matched 80% or greater with lines listed in the DSMZ Cell Line Bank STR database (https://www.dsmz.de/catalogues/catalogue-human-and-animal-cell-lines/quality-assurance/identity-control/authentication-of-cell-lines.html). Cell lines were virus and mycoplasma free.

Cell proliferation studies

Cell counting was performed using the Trypan blue exclusion assay before seeding for CellTiter-Glo (Promega) proliferation assays. Studies were carried out according to manufacturer's instructions. Cell viability is graphed as the percentage of control (untreated) cells with error bars representing standard deviations (n = 3 or 4).

Cell viability studies

Cell viability was measured using the FITC Annexin V Apoptosis Detection Kit with PI (catalog #640914) (BioLegend) as per the manufacturer's instructions.

Drug combination studies

Single agents were added at fixed ratios and simultaneously to cells. Cell number was expressed as a function of drug-treated and growth affected cells versus DMSO vehicle control cells. Calcusyn software (Biosoft), which is based on isobologram generation,[43] was used to assess synergy or antagonism as previously described.[35]

Normal donor-derived PBMC studies

PBMCs derived from a normal donor were attained from the Specimen Bank, Brigham and Women's Hospital under approval of the Dana-Farber Cancer Institute Institutional Review Board. Samples were subjected to Ficoll-purification to obtain mononuclear cells and cultured with 5% CO_2 (2×10^5–5×10^5 cells/mL) at 37°C in RPMI 1640 media (Gibco cat #11875-093). Media was supplemented with 10% FBS (Gibco cat # 10437-028) and 1% penicillin/streptomycin (5,000 U/mL) (Gibco cat # 15070063). For CellTiter-Glo (Promega) proliferation assays, PBMCs were seeded at 20,000 cells/well and treated with 074 at the indicated concentrations for 4 days. Studies were carried out according to the manufacturer's instructions.

Flow cytometry analysis and CD19$^+$ B cell enrichment

Healthy donor leuko-reduction samples were collected at Brigham and Women's Hospital's specimen bank under the approved protocol as per the ethical guidelines of the institutional review board. Mono-nuclear cells were enriched using Ficoll density gradient (GE #17-5442-02) and subjected to CD19$^+$ B cells enrichment using microbeads (Miltenyi #130-050-301) and placed in RPMI supplemented with 10% heat inactivated FBS and PenStrep. Cells were placed into 5–6 replicates per condition in 96-well plates for a viability assay and on day 6 after treatment, after addition of DAPI staining solution, data were acquired on high-throughput screening (HTS) fluorescence-activated cell sorting (FACS). For the differentiation assay, triplicates per condition were treated in 24-well plates and cells were collected on day 3 after treatment, washed with PBS and after addition of Human TruStain FcX (#422302) stained with antibodies from BioLegend Alexa Fluor 647 anti-human CD27 (#302812), PE anti-human IgD (#348204). Data were acquired on BD LSRFortessa and analyzed with Flowjo version 10 where time gated events were filtered for live (DAPI negative), single cells for further analysis.

Doxycycline-inducible CARM1 knockdown H929 cells and doxycycline-inducible IKZF3 knockdown H929 cells

Generation of human MM cells expressing doxycycline-inducible shRNAs targeting CARM1 *and* IKZF3 *genes*

FH1tUTG, the doxycycline-inducible lentiviral expression vector, is a generous gift from Dr. Lizi Wu laboratory at the University of Florida and was originally obtained from Dr. Marco J. Herold at The Walter and Eliza Hall Institute.[44] The vector was sequentially cut by restriction enzymes, *Bsm*BI and *Xho*I. Two sets of oligonucleotides that contain shRNAs targeting *CARM1* and *IKZF3* respectively were annealed and inserted into the purified vector with *Bsm*BI/*Xho*I sticky ends.

For doxycycline-inducible KD of *CARM1*, the following oligo sequences were used: ishCARM1-1 (forward, 5′-TCCCCGATTTCTG TTCCTTCTACAACTCGAGTTGTAGAAGGAACAGAAATCGTT TTTC-3′, and reverse, 5′-TCGAGAAAAACGATTTCTGTTCCTTCT ACAACTCGAGTTGTAGAAGGAACAGAAATCG-3′); ishCARM1-2 (forward, 5′- TCCCCTATGACTTGAGCAGTGTTATCTCGAGAT AACACTGCTCAAGTCATAGTTTTTC-3′, and reverse, 5′-TCGA GAAAAACTATGACTTGAGCAGTGTTATCTCGAGATAACACT GCTCAAGTCATAG-3′).

For doxycycline-inducible KD of *IKZF3*, the following oligo sequences were used: ishIKZF3-1 (forward, 5′-TCCCGTAACCTCCT CCGCCACATTACTCGAGTAATGTGGCGGAGGAGGTTACTTT TTC-3′, and reverse, 5′-TCGAGAAAAAGTAACCTCCTCCGCCAC ATTACTCGAGTAATGTGGCGGAGGAGGTTAC-3′); ishIKZF3-3 (forward, 5′- TCCCGCCAATGAAGATGAAGACATACTCGAGTA TGTCTTCATCTTCATTGGCTTTTTC-3′, and reverse, 5′-TCGAG AAAAAGCCAATGAAGATGAAGACATACTCGAGTATGTCTTC ATCTTCATTGGC-3′).

The subcloned vectors were validated by sanger sequencing with FH1t primer (5′- TCGCTATGTGTTCTGGGAAA-3′). For lentiviral production, the established FH1tUTG-ishCARM1 or -ishIKZF3 constructs along with packaging plasmid psPAX2 (Addgene) and envelope plasmid pMD2.G (Addgene) were co-transfected to HEK293T cells using Effectene transfection kit (Qiagen). The culture suspension that contains lentiviral particles was harvested at 72 h after transfection and filtered through 0.45-μm PES filters and stored at −80°C for future use. Human MM cell line, H929, was infected following a spinoculation protocol as described by Broad Institute (https://portals.broadinstitute.org/gpp/public/resources/protocols). At 96 h after infection, the FH1tUTG-containing GFP-positive H929 cells were sorted by flow cytometry using a BD Aria-II sorter.

IKZF3-overexpressing H929 cells

H929 stable overexpression

For the stable overexpression of open reading frame (ORF) sequences, pLVX-EF1α-IRES-mCherry Vector was purchased from Takara (#631987) and the full-length sequence of EF1α promoter was replaced with custom synthesized EF1α core promoter. ORF containing cDNA sequence of IKZF1 (NM_006060.6 # OHu28071) and IKZF3(NM_012481 # OHU21008D) in pCDNA vectors were purchased from GenScript and subcloned between EcoRI and SpeI sites into the custom pLVX-EF1α core-MCS-IRES-mCherry vector. To produce high titer lentivirus in Lenti-X 293T HEK cells from Takara (#632180) cells were transfected using Turbofect (Thermo#R0532), plasmid of interest and packaging plasmids PMD2.G (#12259), and psPAX2 (#12260) from Addgene. Virus supernatants were collected at 48 and 72 h after transfection, filtered through 0.4-micron filters, and ultra-centrifuged through 0.2-micron filtered 20% sucrose before collection into 80 mL of pure IMDM. Virus particles were titrated on MV4;11 AML cell line. H929 cells were spin-infected with empty vector control and IKZF3 overexpression virus at multiplicity of infection of 5 at 1,800 rpm for 30 min using 8 μg/mL polybrene. Cells

were selected for mCherry production at day 3 by FACS, expanded and validated for protein overexpression by western blot and RT-PCR before use in downstream assays.

Immunoblotting

Protein lysate preparation and immunoblotting were carried out as has been previously described.[45]

GAPDH antibody (14C10) (rabbit mAb, #2118) (1:2,000), MYC antibody (D84C12) (rabbit mAb, #5605) (1:1,000), SMARCC1/BAF155 antibody (D7F8S) (rabbit mAb, #11956) (1:1,000), Ikaros (D6N9Y) antibody (rabbit mAb, #14859) (1:1,000), IKZF3 antibody (D1C1E) (rabbit mAb, #15103) (1:1,000) and CRBN antibody (D8H3S) (rabbit mAb, #71810) (1:1,000) were purchased from Cell Signaling Technology.

Dimethyl-BAF155 antibody (ABE1399) (1:1,000), β-actin (mouse monoclonal) antibody (clone AC-15, #A1978) (1:1,000), and CARM1 antibody (09–818) (1:1,000) were purchased from Millipore-Sigma. FGD3 antibody (20347-1-AP, rabbit pAb) (1:1.000–1:5.000) was purchased from ProteinTech and ZFP91 antibody (A303-245A, rabbit pAb) (1:2,000) was purchased from Bethyl. All antibodies were used at the listed dilutions in the presence of 5% milk.

The original images of all immunoblots are shown in Figures S14–S27. Densitometric analyses of all immunoblots are also shown in Figures S14–S27.

Proteomics

Treatment with 074 was performed at 2.5 μM in MOLT4 cells at 8 and 24 h.

This is a multiplexed quantitative proteomics experiment used for assessing protein abundance changes in response to degraders.[46] Raw data for the 8- and 24-h timepoints are provided in Tables S1 and S2, respectively.

Densitometry

ImageJ package was used to analyze the densitometric values (raw reads) for immunoblotting bands. For the normalization results, each blot was normalized to the corresponding very left blot (usually the control) in each group.

DATA AVAILABILITY

For access to proteomics data, the PRIDE archive ID is: PXD046700.

ACKNOWLEDGMENTS

We would like to thank Dr. Kareem Azab for his thoughtful insights and helpful scientific discussions. This work was funded by NIH grant P01 CA 066996 (05/01/2020-4/30/2025).

AUTHOR CONTRIBUTIONS

W.N. wrote paper and designed the research study; performed immunoblots and developed CARM1 KD and IKZF3 KD cells. S.G. developed and provided IKZF3 overexpressing cells. B.C. developed and performed immunoblots and RNA-seq analysis. M.S. wrote the paper and designed the research study and validated KD hairpins and designed the inducible KD construct based on the efficacy of shRNA in growth assays. D.S. Performed immunoblots and qPCR. C.M. performed immunoblots and qPCR. K.D. performed proteomics study. C.A.S. performed chemical synthesis of 070 and 074. X.L. assisted with proteomics analysis. M.T.G. resynthesized 074 and synthesized the negative control compound, MTG-3-141. W.P. resynthesized 074 and synthesized the negative control compound, MTG-3 141. R.S. designed the research study. S.B. wrote the paper and designed and performed chemical synthesis of 070 and 074. J.D.G. designed the research study. E.W. wrote the paper and designed the research study; performed proliferation studies and synergy studies.

DECLARATION OF INTERESTS

There is no conflict of interest to report. However, for full disclosure, we are providing the following details for several authors on this manuscript.

J.D.G. receives funding and has received a royalty payment from Novartis Pharmaceuticals and receives funding from Eli Lilly and Company. R.S. does Ad Hoc consulting for and receives clinical research support to Dana-Farber Cancer Institute from the following companies: Abbvie, Agios, Arog, and Novartis. He does Ad Hoc consulting for the following companies: Astrazeneca, Cornerstone, Jazz, Daiichi-Sankyo, Otsuka/Astex, Pfizer, and Stemline. He is on the Advisory Board of the following companies: Actinium, Amgen, Astellas, and Macrogenics. He is on the Data Safety and Monitoring Board for the following companies: Argenx, Celgene, and Takeda. He is an Ad Hoc Consultant and on the Steering Committee and Data Safety and Monitoring Board for Celgene. S.B. is a founder and SAB member of Entact Bio. She also served on the SABs of Oncovalent and Adenoid Cystic Carcinoma Foundation. She receives funding from AbbVie, Tuo, and Takeda. K.A.D. is a consultant to Kronos Bio and Neomorph Inc.

SUPPLEMENTAL INFORMATION

Supplemental information can be found online at https://doi.org/10.1016/j.omton.2025.200952.

REFERENCES

1. Reisenbuckler, C. (2014). Multiple myeloma and diagnostic imaging. Radiol. Technol. *85*, 391–413. quiz 411-393.
2. Kazandjian, D. (2016). Multiple myeloma epidemiology and survival: A unique malignancy. Semin. Oncol. *43*, 676–681. https://doi.org/10.1053/j.seminoncol.2016.11.004.
3. Sadek, N.L., Costa, B.A., Nath, K., and Mailankody, S. (2023). CAR T-Cell Therapy for Multiple Myeloma: A Clinical Practice-Oriented Review. Clin. Pharmacol. Ther. *114*, 1184–1195. https://doi.org/10.1002/cpt.3057.
4. Iftikhar, A., Hassan, H., Iftikhar, N., Mushtaq, A., Sohail, A., Rosko, N., Chakraborty, R., Razzaq, F., Sandeep, S., Valent, J.N., et al. (2019). Investigational Monoclonal Antibodies in the Treatment of Multiple Myeloma: A Systematic Review of Agents under Clinical Development. Antibodies *8*, 34. https://doi.org/10.3390/antib8020034.
5. Chen, D., Ma, H., Hong, H., Koh, S.S., Huang, S.M., Schurter, B.T., Aswad, D.W., and Stallcup, M.R. (1999). Regulation of transcription by a protein methyltransferase. Science *284*, 2174–2177. https://doi.org/10.1126/science.284.5423.2174.
6. Shishkova, E., Zeng, H., Liu, F., Kwiecien, N.W., Hebert, A.S., Coon, J.J., and Xu, W. (2017). Global mapping of CARM1 substrates defines enzyme specificity and substrate recognition. Nat. Commun. *8*, 15571. https://doi.org/10.1038/ncomms15571.
7. Yang, L., Ma, L., Gong, Q., Chen, J., and Huang, Q. (2023). Inhibition of CARM1 suppresses proliferation of multiple myeloma cells through activation of p53 signaling pathway. Mol. Biol. Rep. *50*, 7457–7469. https://doi.org/10.1007/s11033-023-08645-5.
8. Drew, A.E., Moradei, O., Jacques, S.L., Rioux, N., Boriack-Sjodin, A.P., Allain, C., Scott, M.P., Jin, L., Raimondi, A., Handler, J.L., et al. (2017). Identification of a CARM1 Inhibitor with Potent In Vitro and In Vivo Activity in Preclinical Models of Multiple Myeloma. Sci. Rep. *7*, 17993. https://doi.org/10.1038/s41598-017-18446-z.
9. Torcal Garcia, G., Kowenz-Leutz, E., Tian, T.V., Klonizakis, A., Lerner, J., De Andres-Aguayo, L., Sapozhnikova, V., Berenguer, C., Carmona, M.P., Casadesus, M.V., et al. (2023). Carm1-arginine methylation of the transcription factor C/EBPalpha regulates transdifferentiation velocity. Elife *12*, e83951. https://doi.org/10.7554/eLife.83951.

10. Rajkumar, S.V., Hayman, S.R., Lacy, M.Q., Dispenzieri, A., Geyer, S.M., Kabat, B., Zeldenrust, S.R., Kumar, S., Greipp, P.R., Fonseca, R., et al. (2005). Combination therapy with lenalidomide plus dexamethasone (Rev/Dex) for newly diagnosed myeloma. Blood *106*, 4050–4053. https://doi.org/10.1182/blood-2005-07-2817.

11. Fotiou, D., Gavriatopoulou, M., Terpos, E., and Dimopoulos, M.A. (2022). Pomalidomide- and dexamethasone-based regimens in the treatment of refractory/relapsed multiple myeloma. Ther. Adv. Hematol. *13*, 20406207221090089. https://doi.org/10.1177/20406207221090089.

12. Kronke, J., Udeshi, N.D., Narla, A., Grauman, P., Hurst, S.N., McConkey, M., Svinkina, T., Heckl, D., Comer, E., Li, X., et al. (2014). Lenalidomide causes selective degradation of IKZF1 and IKZF3 in multiple myeloma cells. Science *343*, 301–305. https://doi.org/10.1126/science.1244851.

13. Ito, T., Ando, H., Suzuki, T., Ogura, T., Hotta, K., Imamura, Y., Yamaguchi, Y., and Handa, H. (2010). Identification of a primary target of thalidomide teratogenicity. Science *327*, 1345–1350. https://doi.org/10.1126/science.1177319.

14. Greco, C., D'Agnano, I., Vitelli, G., Vona, R., Marino, M., Mottolese, M., Zuppi, C., Capoluongo, E., and Ameglio, F. (2006). c-MYC deregulation is involved in melphalan resistance of multiple myeloma: role of PDGF-BB. Int. J. Immunopathol. Pharmacol. *19*, 67–79.

15. Franssen, L.E., Nijhof, I.S., Couto, S., Levin, M.D., Bos, G.M.J., Broijl, A., Klein, S.K., Ren, Y., Wang, M., Koene, H.R., et al. (2018). Cereblon loss and up-regulation of c-Myc are associated with lenalidomide resistance in multiple myeloma patients. Haematologica *103*, e368–e371. https://doi.org/10.3324/haematol.2017.186601.

16. Kortum, K.M., Mai, E.K., Hanafiah, N.H., Shi, C.X., Zhu, Y.X., Bruins, L., Barrio, S., Jedlowski, P., Merz, M., Xu, J., et al. (2016). Targeted sequencing of refractory myeloma reveals a high incidence of mutations in CRBN and Ras pathway genes. Blood *128*, 1226–1233. https://doi.org/10.1182/blood-2016-02-698092.

17. Gooding, S., Ansari-Pour, N., Towfic, F., Ortiz Estévez, M., Chamberlain, P.P., Tsai, K.T., Flynt, E., Hirst, M., Rozelle, D., Dhiman, P., et al. (2021). Multiple cereblon genetic changes are associated with acquired resistance to lenalidomide or pomalidomide in multiple myeloma. Blood *137*, 232–237. https://doi.org/10.1182/blood.2020007081.

18. Petrusca, D.N., Toscani, D., Wang, F.M., Park, C., Crean, C.D., Anderson, J.L., Marino, S., Mohammad, K.S., Zhou, D., Silbermann, R., et al. (2018). Growth factor independence 1 expression in myeloma cells enhances their growth, survival, and osteoclastogenesis. J. Hematol. Oncol. *11*, 123. https://doi.org/10.1186/s13045-018-0666-5.

19. Jones, R.J., Bjorklund, C.C., Baladandayuthapani, V., Kuhn, D.J., and Orlowski, R.Z. (2012). Drug resistance to inhibitors of the human double minute-2 E3 ligase is mediated by point mutations of p53, but can be overcome with the p53 targeting agent RITA. Mol. Cancer Therapeut. *11*, 2243–2253. https://doi.org/10.1158/1535-7163.MCT-12-0135.

20. Shaffer, A.L., Emre, N.C.T., Lamy, L., Ngo, V.N., Wright, G., Xiao, W., Powell, J., Dave, S., Yu, X., Zhao, H., et al. (2008). IRF4 addiction in multiple myeloma. Nature *454*, 226–231. https://doi.org/10.1038/nature07064.

21. An, J., Ponthier, C.M., Sack, R., Seebacher, J., Stadler, M.B., Donovan, K.A., and Fischer, E.S. (2017). pSILAC mass spectrometry reveals ZFP91 as IMiD-dependent substrate of the CRL4(CRBN) ubiquitin ligase. Nat. Commun. *8*, 15398. https://doi.org/10.1038/ncomms15398.

22. Wu, W., Nelson, G.M., Koch, R., Donovan, K.A., Nowak, R.P., Heavican-Foral, T.B., Nirmal, A.J., Liu, H., Yang, L., Duffy, J., et al. (2022). Overcoming IMiD resistance in T-cell lymphomas through potent degradation of ZFP91 and IKZF1. Blood *139*, 2024–2037. https://doi.org/10.1182/blood.2021014701.

23. Nakanishi, H., and Takai, Y. (2008). Frabin and other related Cdc42-specific guanine nucleotide exchange factors couple the actin cytoskeleton with the plasma membrane. J. Cell Mol. Med. *12*, 1169–1176. https://doi.org/10.1111/j.1582-4934.2008.00345.x.

24. Matyskiela, M.E., Lu, G., Ito, T., Pagarigan, B., Lu, C.C., Miller, K., Fang, W., Wang, N.Y., Nguyen, D., Houston, J., et al. (2016). A novel cereblon modulator recruits GSPT1 to the CRL4(CRBN) ubiquitin ligase. Nature *535*, 252–257. https://doi.org/10.1038/nature18611.

25. Chowdhury, B., Garg, S., Ni, W., Sattler, M., Sanchez, D., Meng, C., Akatsu, T., Stone, R., Forrester, W., Harrington, E., et al. (2024). Synergy between BRD9- and IKZF3-Targeting as a Therapeutic Strategy for Multiple Myeloma. Cancers *16*, 1319. https://doi.org/10.3390/cancers16071319.

26. Zhu, Y.X., Shi, C.X., Bruins, L.A., Wang, X., Riggs, D.L., Porter, B., Ahmann, J.M., de Campos, C.B., Braggio, E., Bergsagel, P.L., and Stewart, A.K. (2019). Identification of lenalidomide resistance pathways in myeloma and targeted resensitization using cereblon replacement, inhibition of STAT3 or targeting of IRF4. Blood Cancer J. *9*, 19. https://doi.org/10.1038/s41408-019-0173-0.

27. Cippitelli, M., Stabile, H., Kosta, A., Petillo, S., Gismondi, A., Santoni, A., and Fionda, C. (2021). Role of Aiolos and Ikaros in the Antitumor and Immunomodulatory Activity of IMiDs in Multiple Myeloma: Better to Lose Than to Find Them. Int. J. Mol. Sci. *22*, 1103. https://doi.org/10.3390/ijms22031103.

28. Nakayama, K., Szewczyk, M.M., Dela Sena, C., Wu, H., Dong, A., Zeng, H., Li, F., de Freitas, R.F., Eram, M.S., Schapira, M., et al. (2018). TP-064, a potent and selective small molecule inhibitor of PRMT4 for multiple myeloma. Oncotarget *9*, 18480–18493. https://doi.org/10.18632/oncotarget.24883.

29. Han, K., Jeng, E.E., Hess, G.T., Morgens, D.W., Li, A., and Bassik, M.C. (2017). Synergistic drug combinations for cancer identified in a CRISPR screen for pairwise genetic interactions. Nat. Biotechnol. *35*, 463–474. https://doi.org/10.1038/nbt.3834.

30. Veazey, K.J., Cheng, D., Lin, K., Villarreal, O.D., Gao, G., Perez-Oquendo, M., Van, H.T., Stratton, S.A., Green, M., Xu, H., et al. (2020). CARM1 inhibition reduces histone acetyltransferase activity causing synthetic lethality in CREBBP/EP300-mutated lymphomas. Leukemia *34*, 3269–3285. https://doi.org/10.1038/s41375-020-0908-8.

31. Gumede, D.B., Abrahamse, H., and Houreld, N.N. (2024). Targeting Wnt/beta-catenin signaling and its interplay with TGF-beta and Notch signaling pathways for the treatment of chronic wounds. Cell Commun. Signal. *22*, 244. https://doi.org/10.1186/s12964-024-01623-9.

32. Lu, Q., Yang, D., Li, H., Niu, T., and Tong, A. (2024). Multiple myeloma: signaling pathways and targeted therapy. Mol. Biomed. *5*, 25. https://doi.org/10.1186/s43556-024-00188-w.

33. de Cassia Viu Carrara, R., Fontes, A.M., Abraham, K.J., Orellana, M.D., Haddad, S.K., Palma, P.V.B., Panepucci, R.A., Zago, M.A., and Covas, D.T. (2018). Expression differences of genes in the PI3K/AKT, WNT/b-catenin, SHH, NOTCH and MAPK signaling pathways in CD34+ hematopoietic cells obtained from chronic phase patients with chronic myeloid leukemia and from healthy controls. Clin. Transl. Oncol. *20*, 542–549. https://doi.org/10.1007/s12094-017-1751-x.

34. Hohmann, A.F., Martin, L.J., Minder, J.L., Roe, J.S., Shi, J., Steurer, S., Bader, G., McConnell, D., Pearson, M., Gerstberger, T., et al. (2016). Sensitivity and engineered resistance of myeloid leukemia cells to BRD9 inhibition. Nat. Chem. Biol. *12*, 672–679. https://doi.org/10.1038/nchembio.2115.

35. Weisberg, E., Chowdhury, B., Meng, C., Case, A.E., Ni, W., Garg, S., Sattler, M., Azab, A.K., Sun, J., Muz, B., et al. (2022). BRD9 degraders as chemosensitizers in acute leukemia and multiple myeloma. Blood Cancer J. *12*, 110. https://doi.org/10.1038/s41408-022-00704-7.

36. Wang, X., and Roberts, C.W. (2014). CARMA: CARM1 methylation of SWI/SNF in breast cancer. Cancer Cell *25*, 3–4. https://doi.org/10.1016/j.ccr.2013.12.017.

37. Wang, L., Zhao, Z., Meyer, M.B., Saha, S., Yu, M., Guo, A., Wisinski, K.B., Huang, W., Cai, W., Pike, J.W., et al. (2014). CARM1 methylates chromatin remodeling factor BAF155 to enhance tumor progression and metastasis. Cancer Cell *25*, 21–36. https://doi.org/10.1016/j.ccr.2013.12.007.

38. Neri, P., Barwick, B.G., Jung, D., Patton, J.C., Maity, R., Tagoug, I., Stein, C.K., Tilmont, R., Leblay, N., Ahn, S., et al. (2024). ETV4-Dependent Transcriptional Plasticity Maintains MYC Expression and Results in IMiD Resistance in Multiple Myeloma. Blood Cancer Discov. *5*, 56–73. https://doi.org/10.1158/2643-3230.BCD-23-0061.

39. Walker, B.A., Mavrommatis, K., Wardell, C.P., Ashby, T.C., Bauer, M., Davies, F.E., Rosenthal, A., Wang, H., Qu, P., Hoering, A., et al. (2018). Identification of novel mutational drivers reveals oncogene dependencies in multiple myeloma. Blood *132*, 587–597. https://doi.org/10.1182/blood-2018-03-840132.

40. Martello, M., Poletti, A., Borsi, E., Solli, V., Dozza, L., Barbato, S., Zamagni, E., Tacchetti, P., Pantani, L., Mancuso, K., et al. (2022). Clonal and subclonal TP53 molecular impairment is associated with prognosis and progression in multiple myeloma. Blood Cancer J. *12*, 15. https://doi.org/10.1038/s41408-022-00610-y.

41. Chin, M., Sive, J.I., Allen, C., Roddie, C., Chavda, S.J., Smith, D., Blombery, P., Jones, K., Ryland, G.L., Popat, R., et al. (2017). Prevalence and timing of TP53 mutations in del(17p) myeloma and effect on survival. Blood Cancer J. *7*, e610. https://doi.org/10.1038/bcj.2017.76.

42. Walker, B.A., Mavrommatis, K., Wardell, C.P., Ashby, T.C., Bauer, M., Davies, F., Rosenthal, A., Wang, H., Qu, P., Hoering, A., et al. (2019). A high-risk, Double-Hit, group of newly diagnosed myeloma identified by genomic analysis. Leukemia *33*, 159–170. https://doi.org/10.1038/s41375-018-0196-8.

43. Chou, T.C., and Talalay, P. (1984). Quantitative analysis of dose-effect relationships: the combined effects of multiple drugs or enzyme inhibitors. Adv. Enzym. Regul. *22*, 27–55. https://doi.org/10.1016/0065-2571(84)90007-4.

44. Herold, M.J., van den Brandt, J., Seibler, J., and Reichardt, H.M. (2008). Inducible and reversible gene silencing by stable integration of an shRNA-encoding lentivirus in transgenic rats. Proc. Natl. Acad. Sci. USA *105*, 18507–18512. https://doi.org/10.1073/pnas.0806213105.

45. Weisberg, E., Boulton, C., Kelly, L.M., Manley, P., Fabbro, D., Meyer, T., Gilliland, D.G., and Griffin, J.D. (2002). Inhibition of mutant FLT3 receptors in leukemia cells by the small molecule tyrosine kinase inhibitor PKC412. Cancer Cell *1*, 433–443. https://doi.org/10.1016/s1535-6108(02)00069-7.

46. Donovan, K.A., An, J., Nowak, R.P., Yuan, J.C., Fink, E.C., Berry, B.C., Ebert, B.L., and Fischer, E.S. (2018). Thalidomide promotes degradation of SALL4, a transcription factor implicated in Duane Radial Ray syndrome. Elife *7*, e38430. https://doi.org/10.7554/eLife.38430.

OMTON, Volume *33*

Supplemental information

Dual CARM1-and IKZF3-targeting: A novel approach to multiple myeloma therapy synergy between CARM1 inhibition and IMiDs

Wei Ni, Swati Garg, Basudev Chowdhury, Martin Sattler, Dana Sanchez, Chengcheng Meng, Taisei Akatsu, Katherine A. Donovan, Jun Qi, Michelle Y. Wang, Cara Ann Starnbach, Xiaoxi Liu, Maria Tarazona Guzman, Wei Pin Teh, Richard Stone, James D. Griffin, Sara Buhrlage, and Ellen Weisberg

Supplemental Materials and Methods

Table S1 Raw proteomics data for the 8-hour time point corresponding to 074 treatment of MOLT4 cells. Multiplexed quantitative proteomics experiment used for assessing protein abundance changes in response to degrader treatment.

Table S2 Raw proteomics data for the 24-hour time point corresponding to 074 treatment of MOLT4 cells. Multiplexed quantitative proteomics experiment used for assessing protein abundance changes in response to degrader treatment.

Table S3 Enzyme assay assessing 074 inhibition of CARM1.

Quantitative PCR (qPCR)

The following primers were used for qPCR.

Gene	Forward primer (5'->3')	Reverse primer (5'->3')	Ampicon (bp)
MYC	AATGAAAAGGCCCCCAAGGTAGTTATCC	GTCGTTTCCGCAACAAGTCCTCTTC	112
IRF4	CCCGGAAATCCCGTACCAAT	TTATGCTTGGCTCTGTGGGG	158

074 Chemical Synthesis

General Experimental and Materials:

All commercially available starting materials and solvents were purchased from Sigma Aldrich, Fisher Scientific, Oakwood Chemical, and Combi Blocks and were used without further purification. Deuterated solvents ($CDCl_3$, DMSO-d^6) were obtained from Cambridge Isotope Laboratories. Compound purification was performed by normal phase column chromatography using Teledyne Combi Flash chromatography system, and/or reversed phase chromatography using Shimadzu Nexera LC40 Series with Shim-Pack GIS 10 μm C18 30x250 mm column. Reaction monitoring and mass spectrometry data were obtained using Waters ACQUITY UPLC I-Class system. ^{1}H-NMR spectra were recorded at 298K using a Bruker ARX 500 (500 MHz) spectrometer.

General Information for Starting Materials:

All starting materials were purchased from commercial sources and used without further purification unless otherwise specified. Intermediates **1**, **3**, and **5** were synthesized according to literature procedure and the spectroscopic data were consistent with the reported values[1-3].

Supplemental Figures

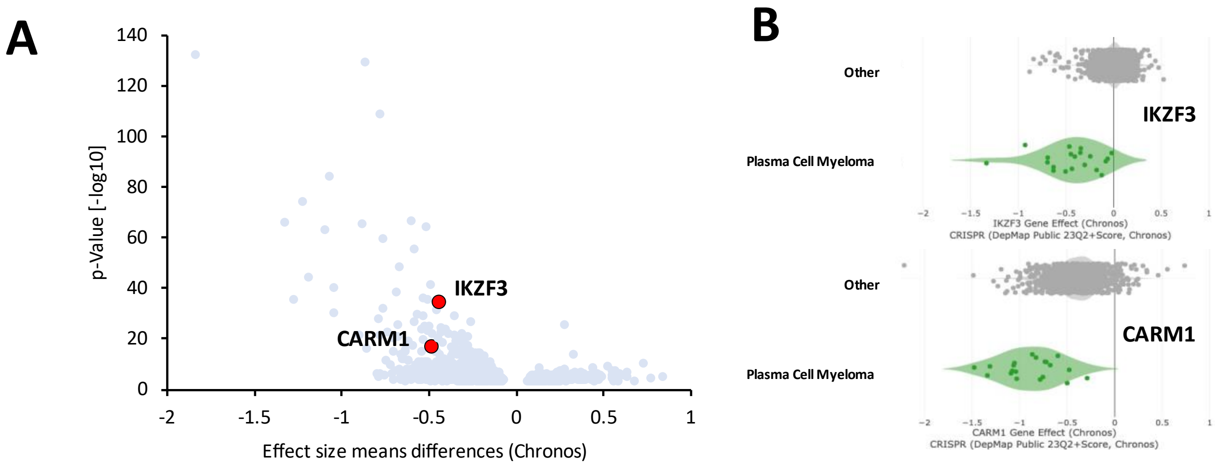

Figure S1. Genome-based CRISPR gene-editing screen identifies preferential dependencies in myeloma cell lines. A. Volcano plot of Chronos dependency scores and p-values within the DepMap dataset for myeloma identify IKZF3 and CARM1 as primary dependencies. B. Gene effect of IKZF3 and CARM1 targeting in 20 myeloma cell lines versus various other cancer cell lines tested within this screen. Data were retrieved from https://depmap.org/.

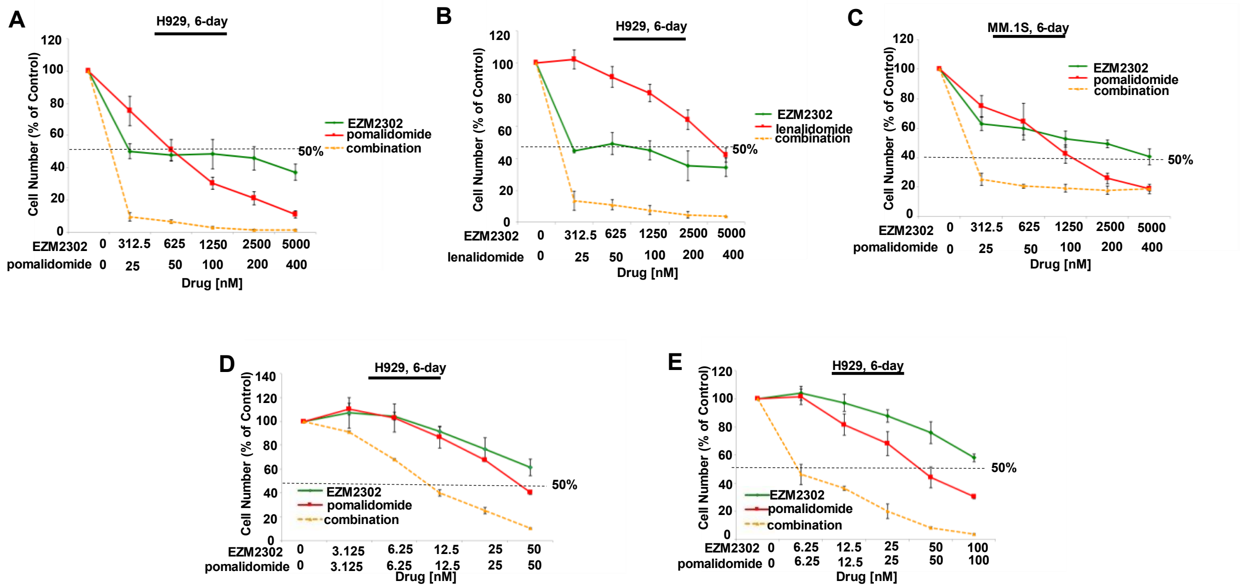

Figure S2. CARM1 inhibition potentiates the antiproliferative effects of pomalidomide against MM cells. (A) Proliferation assay: 6-day treatment of H929 cells with EZM2302, pomalidomide, or a combination. (B) Proliferation assay: 6-day treatment of H929 cells with EZM2302, lenalidomide, or a combination. (C) Proliferation assay: 6-day treatment of MM.1S cells with EZM2302, pomalidomide,or a combination. (D) Proliferation assay: 6-day treatment of H929 cells with EZM2302, pomalidomide, or a combination (concentration range 3.125-50 nM). (E) Proliferation assay: 6-day treatment of H929 cells with EZM2302, pomalidomide, or a combination (concentration range 6.25-100 nM).

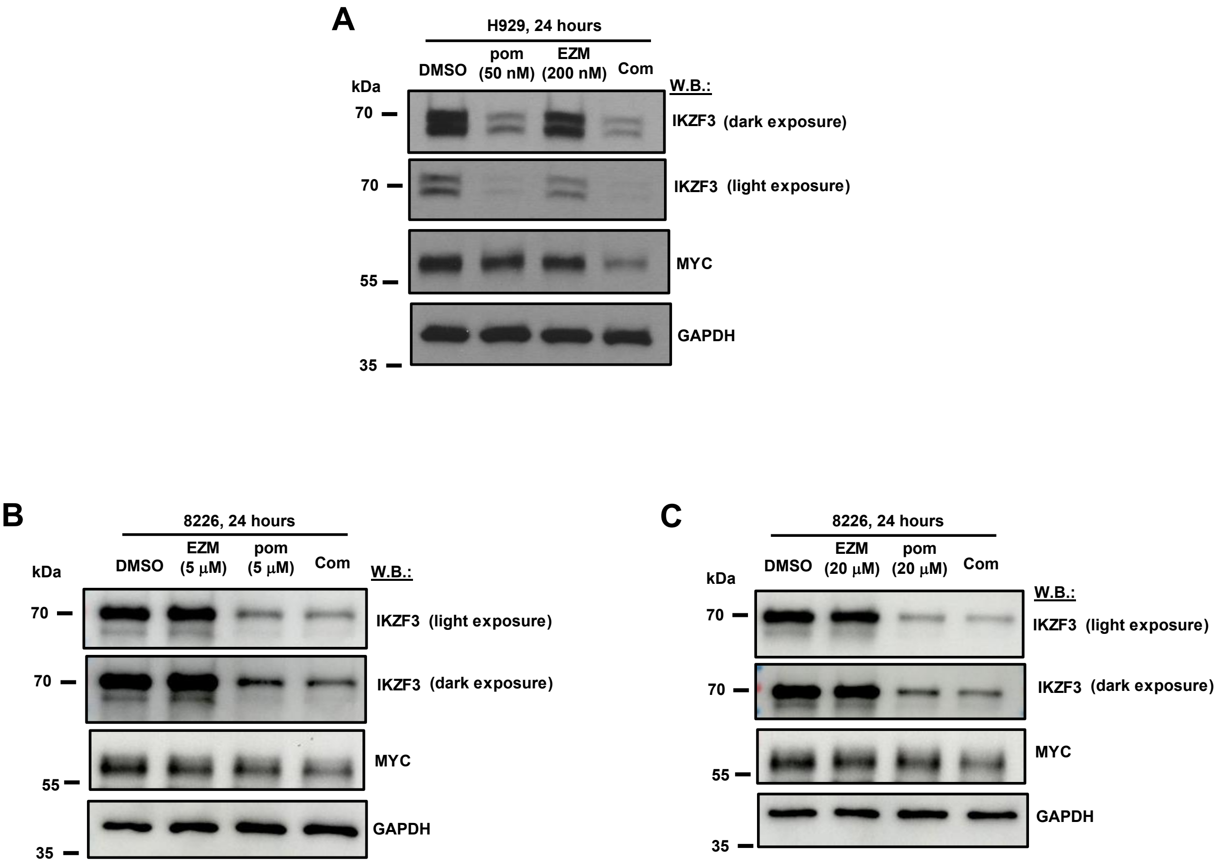

Figure S3 (A-C). Effects of CARM1 targeting and IMiD treatment on IKZF3 and MYC in MM cells. (A-C) Immunoblots: Effects of 24-hour treatment of H929 cells (A) or 8226 cells (B-C) with pomalidomide, EZM2302, or a combination on IKZF3 and MYC expression.

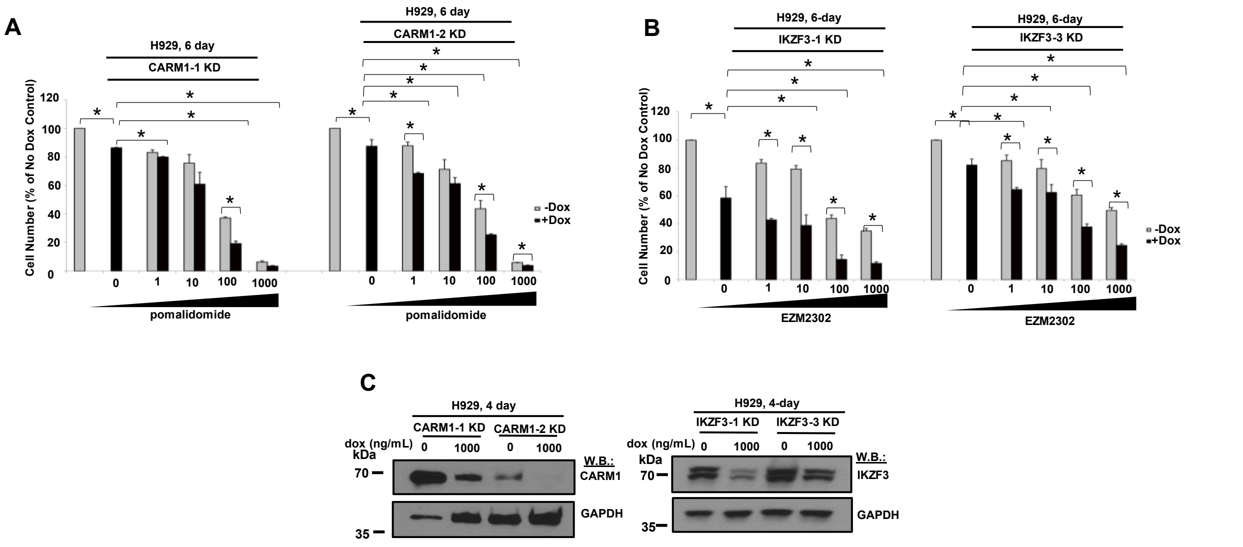

Figure S4. Contribution of CARM1 and IKZF3 to synergy between EZM2302 and pomalidomide. For doxycycline-inducible KD of *CARM1* and *IKZF3*, two individual clones were generated for each gene: ishCARM1-1 (CARM1-1 KD) and ishCARM1-2 for CARM1 (CARM1-2 KD), and ishIKZF3-1 (IKZF3-1 KD) and ishIKZF3-3 (IKZF3-3 KD) for IKZF3. (A) Effects of doxycycline-inducible CARM1 KD on potency of pomalidomide against MM cell growth. (B) Effects of doxycycline-inducible IKZF3 KD on potency of CARM1 inhibition against MM cell growth. (C) Immunoblots: (Left panel) Assessment of CARM1 KD efficiency in doxycycline-treated H929 CARM1 KD cells following a 4-day treatment. (Right panel): Immunoblots: Assessment of IKZF3 KD efficiency in doxycycline-treated H929 IKZF3 KD cells following a 4-day treatment.

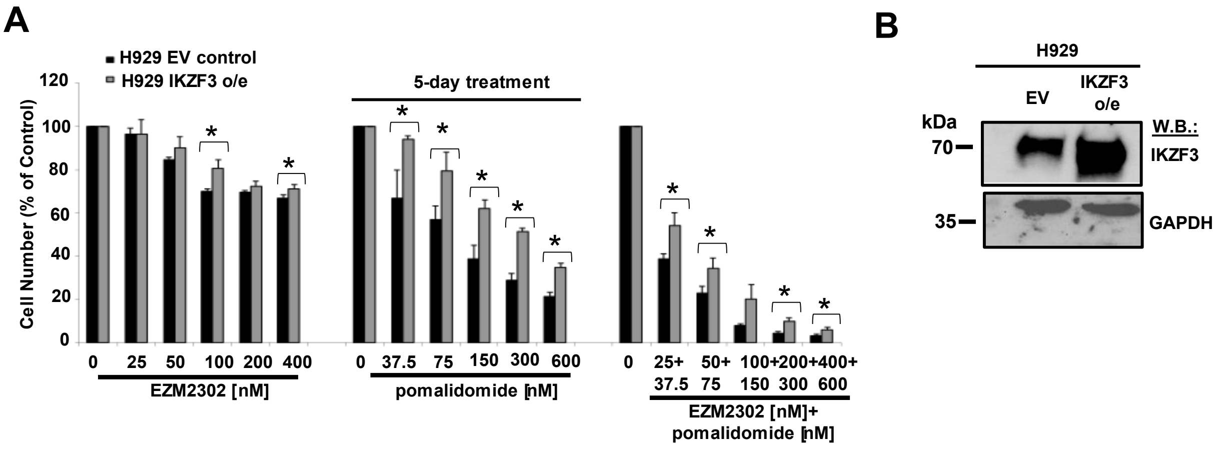

Figure S5. Contribution of IKZF3 to synergy between EZM2302 and pomalidomide. (A) Effects of IKZF3 overexpression (IKZF3 o/e) in MM cells on EZM2302, pomalidomide, or a combination. (B) Immunoblots: Assessment of IKZF3 levels in H929 cells engineered to overexpress IKZF3.

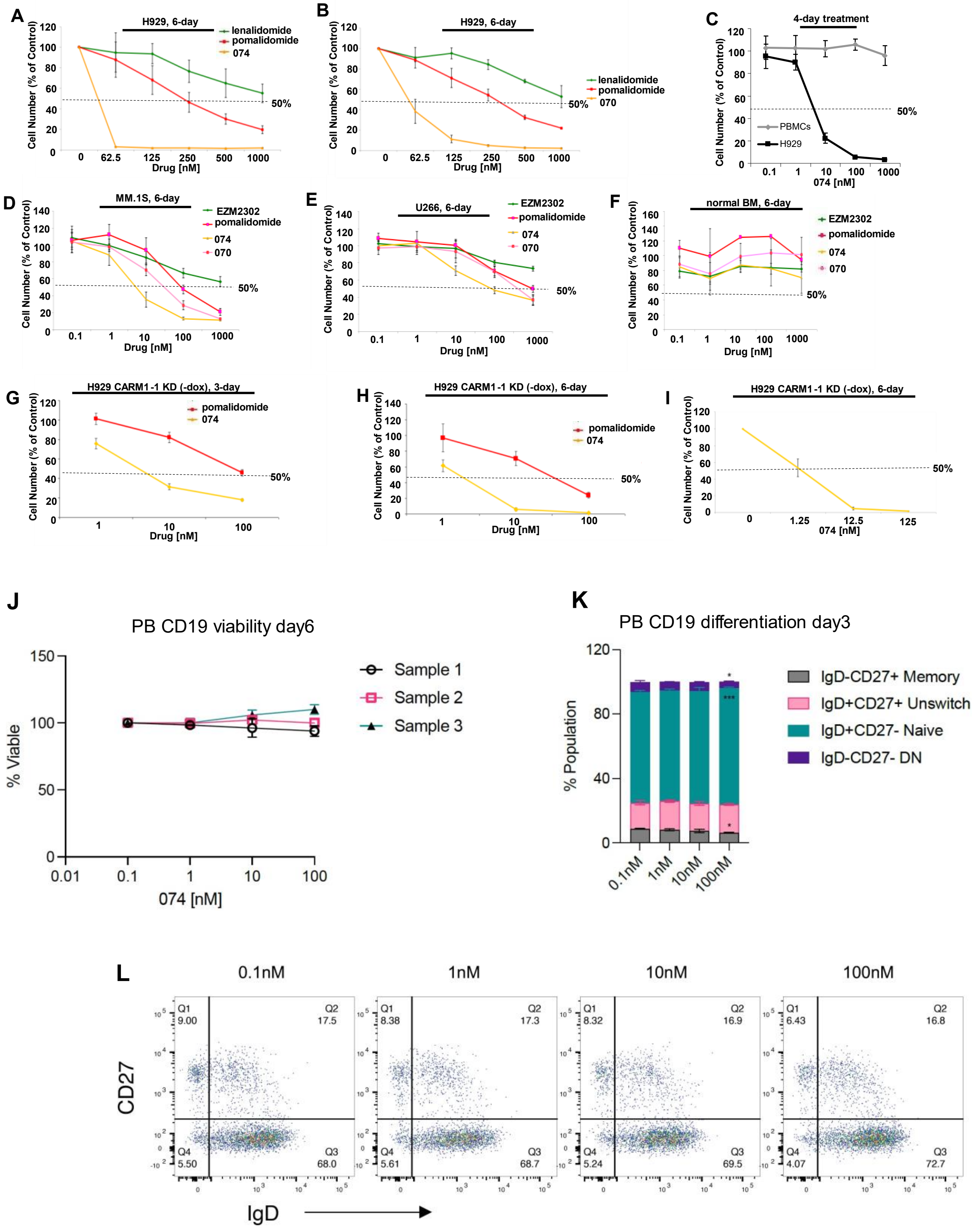
A
H929, 6-day
lenalidomide
pomalidomide
074
Cell Number (% of Control)
50%
Drug [nM]
B
H929, 6-day
C
4-day treatment
PBMCs
H929
074 [nM]
D
MM.1S, 6-day
EZM2302
070
E
U266, 6-day
F
normal BM, 6-day
G
H929 CARM1-1 KD (-dox), 3-day
H
H929 CARM1-1 KD (-dox), 6-day
I
H929 CARM1-1 KD (-dox), 6-day
J
PB CD19 viability day6
% Viable
Sample 1
Sample 2
Sample 3
074 [nM]
K
PB CD19 differentiation day3
% Population
IgD-CD27+ Memory
IgD+CD27+ Unswitch
IgD+CD27- Naive
IgD-CD27- DN
L
0.1nM
1nM
10nM
100nM
CD27
IgD

Figure S6. 070 and 074 more potently inhibit the proliferation of MM cell lines than EZM2302 or IMiDs. (A-B) Proliferation studies: H929 treated with lenalidomide, pomalidomide, 074 or 070 for 6 days. (C) Proliferation assay: 4-day treatment of normal PBMCs versus H929 cells with 074. (D) Proliferation studies: MM.1S treated with EZM2302, pomalidomide, 074 or 070 for 6 days. (E) Proliferation studies: U266 treated with EZM2302, pomalidomide, 074 or 070 for 6 days. (F) Proliferation studies: Normal bone marrow treated with EZM2302, pomalidomide, 074 or 070 for 6 days. (G-H) Proliferation studies: 074 versus pomalidomide treatment of dox-inducible H929 CARM1-1 KD cells in the absence of doxycycline for 3 days (G) and 6 days (H). (I) H929 CARM1-1 KD cells treated with 074 in the absence of doxycycline for 6 days. (J) CD19+ B cells enriched from healthy donor peripheral blood were treated with increasing concentrations of 074, and on day 6 cells were stained with DAPI for HTS acquisition on flow cytometer. The data plot shows cell viability (mean and SD) from a minimum of 5 replicates with each concentration normalized to the smallest concentration of 0.1nM. (K) CD19+ cells from one sample were treated in triplicates and subjected to flow cytometry on day 3. The statistical plot shows mean and SD, and comparison from 0.1nM in two-way ANOVA using Dunnett's multiple comparisons test; asterisk shows $p<0.01$. (L) A representative flow cytometry plot showing distribution of naïve (IgD+CD27-), unswitched (IgD+CD27+), memory (IgD-CD27+) and double negative cells on day 3 post-treatment.

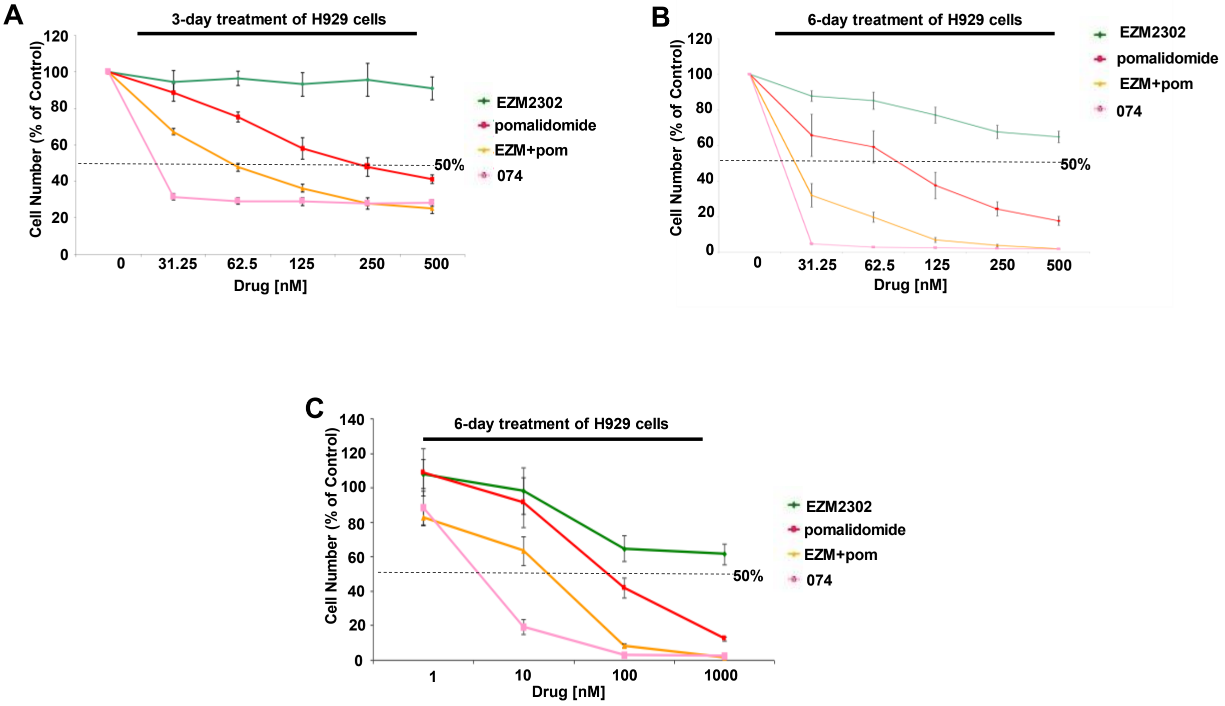

Figure S7. Comparison of the potency of 074 versus the combination of CARM1 inhibition+pomalidomide. (A-B) Proliferation studies: H929 cells treated with EZM2302, pomalidomide, EZM2302+pomalidomide, or 074 for 3 days (A) or 6 days (B) (concentration range 31.25-500 nM). (C) Proliferation studies: H929 cells treated with EZM2302, pomalidomide, EZM2302+pomalidomide, or 074 for 6 days (concentration range 1-1000 nM).

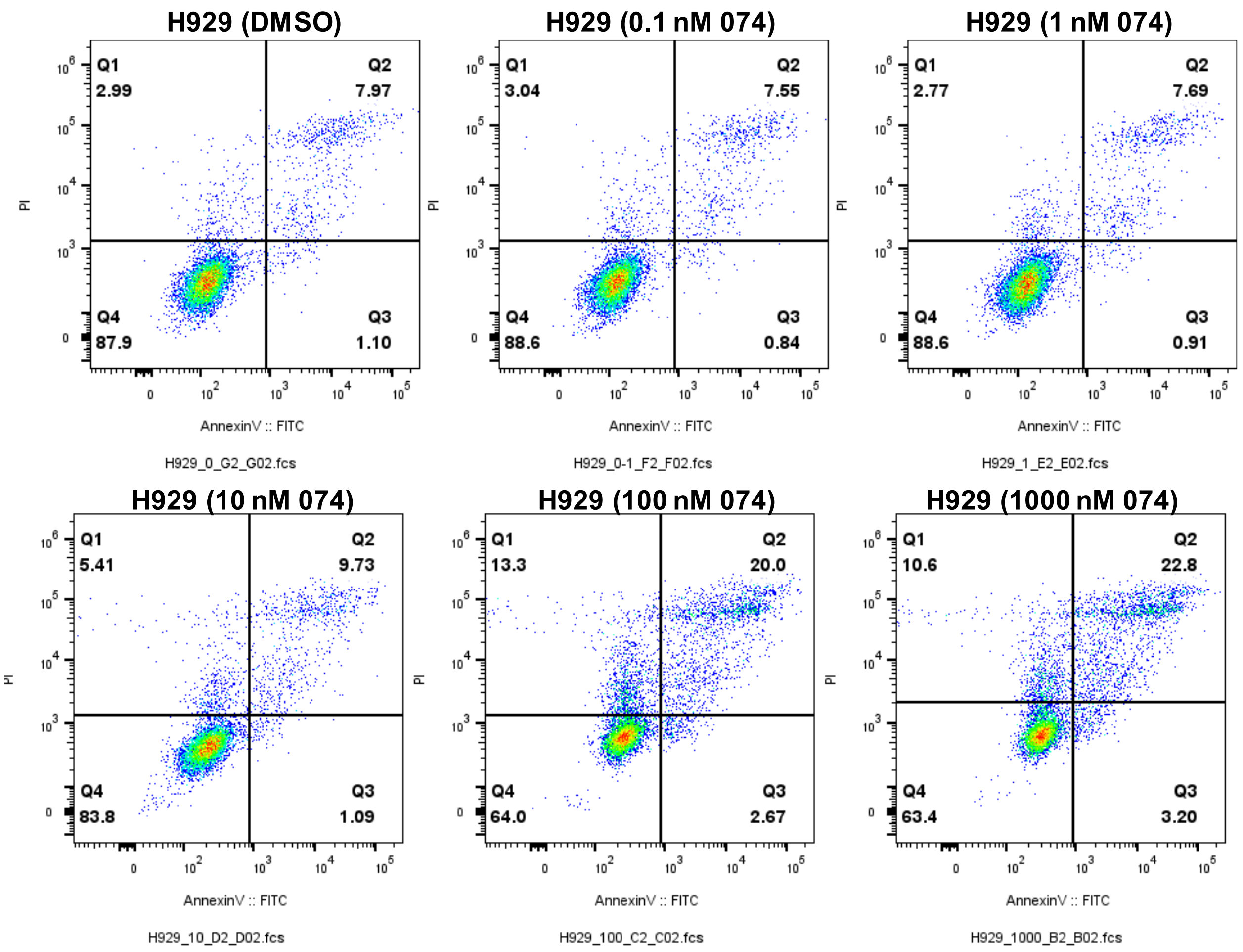

Figure S8. 074 induces late-stage apoptosis and necrosis in a concentration-dependent manner. 4-day 074 treatment of H929 cells followed by Annexin/PI staining as a measure of apoptosis.

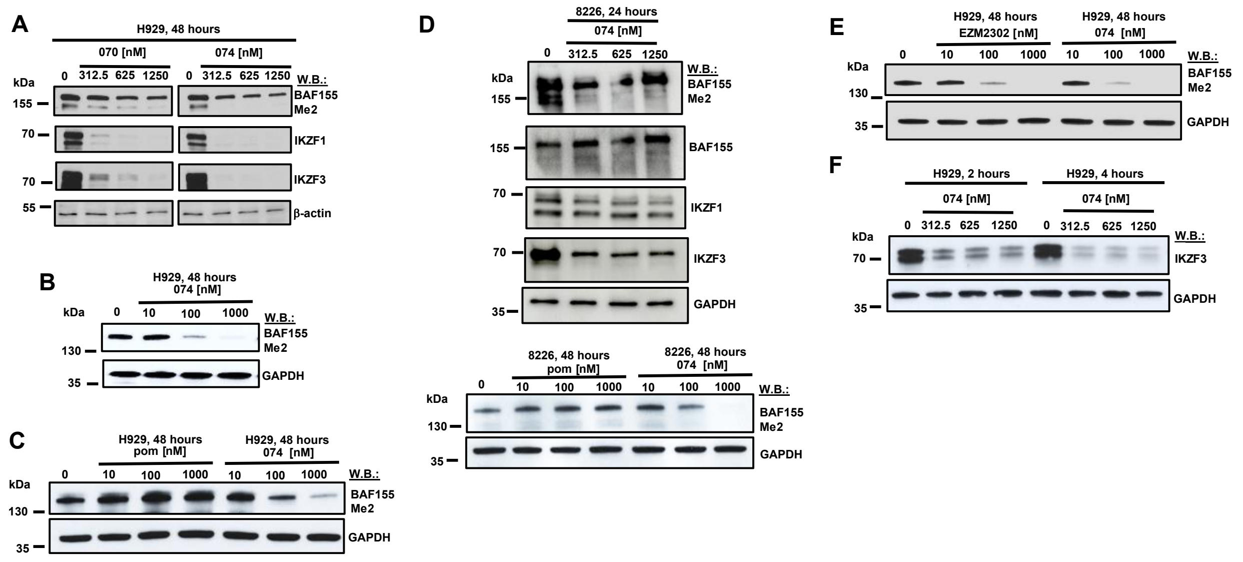

Figure S9 (A-F). Effects of 070 and 074 on BAF155 methylation and expression of IKZF1, IKZF3, MYC and IRF4. (A) Immunoblots: Effects of 070 and 074 on expression of methylated BAF155, IKZF1, and IKZF3 following 48-hour treatment of H929 cells. (B) Immunoblots: Effects of 074 on expression of methylated BAF155 following 48-hour treatment of H929 cells. (C) Immunoblots: Comparison of effects of pomalidomide and 074 on methylation of BAF155 in H929 cells. (D) Effects of 074 on expression of methylated BAF155, IKZF1, and IKZF3 following 24 hours of treatment of 8226 cells (lower panel) and effects of pomalidomide versus 074 on methylated BAF155 following 48 hours of treatment of 8226 cells (right panel). (E) Comparison of effects of EZM2302 and 074 on methylation of BAF155 in H929 cells following 48 hours of treatment. (F) Immunoblots: Effects of 074 on IKZF3 expression following 2-hour and 4-hour treatment of H929 cells.

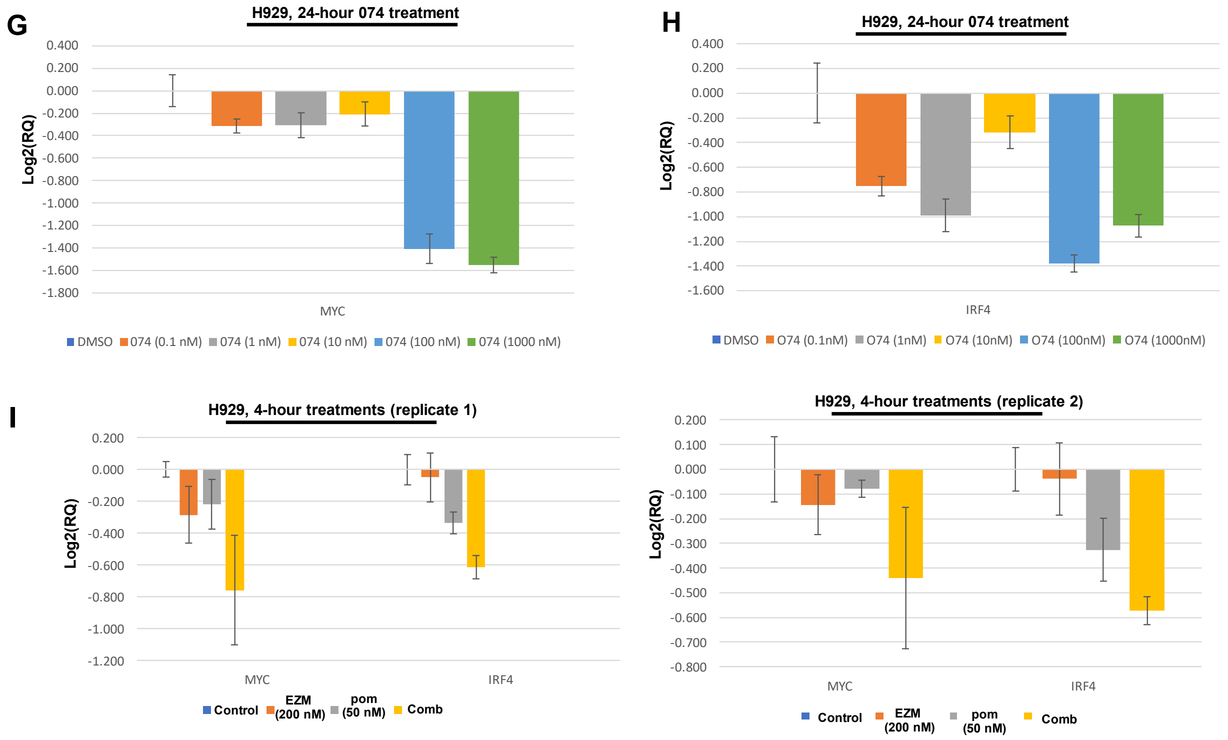

Figure S9 (G-I). Effects of CARM1 targeting+IMiD treatment or 074 on IKZF3, MYC and IRF4 in MM cells. (G) qPCR investigation of effects of 074 on transcription of MYC. (H) qPCR investigation of effects of 074 on transcription of IRF4. (I) qPCR investigation of effects of EZM2302 (200 nM), pomalidomide (50 nM), or the combination following a 4-hour treatment on MYC and IRF4 transcription in H929 cells.

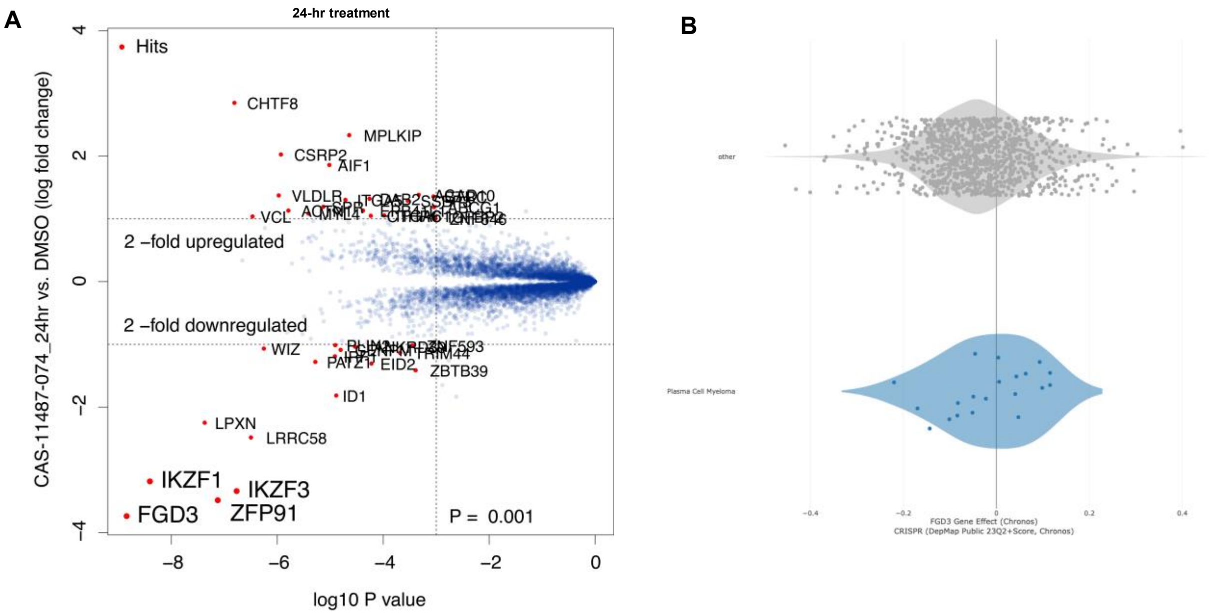

Figure S10. Proteomics identification of protein targets of 074. (A) Proteomics analysis for 074. 24-hour time point. (B) Gene effect of FGD3 targeting in 20 myeloma cell lines versus various other cancer cell lines from different lineages tested within this CRISPR screen. Data were retrieved from https://depmap.org/.

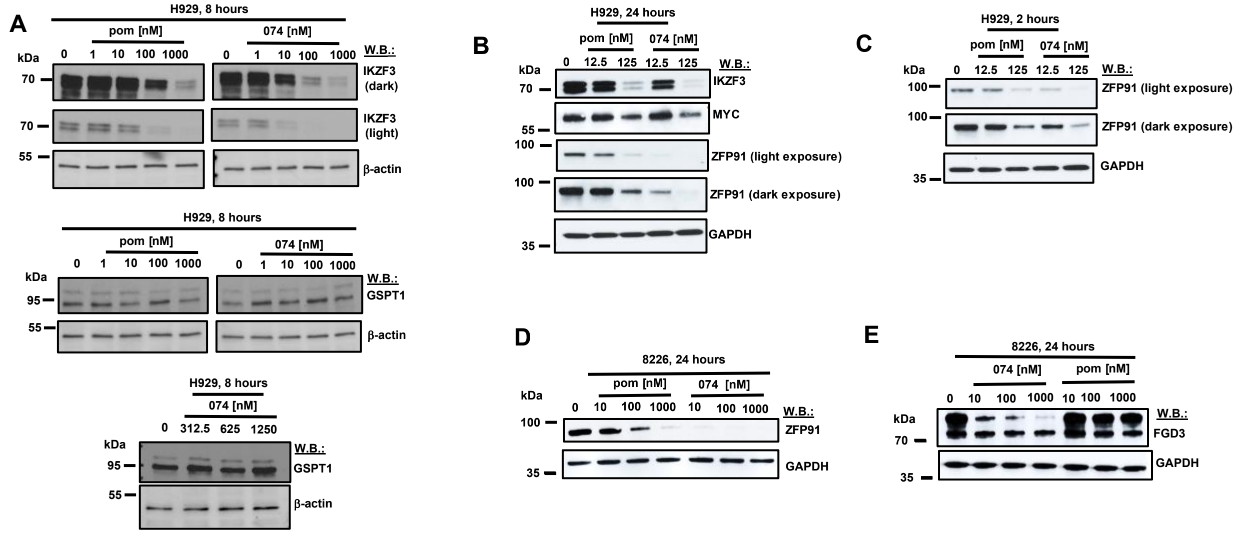

Figure S11. Comparison of effects of pomalidomide and 074 on IKZF1, IKZF3, GSTP1, and ZFP91 protein expression in H929 cells. (A) Effects of pomalidomide or 074 on expression of IKZF3 and GSPT1 following 8-hour treatment of H929 cells. (B) Effects of pomalidomide or 074 on expression of IKZF3, MYC, and ZFP91 following a 24-hour treatment of H929 cells. (C) Effects of pomalidomide or 074 on expression of ZFP91 following 2-hour treatment of H929 cells. (D-E) Effects of pomalidomide or 074 on expression of ZFP91 (D) or FGD3 (E) following a 24-hour treatment of 8226 cells.

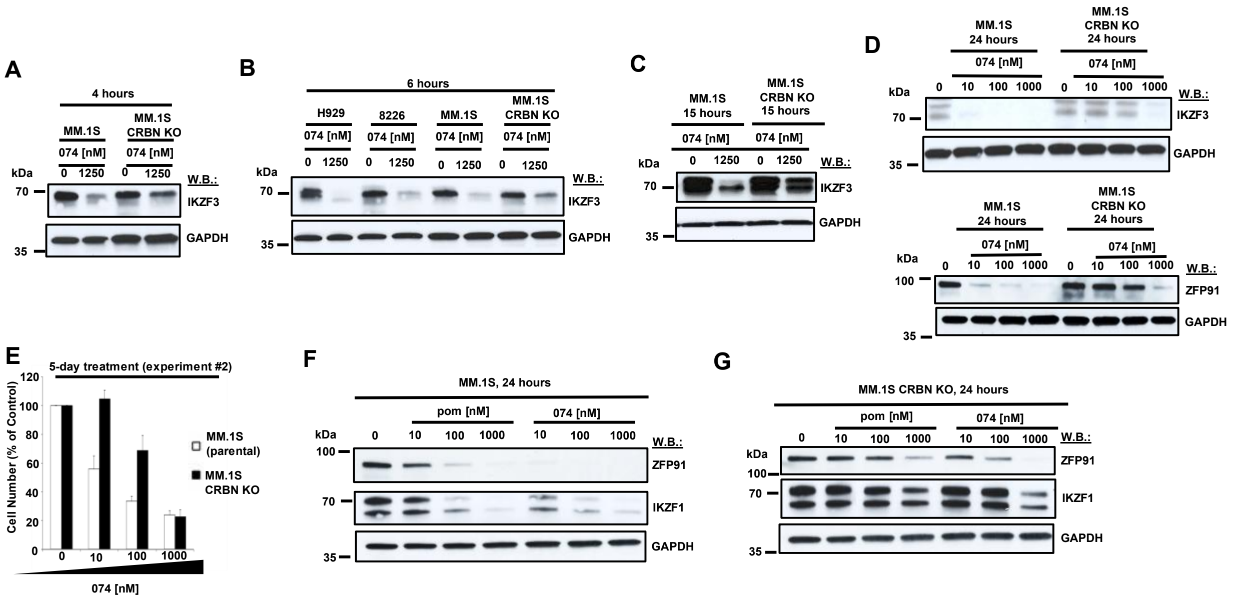

Figure S12. Effects of 074 treatment of MM.1S and MM.1S CRBN KO on IKZF3, CARM1, and ZFP91 protein levels. (A-B) Effects of 074 on IKZF3 protein levels in MM.1S and MM.1S CRBN KO cells following 4 hours (A) and 6 hours (B). In (B), H929 and 8226 cells are included as controls for comparison. (C) Effects of 074 on IKZF3 protein levels in MM.1S and MM.1S CRBN KO cells following 15 hours of treatment. (D) Effects of 074 on IKZF3 and ZFP91 protein levels in MM.1S and MM.1S CRBN KO cells following 24 hours of treatment (E) Proliferation study: 5-day 074 treatment of parental MM.1S cells and MM.1S CRBN KO cells. . (F) Immunoblots: Comparison of effects of pomalidomide and 074 on ZFP91 and IKZF1 protein expression following 24 hours of treatment of MM.1S cells. (G) Immunoblots: Comparison of effects of pomalidomide and 074 on ZFP91 and IKZF1 protein expression following 24 hours of treatment of MM.1S CRBN KO cells.

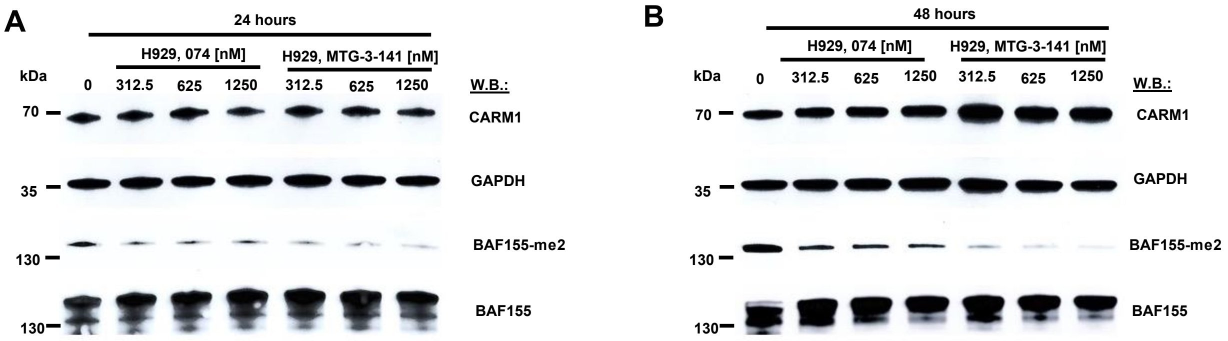

Figure S13. Effects of 074 or MTG-3-141 treatment of H929 cells on CARM1, meBAF155 and total BAF155. (A-B) Immunoblots: Effects of 24- and 48-hour 074 or MTG-3-141 treatment of H929 on levels of CARM1, methylated BAF155 and total BAF155.

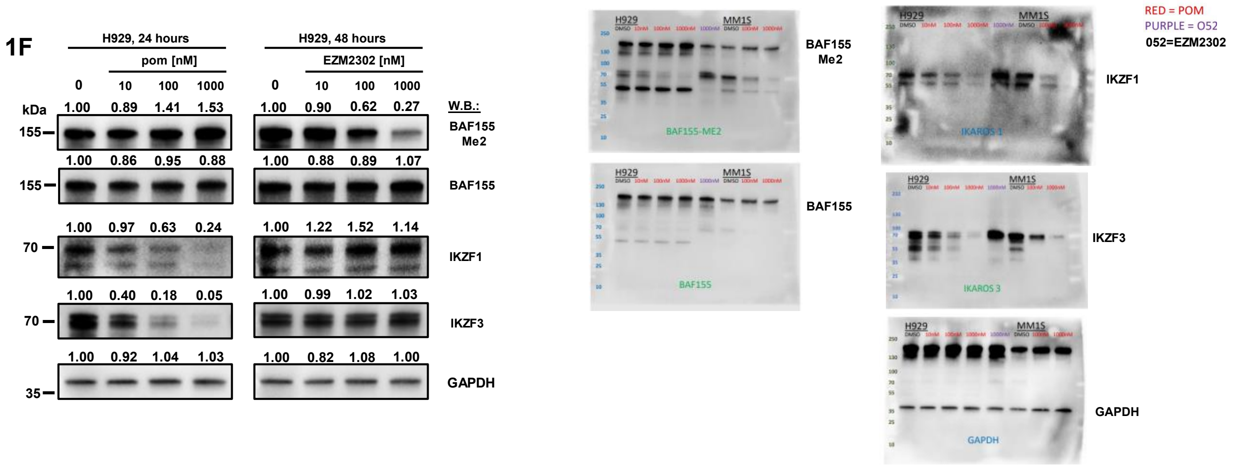

Figure S14 (part 1). Uncut gels/densitometry for Figure 1F (left panel).

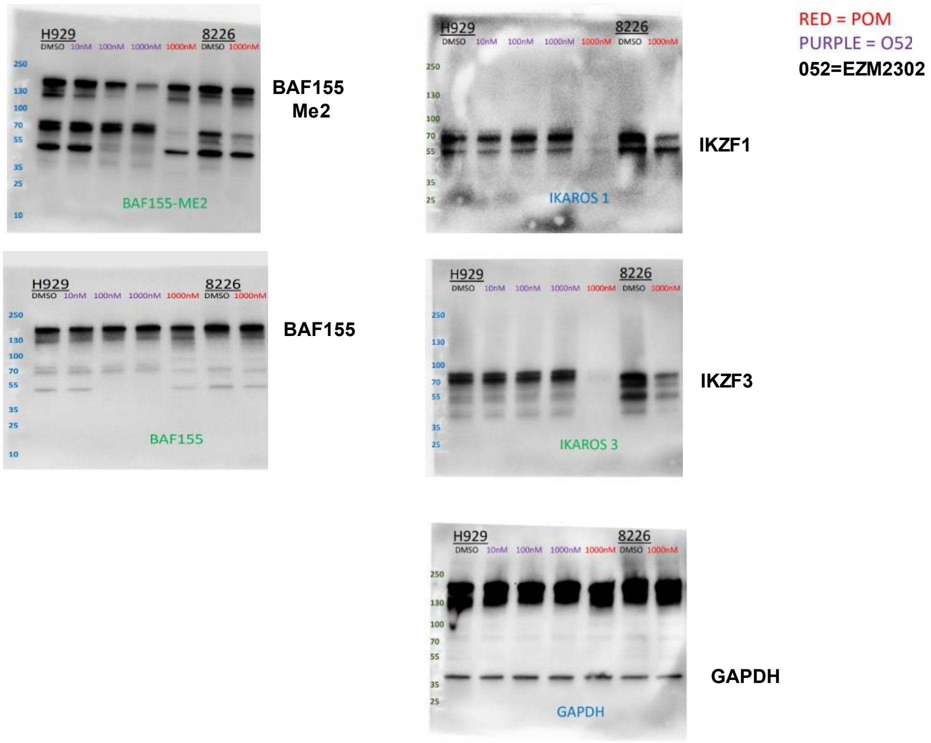

Figure S14 (part 2). Uncut gels/densitometry for Figure 1F (right panel).

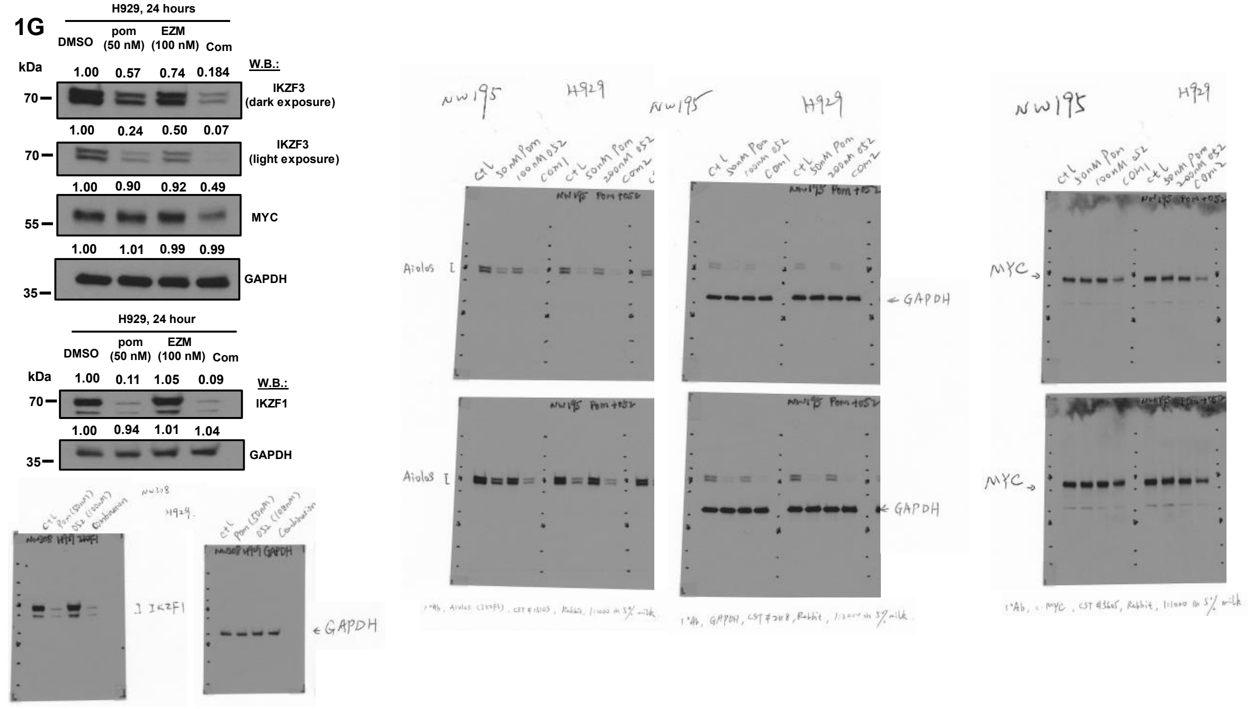

Figure S15. Uncut gels/densitometry for Figure 1G.

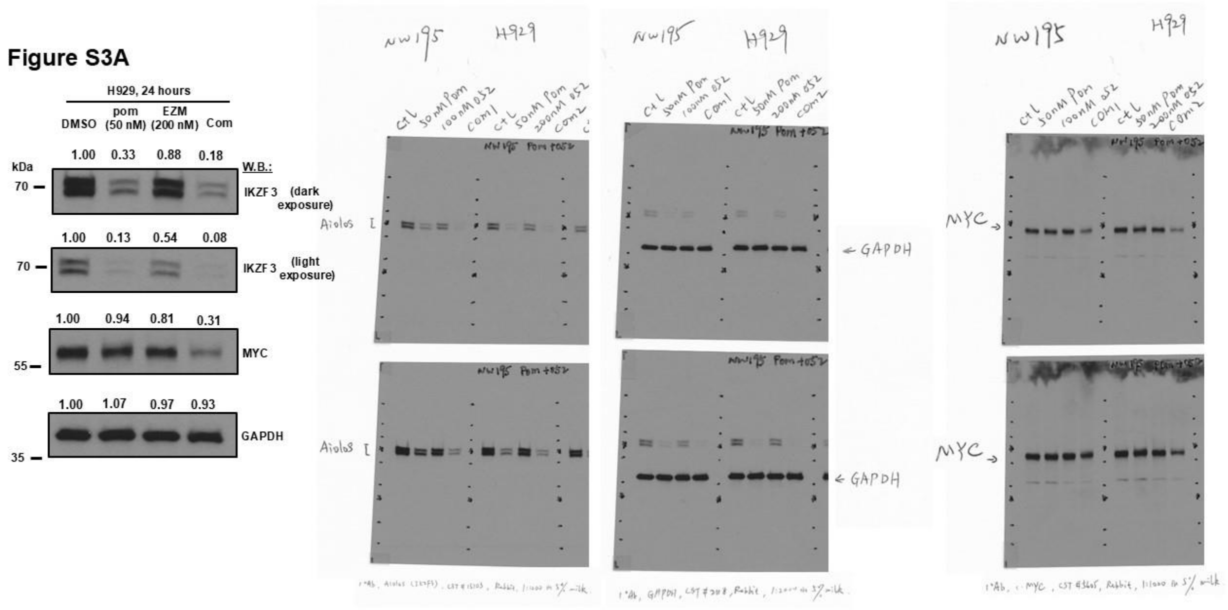

Figure S16 (part 1). Uncut gels/densitometry for Figure S3.

Figure S3B

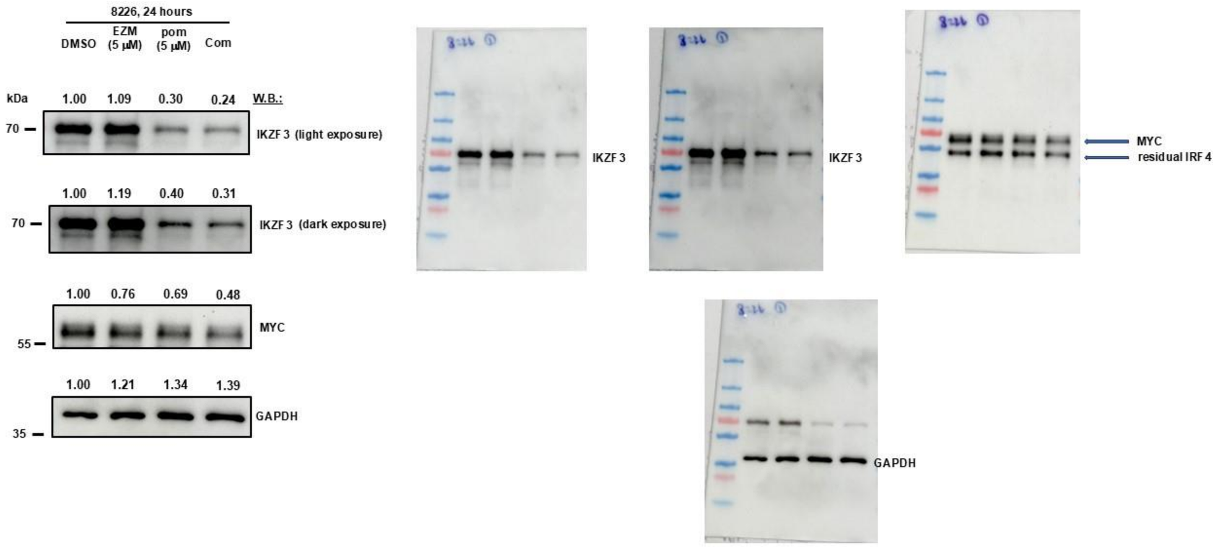

Figure S16 (part 2). Uncut gels/densitometry for Figure S3.

Figure S3C

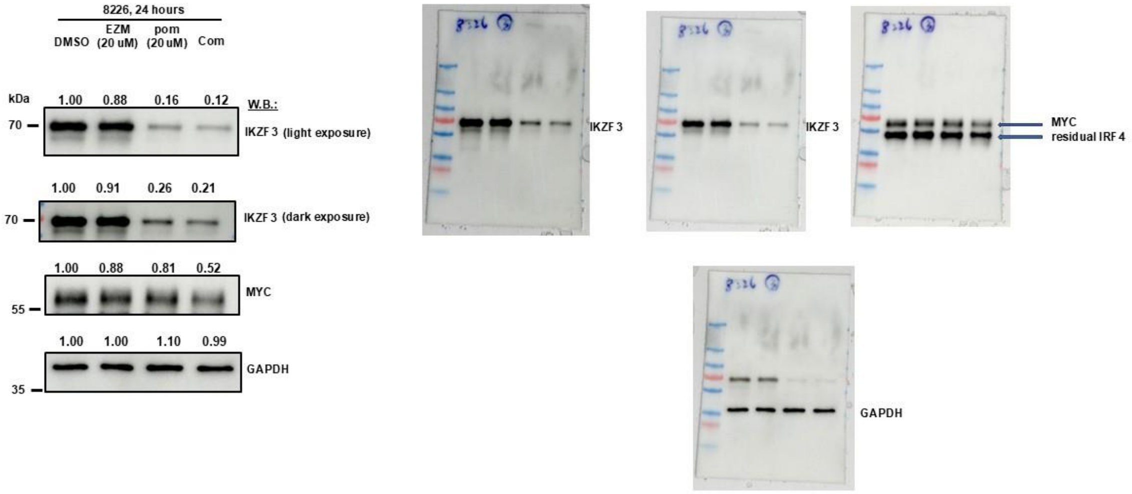

Figure S16 (part 3). Uncut gels/densitometry for Figure S3.

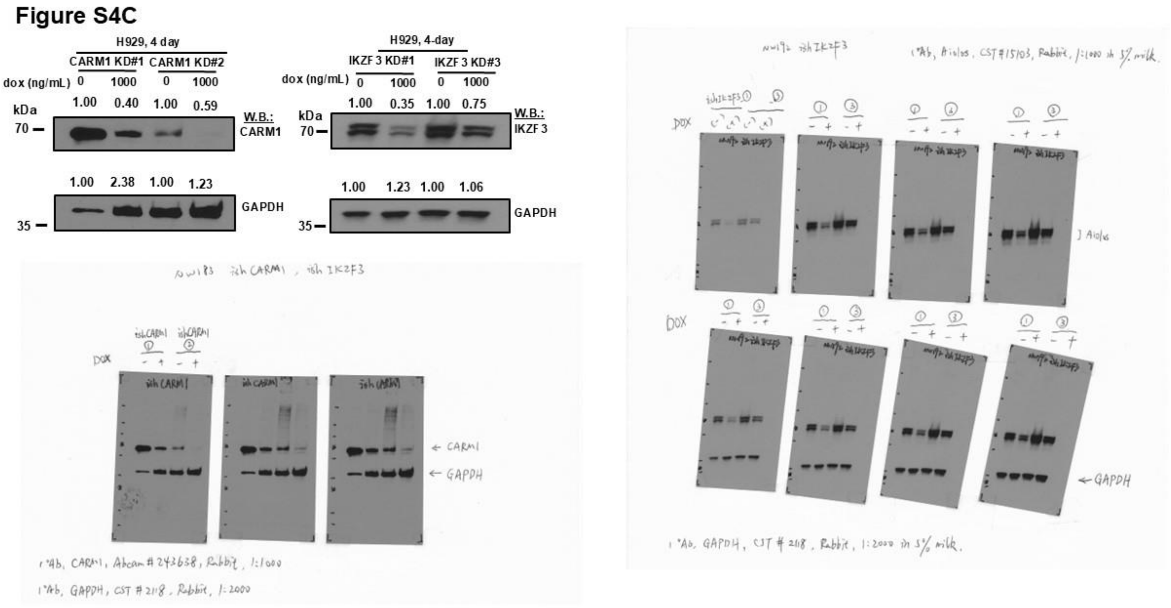

Figure S17. Uncut gels/densitometry for Figure S4C.

Figure S5B

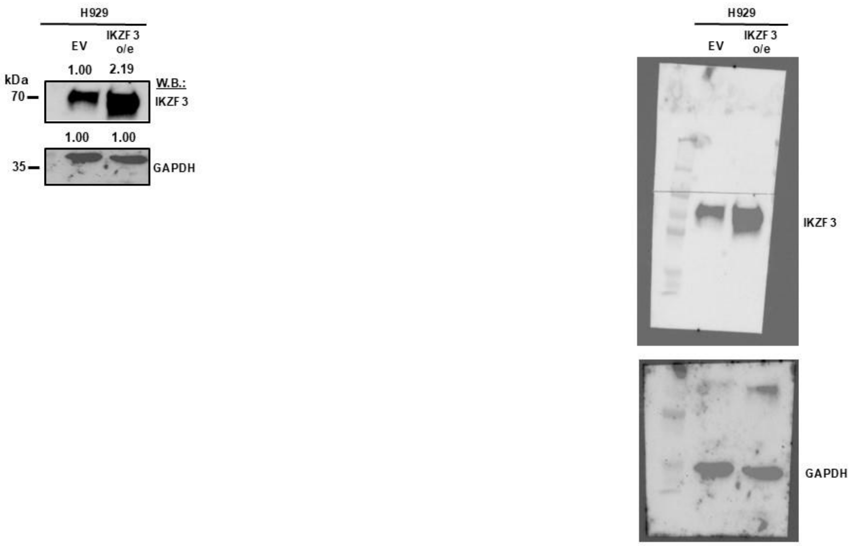

Figure S18. Uncut gels/densitometry for Figure S5B.

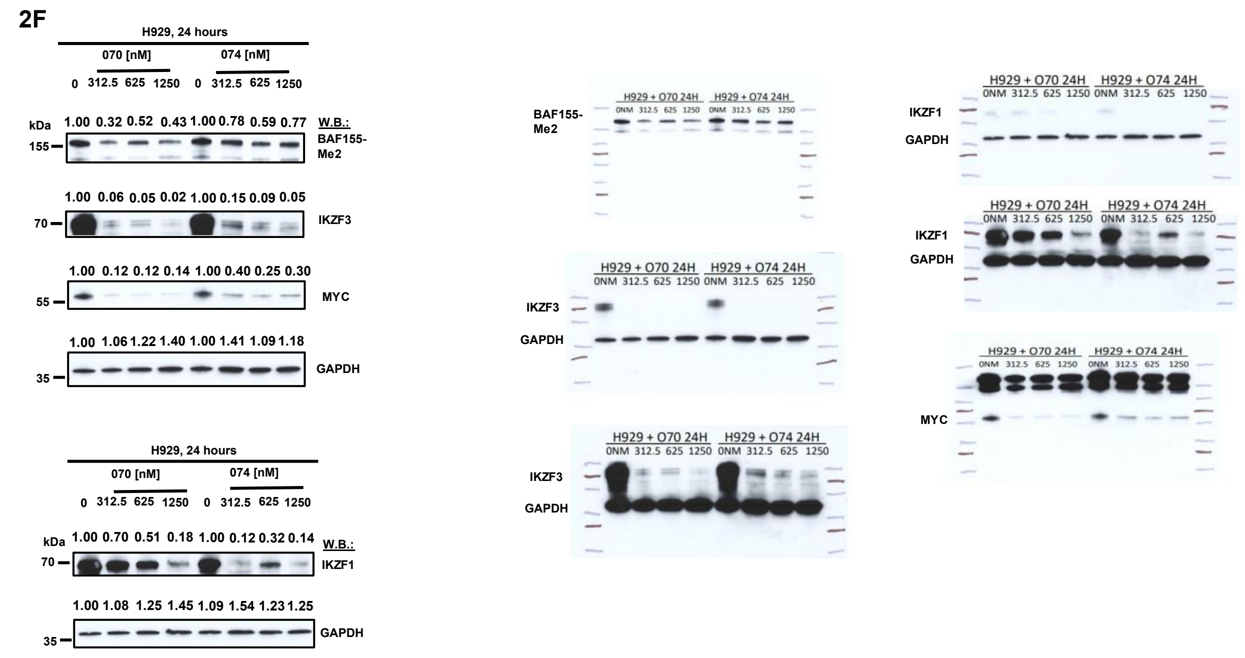

Figure S19 (part 1). Uncut gels/densitometry for Figure 2F.

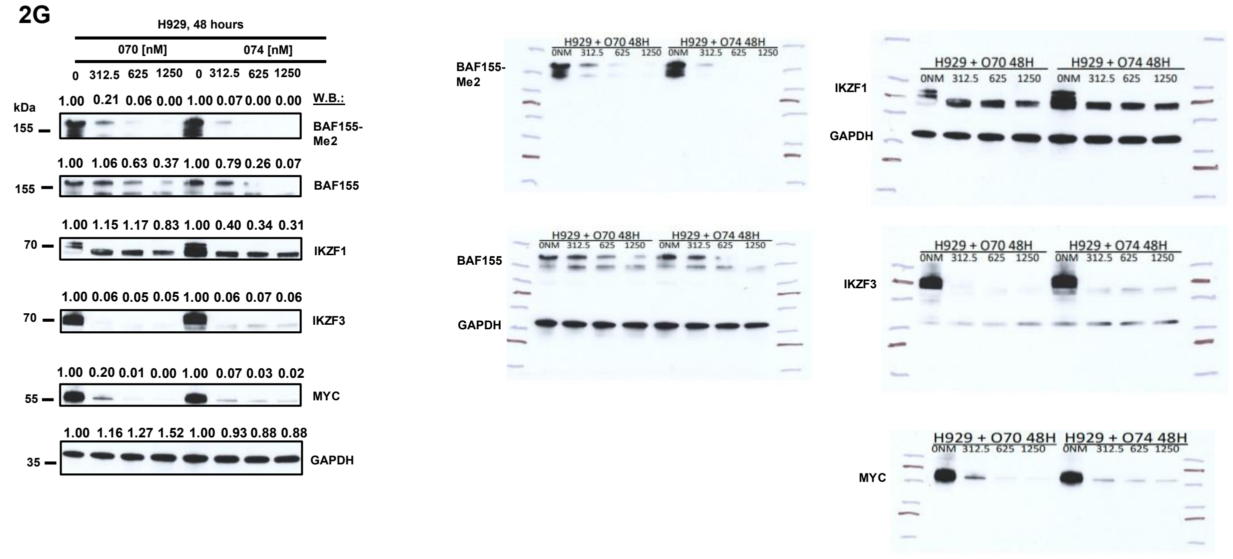

Figure S19 (part 2). Uncut gels/densitometry for Figure 2G.

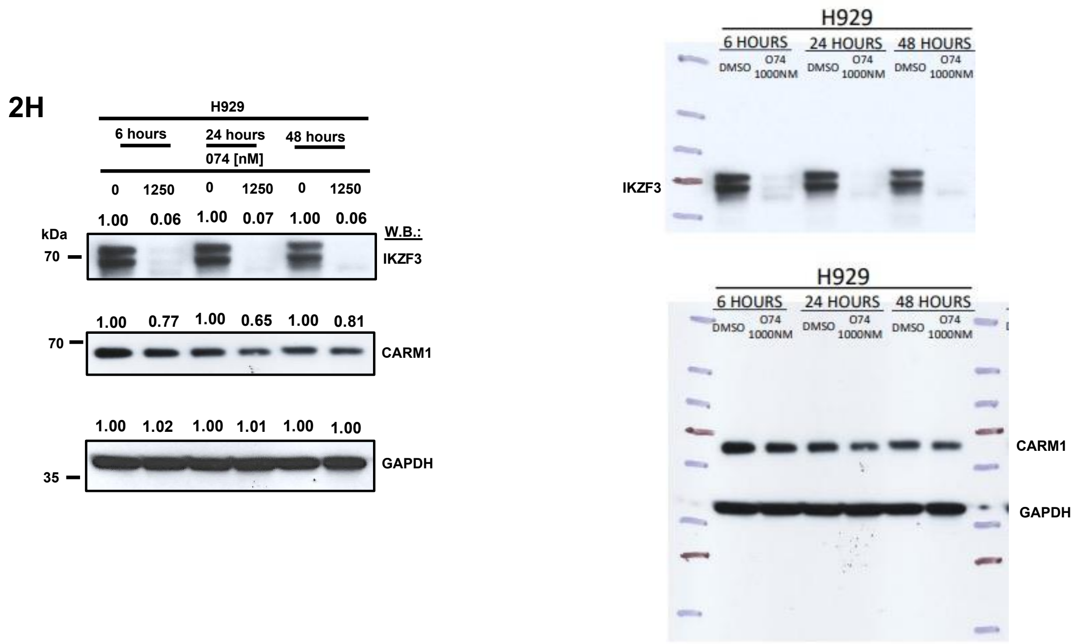

Figure S19 (part 3). Uncut gels/densitometry for Figure 2H.

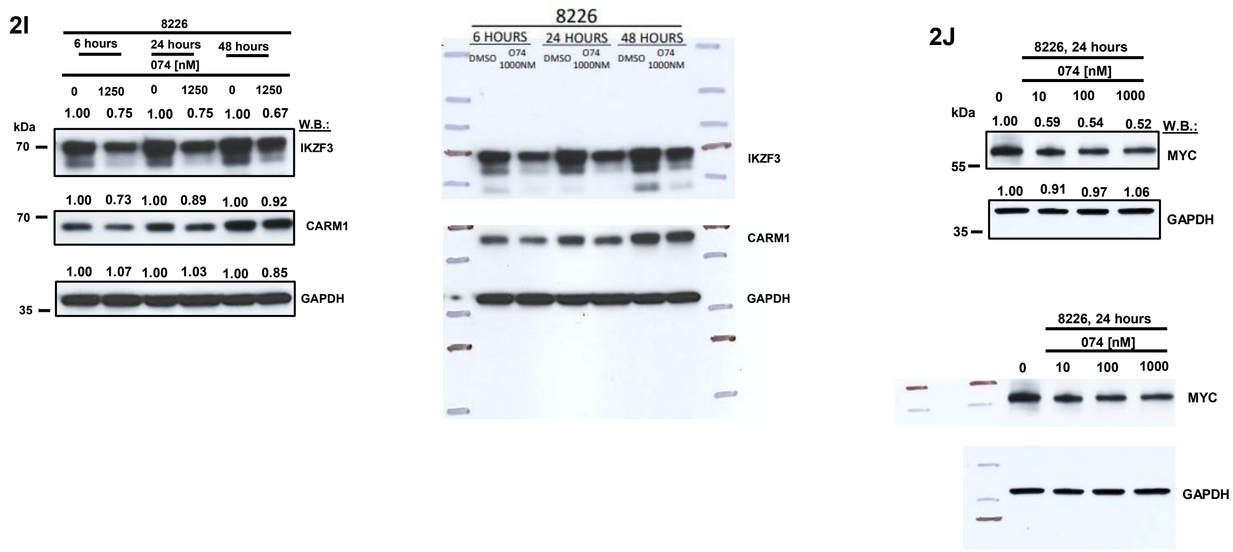

Figure S19 (part 4). Uncut gels/densitometry for Figure 2I and J.

Figure S8A

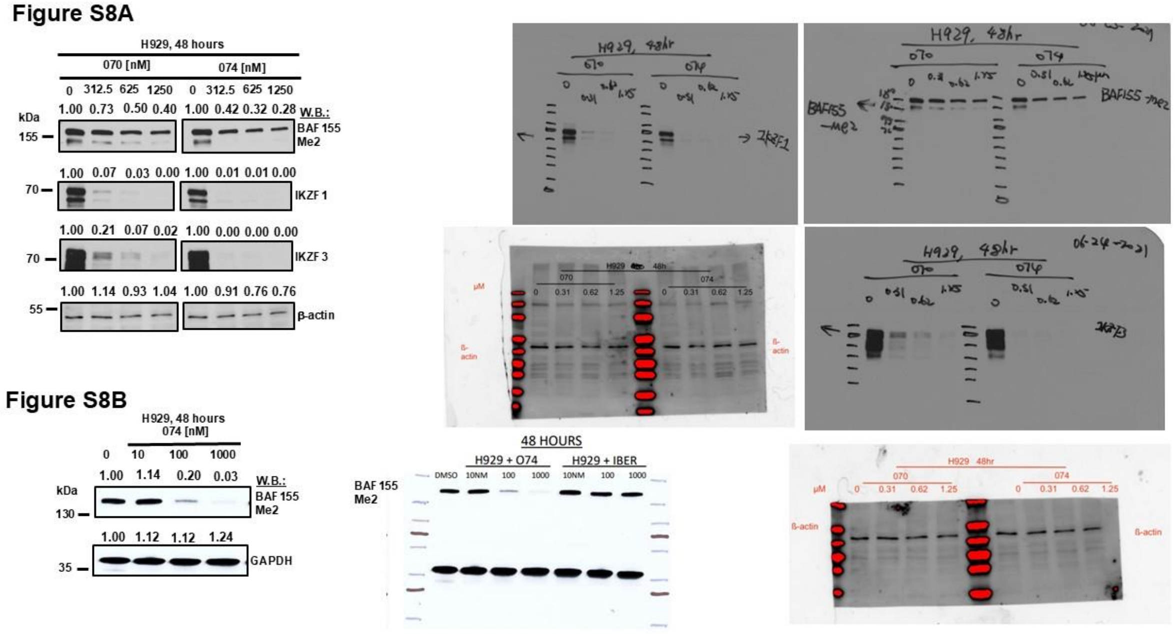

Figure S20 (part 1). Uncut gels/densitometry for Figure S8A and B.

Figure S8C

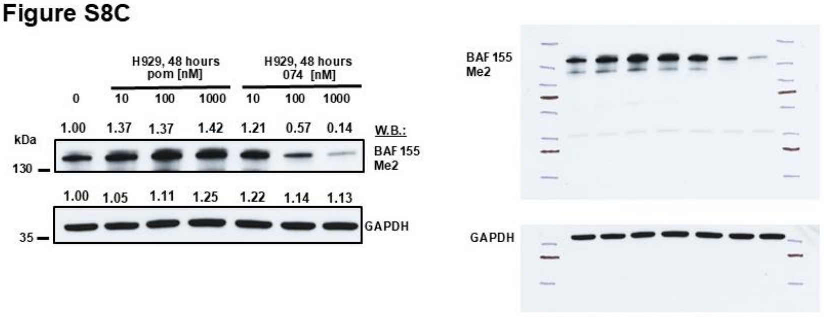

Figure S20 (part 2). Uncut gels/densitometry for Figure S8C.

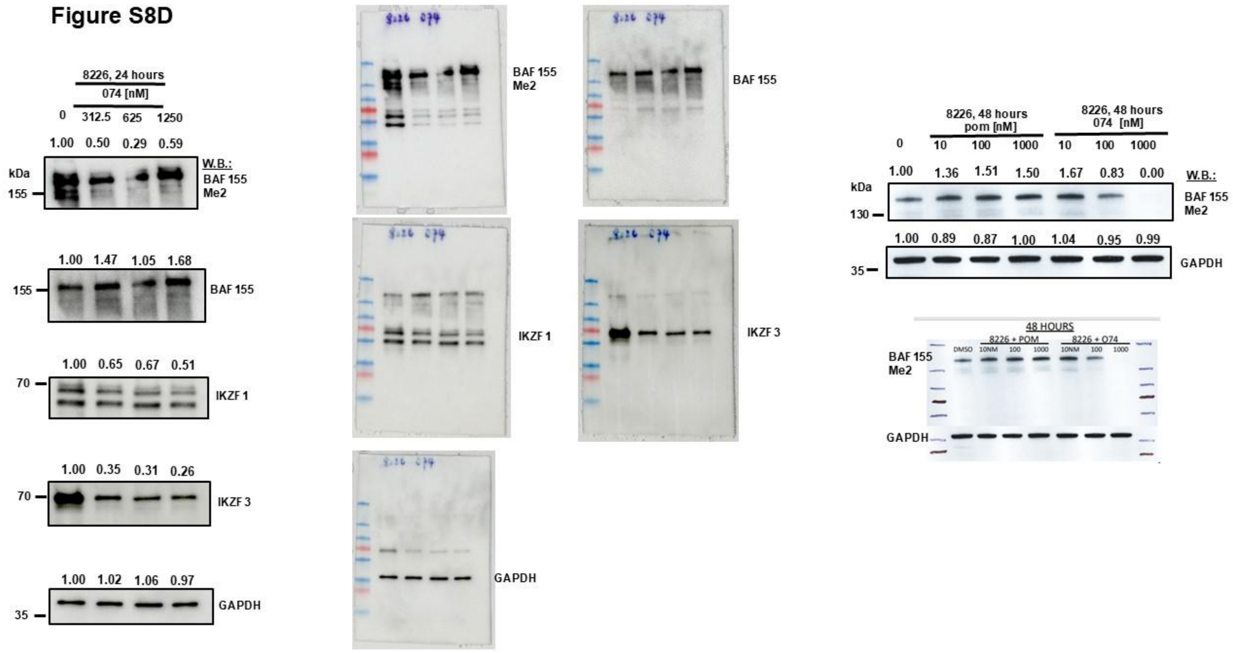

Figure S20 (part 3). Uncut gels/densitometry for Figure S8D.

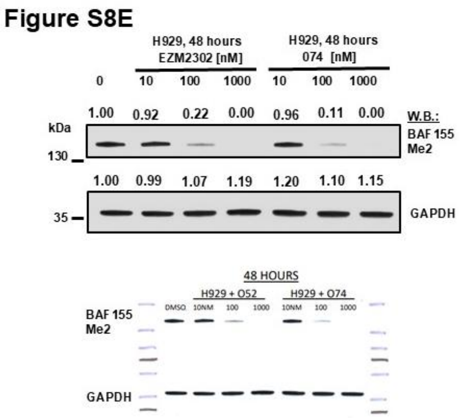

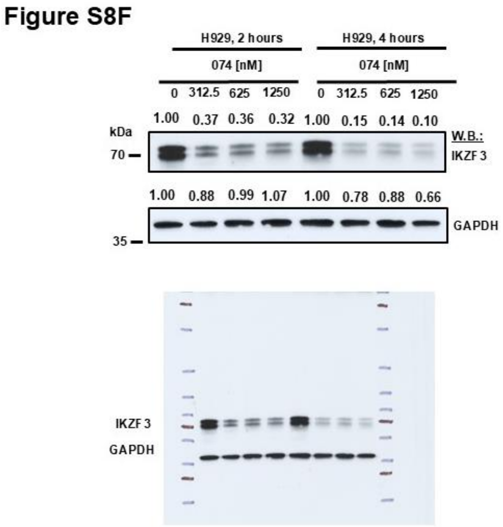

Figure S20 (part 4). Uncut gels/densitometry for Figure S8E and F.

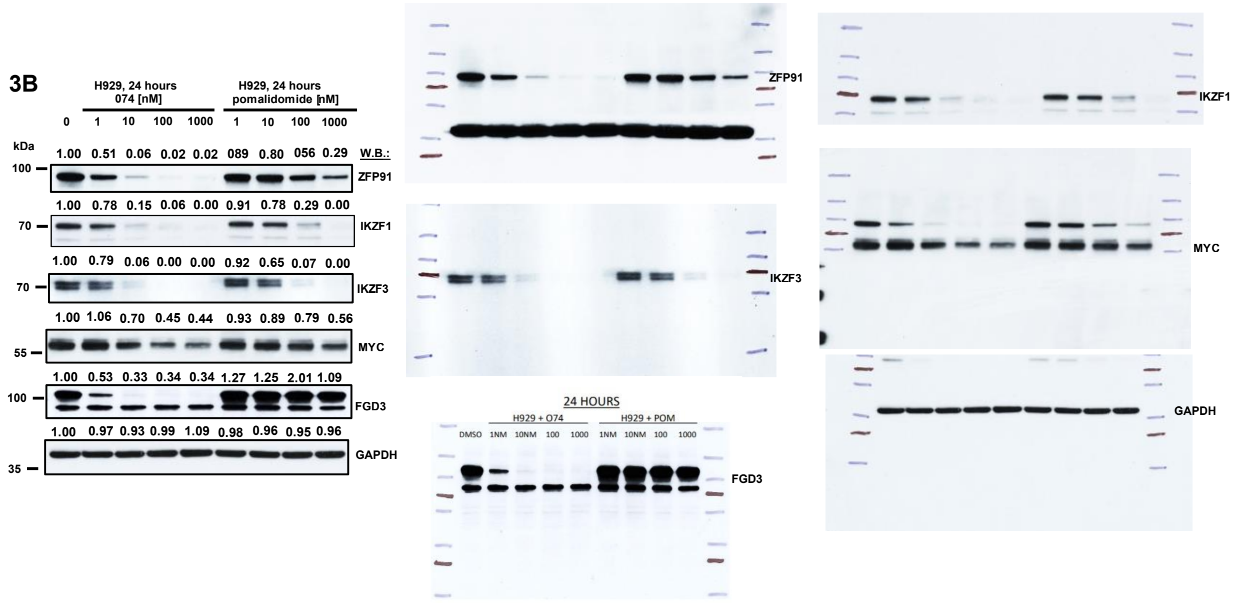

Figure S21 (part 1). Uncut gels/densitometry for Figure 3B.

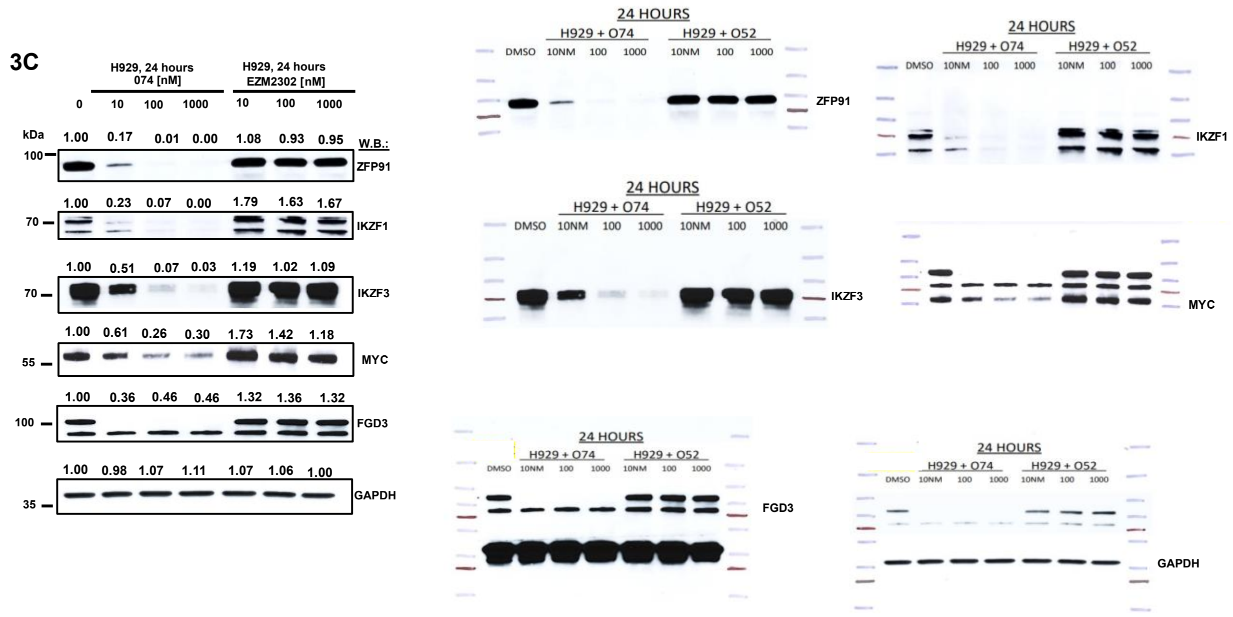

Figure S21 (part 2). Uncut gels/densitometry for Figure 3C.

Figure S10A

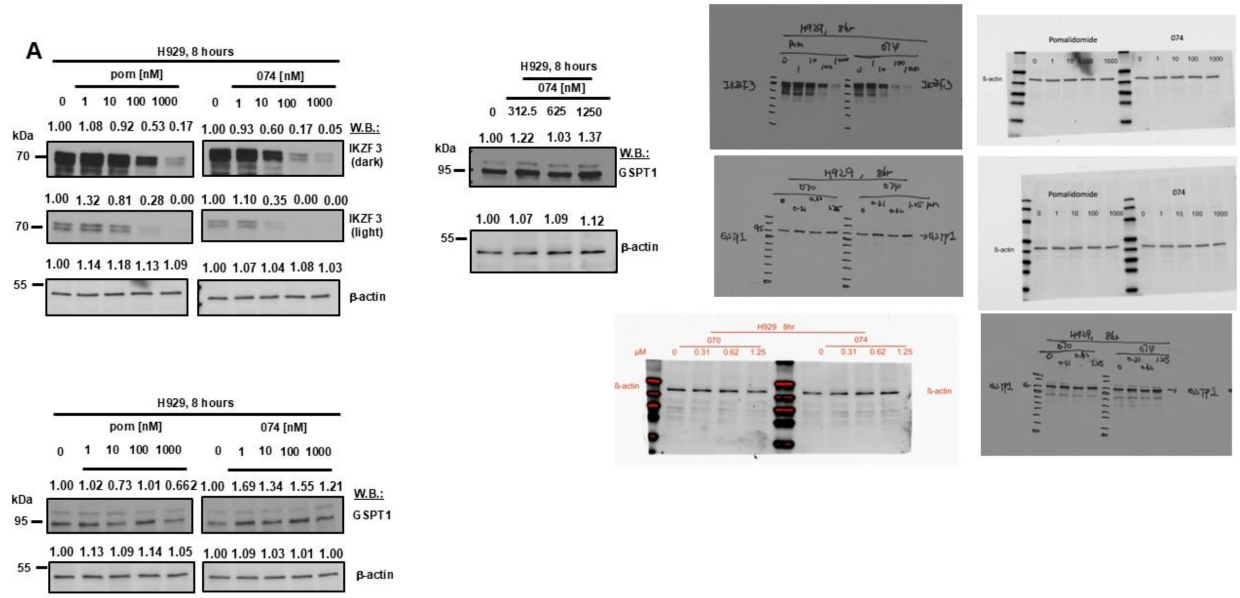

Figure S22 (part 1). Uncut gels/densitometry for Figure S10A.

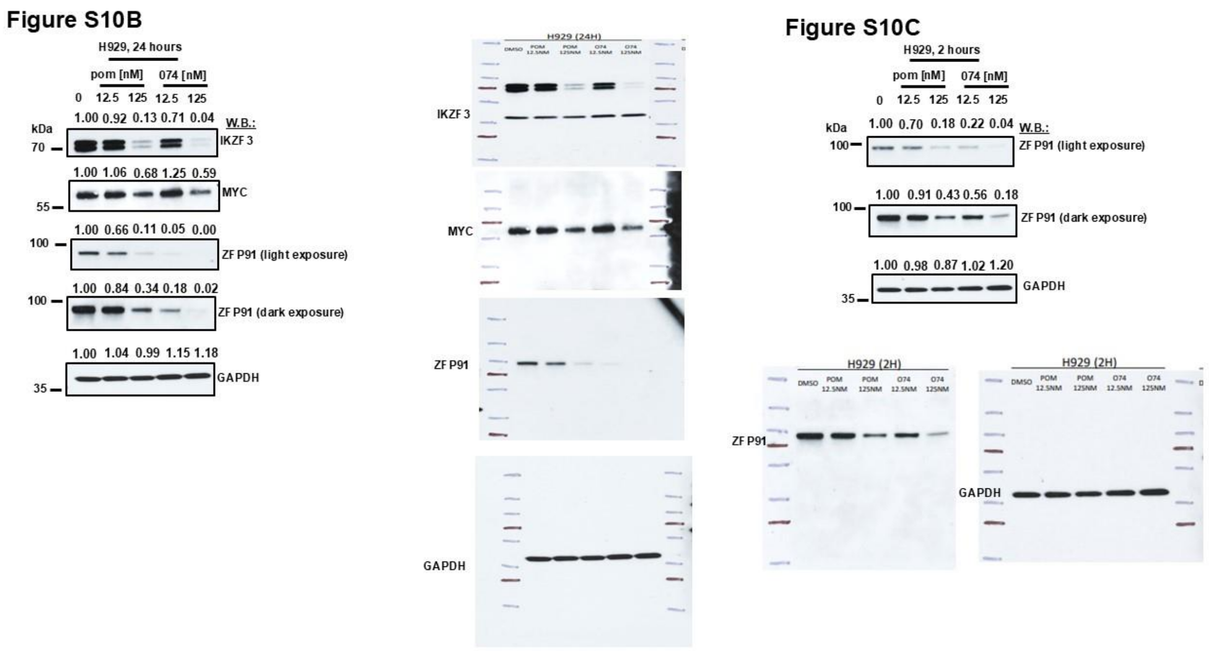

Figure S22 (part 2). Uncut gels/densitometry for Figure S10B and C.

Figure S10D

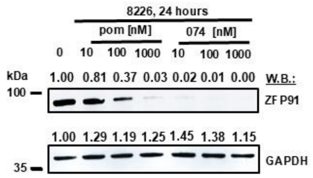

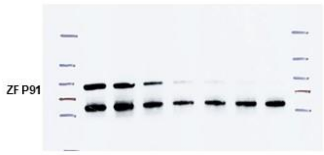

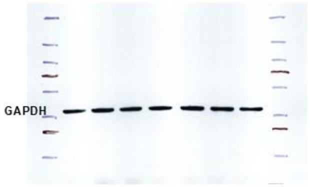

Figure S10E

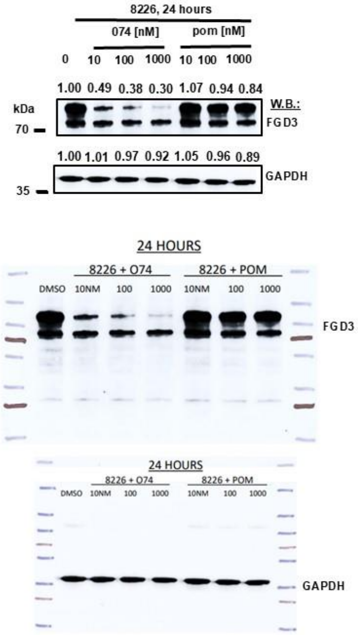

Figure S22 (part 3). Uncut gels/densitometry for Figure S10D and E.

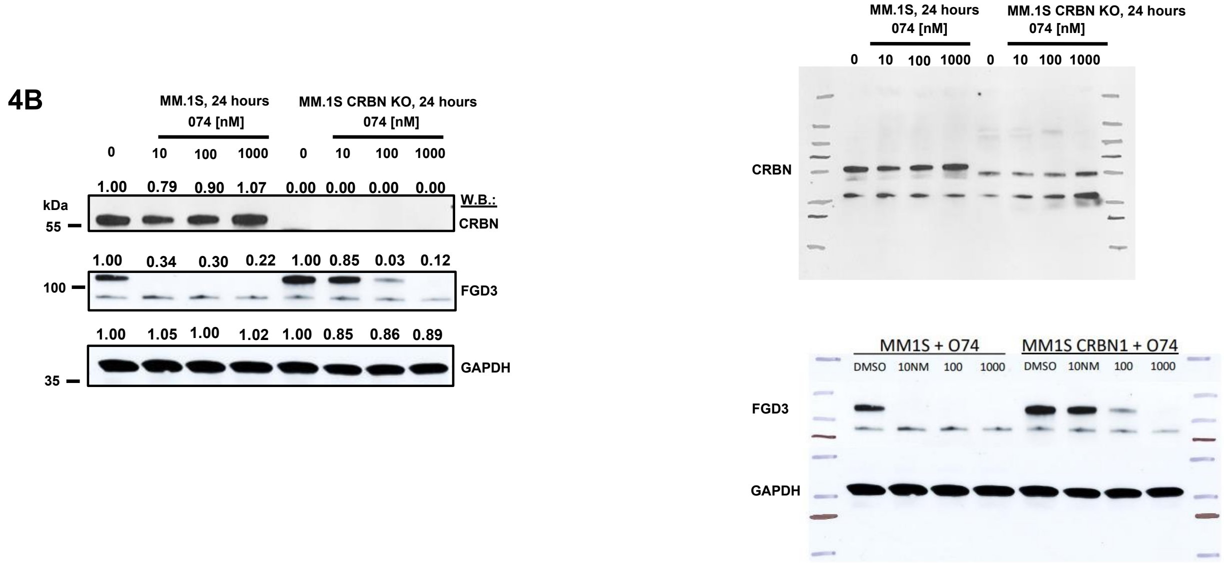

Figure S23 (part 1). Uncut gels/densitometry for Figure 4B.

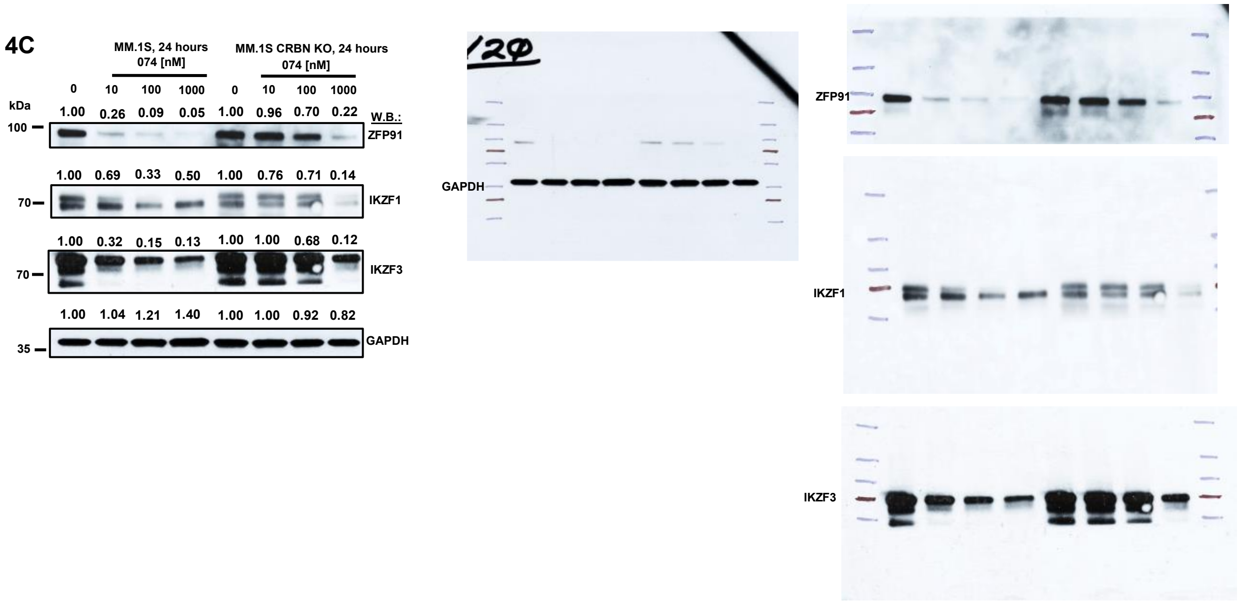

Figure S23 (part 2). Uncut gels/densitometry for Figure 4C.

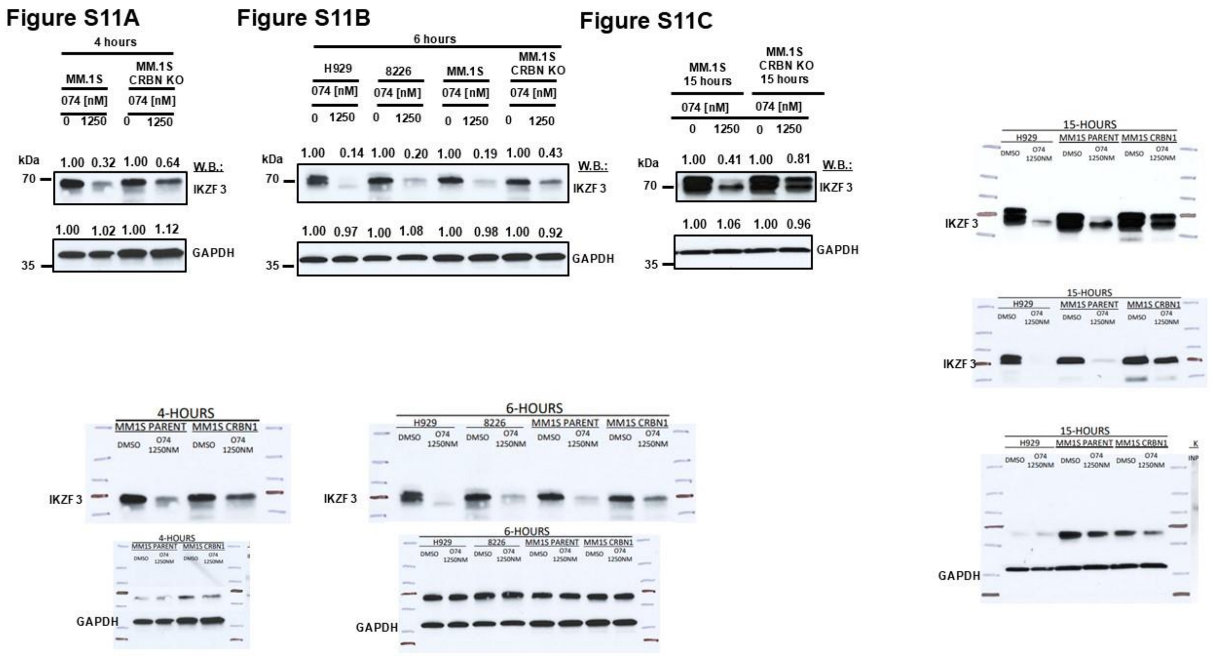

Figure S24 (part 1). Uncut gels/densitometry for Figure S11A-C.

Figure S11D

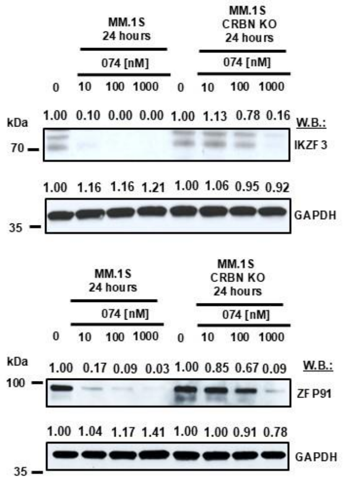

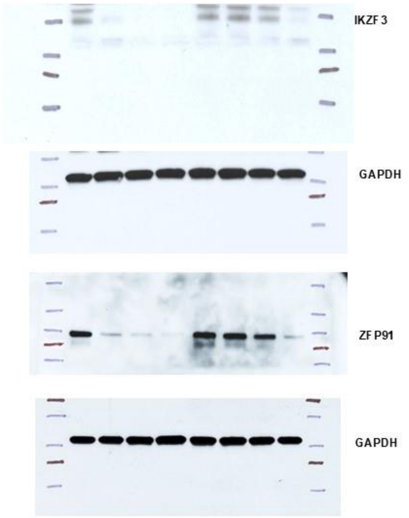

Figure S24 (part 2). Uncut gels/densitometry for Figure S11D.

Figure S11F

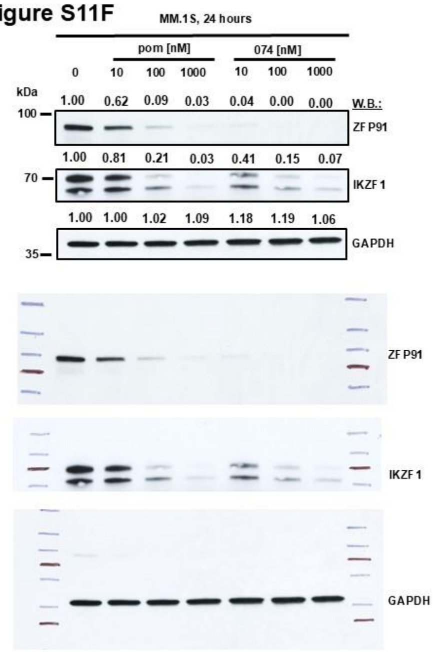

Figure S11G

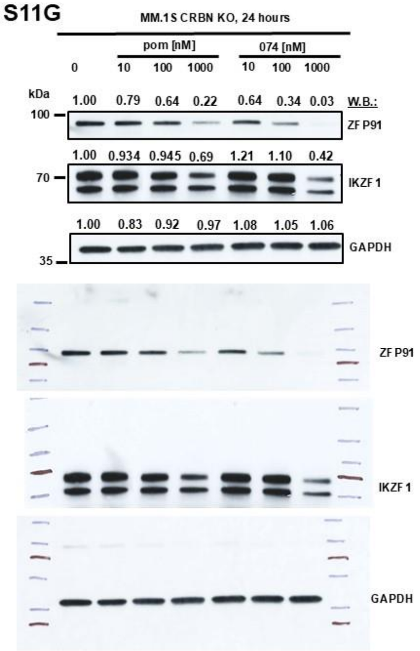

Figure S24 (part 3). Uncut gels/densitometry for Figure S11F and G.

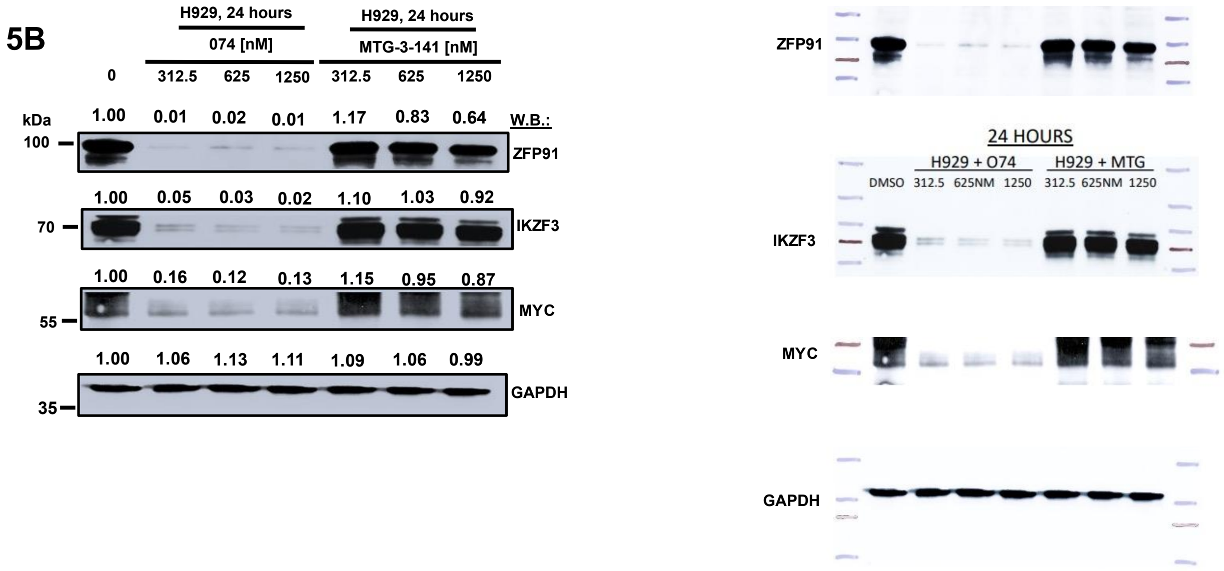

Figure S25 (part 1). Uncut gels/densitometry for Figure 5B.

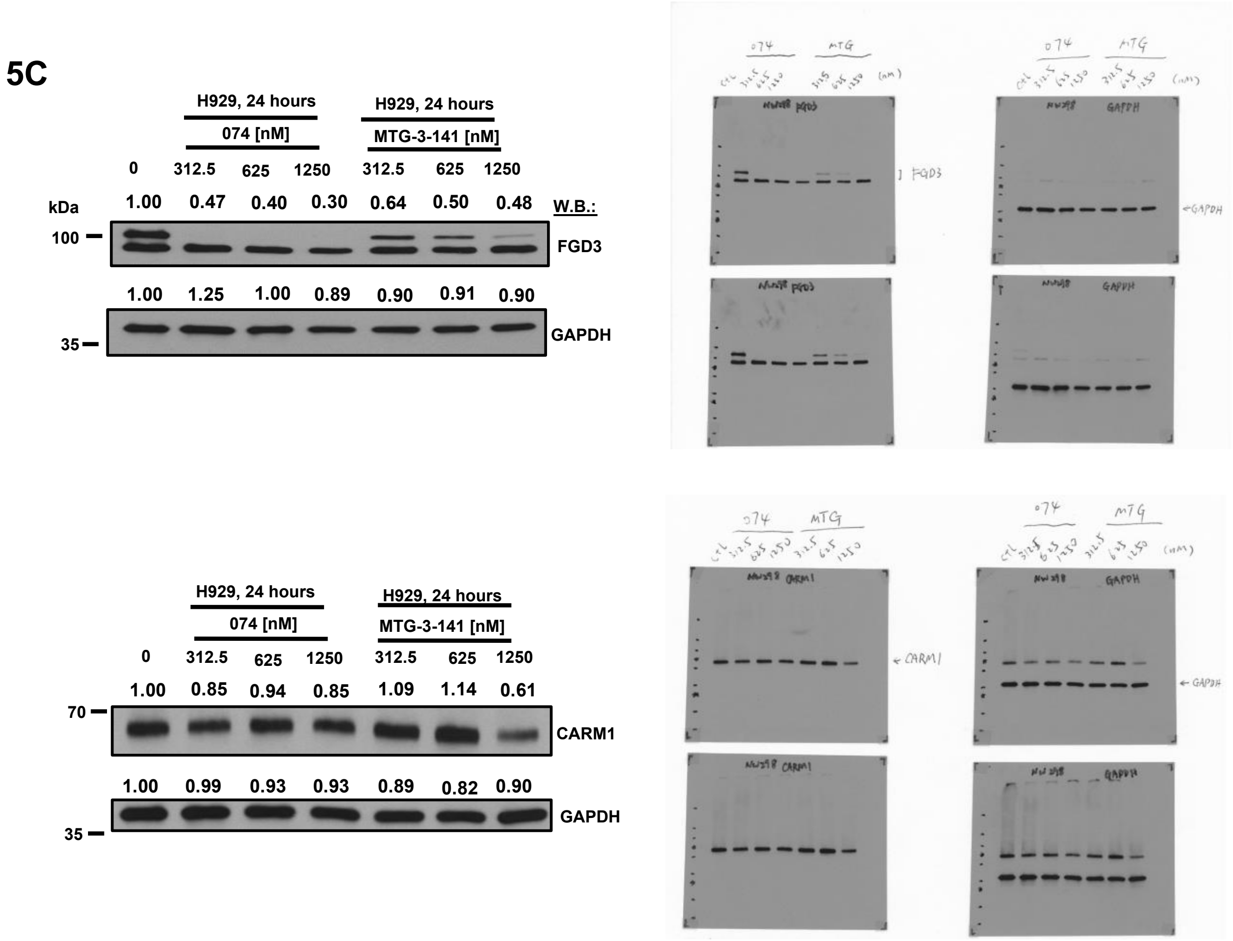

Figure S25 (part 2). Uncut gels/densitometry for Figure 5C.

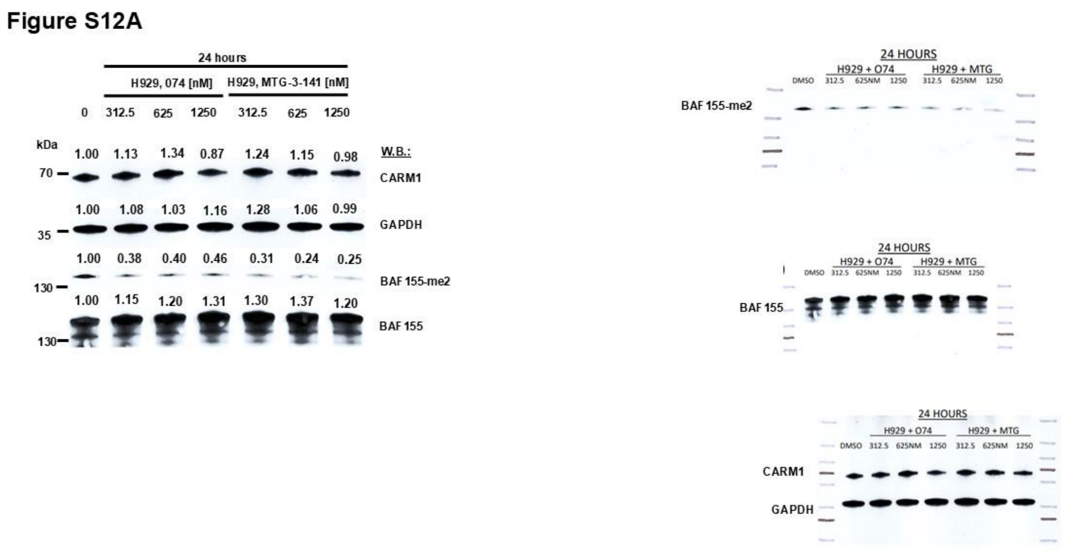

Figure S26 (part 1). Uncut gels/densitometry for Figure S12A.

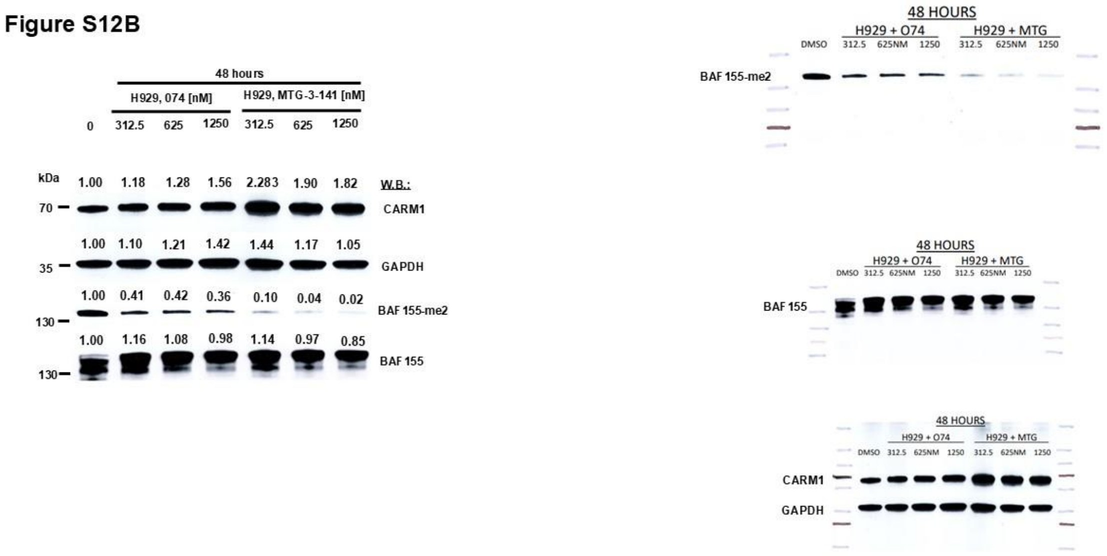

Figure S26 (part 2). Uncut gels/densitometry for Figure S12B.

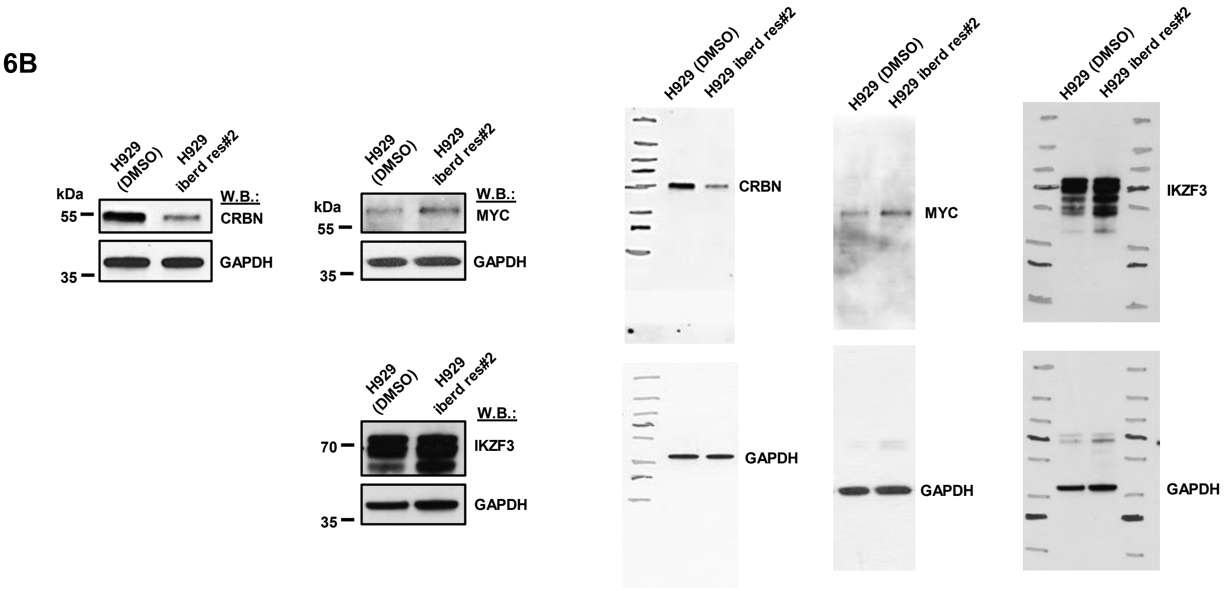

Figure S27. Uncut gels/densitometry for Figure 6B.

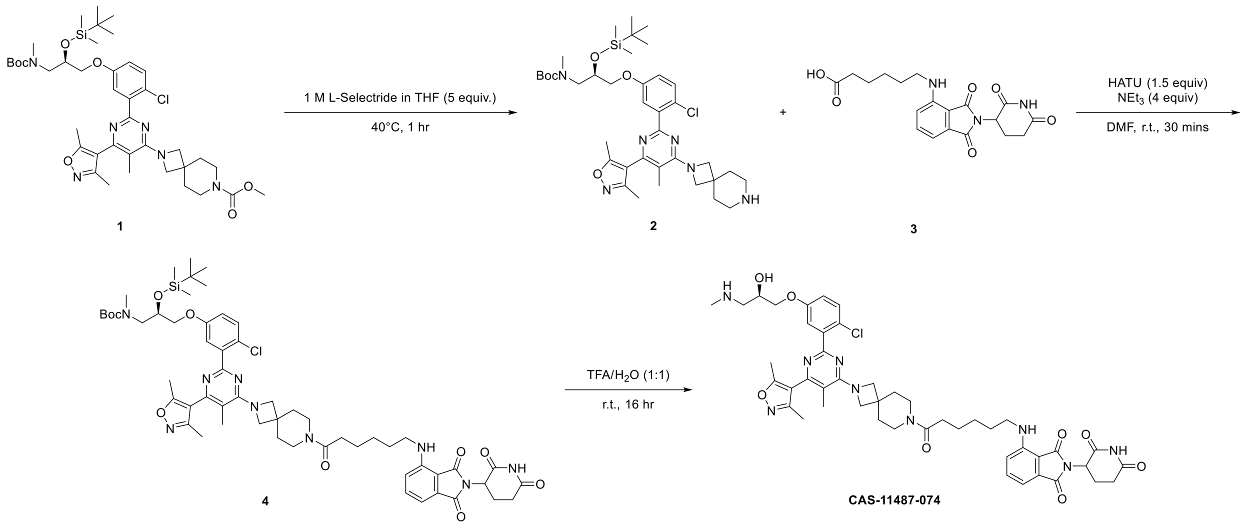

Figure S28. Synthetic Procedure and Characterization of CAS-11487-074.

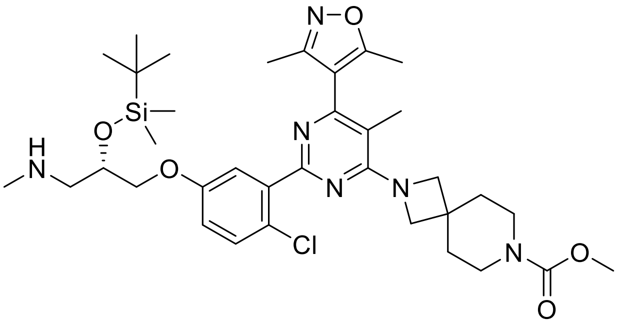

2

Figure S29. Intermediate 2. A borosilicate glass vial equipped with a magnetic stir bar was charged with a solution of 1 M L-Selectride in THF (2.28 mL, 2.28 mmol) and compound **1** (364 mg, 0.455 mmol). The solution was stirred at 40 °C for 1 hour. Upon completion, the reaction mixture was cooled to room temperature and concentrated under reduced pressure. The crude reaction product was then purified by flash chromatography (gradient from 20:80 to 90:10 methanol with 5% triethylamine: ethyl acetate) to afford the product as a clear liquid (289 mg, 86% yield). LCMS (ESI) $[M+H]^+$ calculated for $C_{38}H_{58}ClN_6O_5Si^+$m/z 742.45, found m/z 742.81.

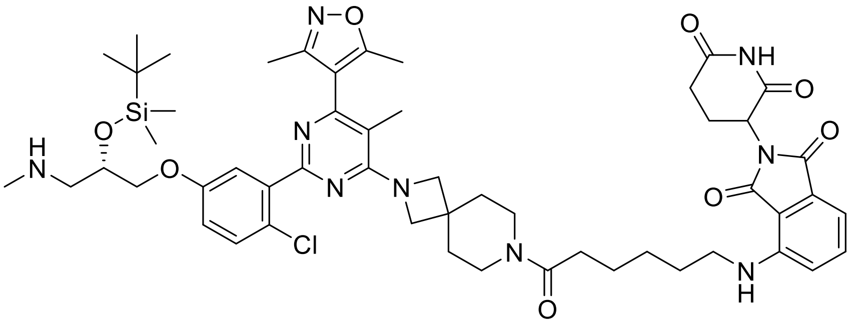

4

Figure S30. *tert*-Butyl ((2*R*)-2-((*tert*-butyldimethylsilyl)oxy)-3-(4-chloro-3-(4-(3,5-dimethylisoxazol-4-yl)-6-(7-(6-((2-(2,6-dioxopiperidin-3-yl)-1,3-dioxoisoindolin-4-yl)amino)hexanoyl)-2,7-diazaspiro[3.5]nonan-2-yl)-5-methylpyrimidin-2-yl)phenoxy)propyl)(methyl)carbamate (4). A borosilicate glass vial equipped with a magnetic stir bar was charged with **2** (65.8 mg, 0.0888 mmol) and **3** (34.4 mg, 0.0888 mmol). Dimethylformamide (3 mL) and triethylamine (50.0 µL, 0.355 mmol) were added followed by hexafluorophosphate azabenzotriazole tetramethyl uronium (50.6 mg, 0.133 mmol). The reaction mixture was stirred at room temperature for 30 minutes. Upon completion, the reaction mixture was purified by reverse-phase high-performance liquid chromatography to afford the product as yellow solid (26.2 mg, 27% yield). LCMS (ESI) $[M+H]^+$ calculated for $C_{57}H_{77}ClN_9O_{10}Si^+$m/z 1111.82, found m/z 1111.85.

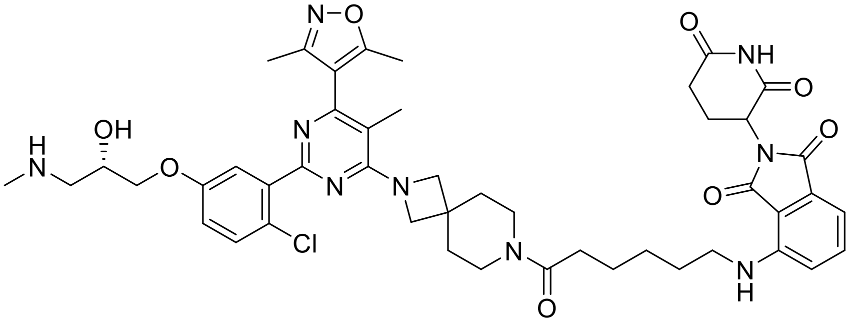

CAS-11487-074

Figure S31. 4-((6-(2-(2-(2-chloro-5-(®-2-Hydroxy-3-(methylamino)propoxy)phenyl)-6-(3,5-dimethylisoxazol-4-yl)-5-methylpyrimidin-4-yl)-2,7-diazaspiro[3.5]nonan-7-yl)-6-oxohexyl)amino)-2-(2,6-dioxopiperidin-3-yl)isoindoline-1,3-dione (CAS-11487-074).
A borosilicate glass vial equipped with a magnetic stir bar was charged with **4** (26.2 mg, 0.0236 mmol) and a solution of TFA/H_2O (1:1, 2 mL). The reaction mixture was stirred at room temperature for 16 hours. Upon completion, the reaction mixture was concentrated and purified by flash chromatography (gradient from 50:50 to 100:0 methanol with 5% triethylamine: ethyl acetate) to afford the product as a yellow solid (18.5 mg, 88% yield). LCMS (ESI) $[M+H]^+$ calculated for $C_{46}H_{55}ClN_9O_8^+$ m/z 896.44, found m/z 896.42. ^{1}H NMR (500 MHz, Chloroform-*d*) δ 7.52 – 7.45 (m, 1H), 7.31 (d, *J* = 8.8 Hz, 2H), 7.08 (d, *J* = 7.0 Hz, 1H), 6.88 (d, *J* = 8.6 Hz, 1H), 6.85 (dd, *J* = 8.8, 3.1 Hz, 2H), 6.25 – 6.19 (m, 1H), 4.94 – 4.86 (m, 1H), 4.32 – 4.26 (m, 1H), 4.11 (s, 4H), 4.07 – 4.01 (m, 1H), 3.99 – 3.93 (m, 1H), 3.64 (s, 7H), 3.64 – 3.57 (m, 3H), 3.45 (d, *J* = 4.8 Hz, 2H), 3.28 (dt, *J* = 13.8, 6.9 Hz, 2H), 3.11 – 2.66 (m, 9H), 2.64 (s, 2H), 2.37 (s, 3H), 2.27 (s, 3H), 2.11 (s, 3H), 1.53 – 1.44 (m, 3H), 1.38 (t, *J* = 7.2 Hz, 3H), 1.25 (s, 3H), 0.88 (t, *J* = 6.7 Hz, 1H). (missing 2 protons)

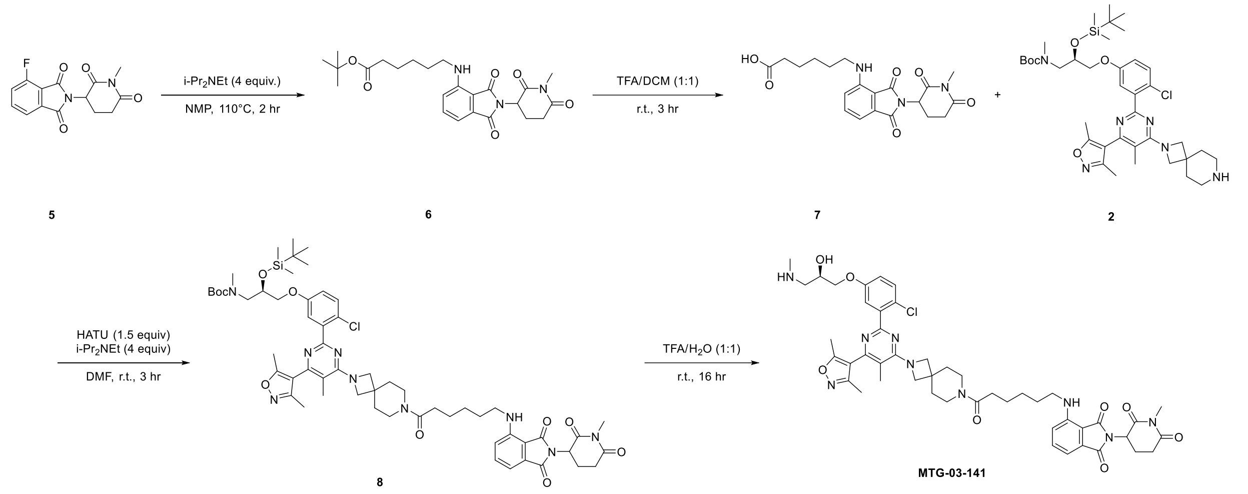

Figure S32. Synthetic Procedure and Characterization of MTG-03-141.

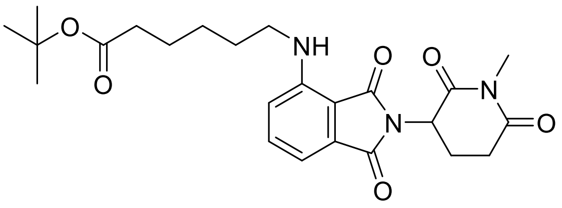

6

Figure S33. *tert*-Butyl 6-((2-(1-methyl-2,6-dioxopiperidin-3-yl)-1,3-dioxoisoindolin-4-yl)amino)hexanoate (6). To a stirred solution of **5** (142 mg, 0.487 mmol) in N-Methyl-2-Pyrrolidone (1 mL) was added N,N-Diisopropylethylamine (339 µL, 1.95 mmol) followed by *tert*-butyl 6-aminohexanoate (218 mg, 0.975 mmol). The reaction mixture was heated at 110 °C in a sand bath with stirring for 2 hours. Once complete, the reaction mixture was cooled to room temperature, resuspended in water (20 mL) and extracted with dichloromethane (3 x 20 mL). Organic layers were dried with sodium sulfate, filtered, concentrated, and purified by flash chromatography (gradient from 0:100 to 40:60 hexanes: ethyl acetate) to afford the product as a yellow oil (32.5 mg, 14% yield). LCMS (ESI) $[M+H]^+$ calculated for $C_{24}H_{32}N_3O_6^+$ - tBu m/z 402.43, found m/z 402.32.

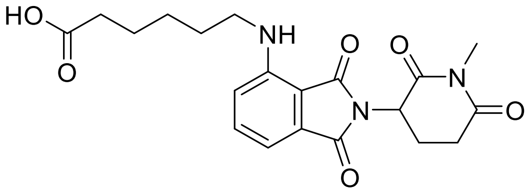

7

Figure S34. 6-((2-(1-methyl-2,6-dioxopiperidin-3-yl)-1,3-Dioxoisoindolin-4-yl)amino)hexanoic acid (7). A 1:1 solution of TFA/DCM (1 mL) was added to **6** (32.5 mg, 0.710 mmol) and stirred at room temperature for 3 hours. Once complete, the reaction mixture was concentrated to afford the crude product and used for subsequent reaction without further purification. LCMS (ESI) $[M+H]^+$ calculated for $C_{20}H_{24}N_3O_6^+$ m/z 402.42, found m/z 402.32.

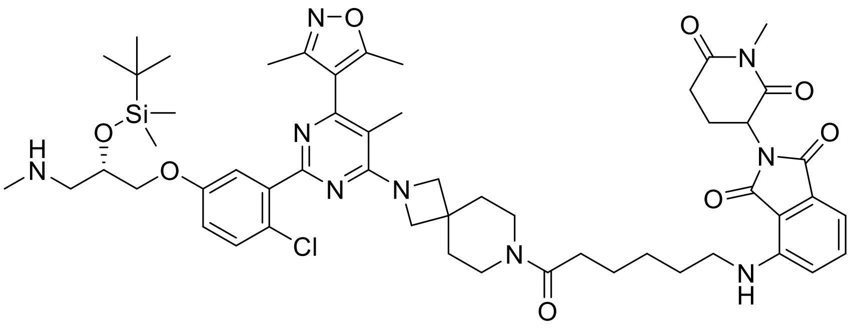

8

Figure S35. *tert*-Butyl ((2*R*)-2-((*tert*-butyldimethylsilyl)oxy)-3-(4-chloro-3-(4-(3,5-dimethylisoxazol-4-yl)-5-methyl-6-(7-(6-((2-(1-methyl-2,6-dioxopiperidin-3-yl)-1,3-dioxoisoindolin-4-yl)amino)hexanoyl)-2,7-diazaspiro[3.5]nonan-2-yl)pyrimidin-2-yl)phenoxy)propyl)(methyl)carbamate (8). To a stirred solution of **2** (57 mg, 0.0767 mmol) in dimethylformamide (1 mL) at room temperature was added **7** (31 mg, 0.0771 mmol) and N,N-Diisopropylethylamine (67.0 µL, 0.385 mmol) followed by hexafluorophosphate azabenzotriazole tetramethyl uronium (35.0 mg, 0.0925 mmol). After 3 hours, the reaction was resuspended in water (15 mL) and extracted with dichloromethane (2 x 20 mL). Organic layer was dried with sodium sulfate, filtered, concentrated, and purified by flash chromatography (gradient from 0:100 to 80:20 hexanes/ethyl acetate) to afford the product as a yellow oil (20.2 mg, 23.3% yield). LCMS (ESI) $[M+H]^+$ calculated for $C_{58}H_{79}ClN_9O_{10}Si^+$ m/z 1124.85, found m/z 1124.49.

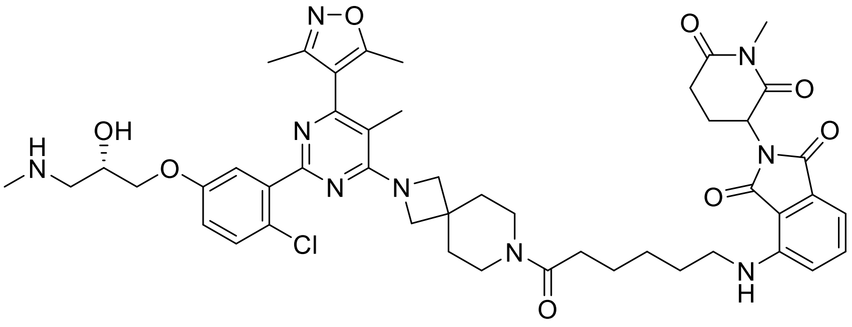

MTG-03-141

Figure S36. 4-((6-(2-(2-(2-chloro-5-((*R*)-2-Hydroxy-3-(methylamino)propoxy)phenyl)-6-(3,5-dimethylisoxazol-4-yl)-5-methylpyrimidin-4-yl)-2,7-diazaspiro[3.5]nonan-7-yl)-6-oxohexyl)amino)-2-(1-methyl-2,6-dioxopiperidin-3-yl)isoindoline-1,3-dione (MTG-03-141). A 1:1 solution of TFA/H2O (1 mL) was added to **8** (20.3 mg, 0.0180 mmol) and stirred at room temperature for 16 hours. Once completed, the reaction was concentrated and purified by reverse-phased high-performance liquid chromatography to afford the product as a yellow solid (10.1 mg,62% yield). LCMS (ESI) $[M+H]^+$ calculated for $C_{47}H_{57}ClN_9O_8^+$ m/z 910.47, found m/z 910.33.

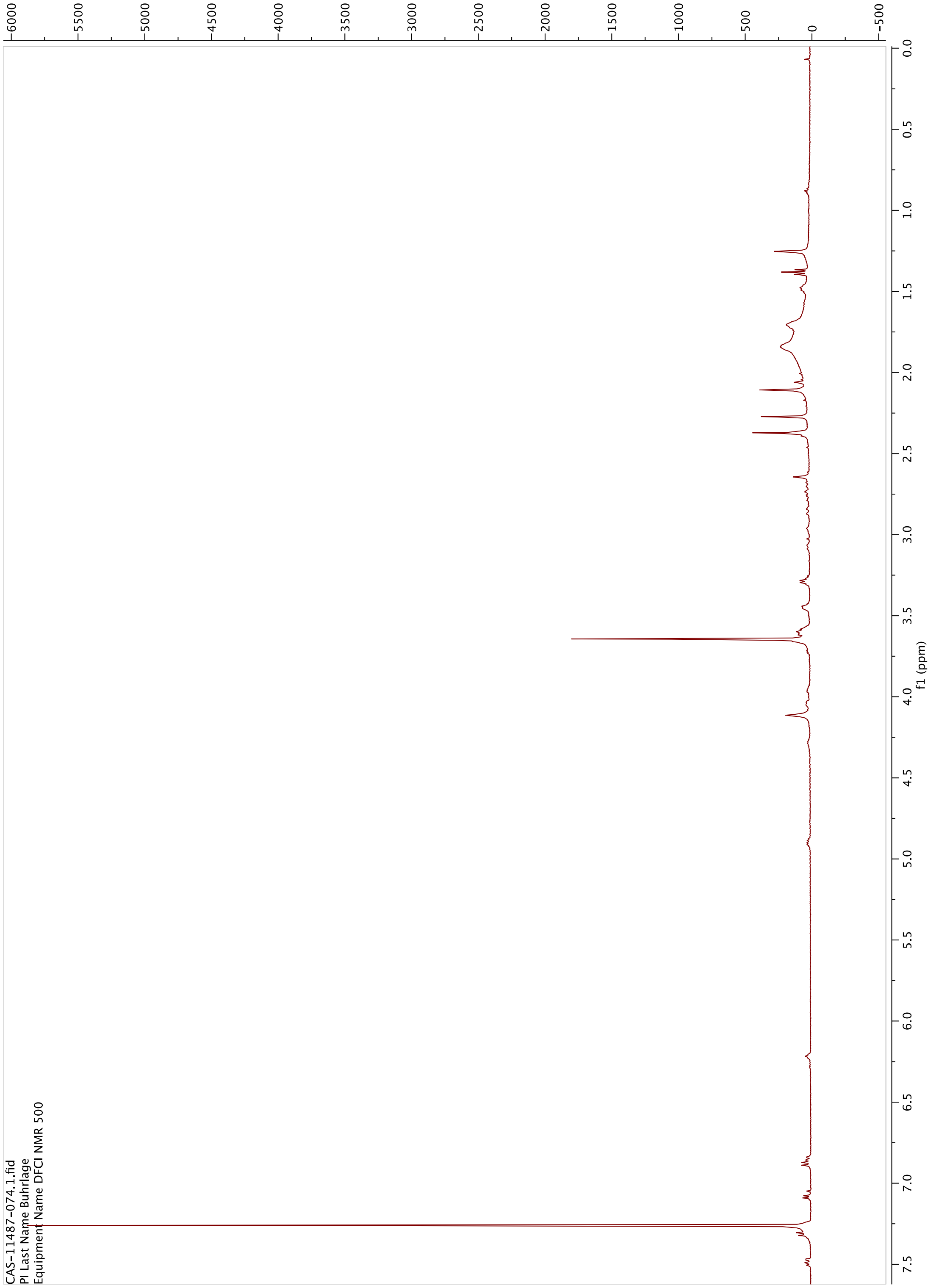

Figure S37. NMR spectrum of CAS-11487-074.

References:

1. Drew, A. E. et al. (2017). Identification of a CARM1 Inhibitor with Potent In Vitro and In Vivo Activity in Preclinical Models of Multiple Myeloma. *Scientific reports*, 7(1), 17993. https://doi-org.ezp-prod1.hul.harvard.edu/10.1038/s41598-017-18446-z
2. Patent: US10925868B2 Title: Degradation of protein kinases by conjugation of protein kinase inhibitors with E3 ligase ligand and methods of use
3. Posternak, G. et al. (2020). Functional characterization of a PROTAC directed against BRAF mutant V600E. *Nature chemical biology*, *16*(11), 1170–1178. https://doi-org.ezp-prod1.hul.harvard.edu/10.1038/s41589-020-0609-7
